# Deep genomic analysis of malignant peripheral nerve sheath tumor cell lines challenges current malignant peripheral nerve sheath tumor diagnosis

**DOI:** 10.1016/j.isci.2023.106096

**Published:** 2023-01-31

**Authors:** Miriam Magallón-Lorenz, Ernest Terribas, Sara Ortega-Bertran, Edgar Creus-Bachiller, Marco Fernández, Gerard Requena, Inma Rosas, Helena Mazuelas, Itziar Uriarte-Arrazola, Alex Negro, Tereza Lausová, Elisabeth Castellanos, Ignacio Blanco, George DeVries, Hiroyuki Kawashima, Eric Legius, Hilde Brems, Viktor Mautner, Lan Kluwe, Nancy Ratner, Margaret Wallace, Juana Fernández-Rodriguez, Conxi Lázaro, Jonathan A. Fletcher, David Reuss, Meritxell Carrió, Bernat Gel, Eduard Serra

**Affiliations:** 1 Hereditary Cancer Group, Germans Trias i Pujol Research Institute (IGTP), Can Ruti Campus, 08916 Badalona, Barcelona, Spain; 2 Hereditary Cancer Program, Catalan Institute of Oncology (ICO-IDIBELL), L'Hospitalet de Llobregat, 08098 Barcelona, Spain; 3 Program in Molecular Mechanisms and Experimental Therapy in Oncology (Oncobell), IDIBELL, Hospitalet de Llobregat, Barcelona, Spain; 4 Centro de Investigación Biomédica en Red de Cáncer (CIBERONC), Madrid, Spain; 5 Cytometry Core Facility, Germans Trias & Pujol Research Institute (IGTP), Badalona, Barcelona, Spain; 6 Clinical Genomics Research Group, Germans Trias i Pujol Research Institute (IGTP), Can Ruti Campus, 08916 Badalona, Barcelona, Spain; 7 Clinical Genomics Unit, Clinical Genetics Service, Northern Metropolitan Clinical Laboratory, Germans Trias i Pujol University Hospital (HGTP), Can Ruti Campus, 08916 Badalona, Barcelona, Spain; 8 Department of Neuropathology, Institute of Pathology, Heidelberg University Hospital, Heidelberg, Germany; 9 Clinical Cooperation Unit Neuropathology, German Cancer Research Center (DKFZ), German Consortium for Translational Cancer Research (DKTK), Heidelberg, Germany; 10 Genetic Counseling Unit, Clinical Genetics Service, Northern Metropolitan Clinical Laboratory, Hospital Universitari Germans Trias i Pujol, Badalona, Spain; 11 Hines VA Hospital, Hines, IL 60141, USA; 12 Division of Orthopedic Surgery, Department of Regenerative and Transplant Medicine, Niigata University Graduate School of Medical and Dental Sciences, Palliative Care Team, Niigata University Medical and Dental Hospital, Niigata, Japan; 13 Department of Human Genetics, KU Leuven, Leuven, Belgium; 14 Department of Neurology, University Medical Center Hamburg-Eppendorf, Hamburg, Germany; 15 Division of Experimental Hematology and Cancer Biology, Cincinnati Children’s Hospital Medical Center, Cincinnati, OH, USA; 16 Department of Molecular Genetics & Microbiology, and UF Health Cancer Center, University of Florida College of Medicine, Gainesville, FL, USA; 17 Department of Pathology, Brigham and Women’s Hospital, Harvard Medical School, 20 Shattuck Street, Thorn 528, Boston, MA 02115, USA; 18 Departament de Fonaments Clínics, Facultat de Medicina i Ciències de la Salut, Universitat de Barcelona (UB), 08036 Barcelona, Spain

**Keywords:** Biological sciences, Neuroscience, Cancer, Omics

## Abstract

Malignant peripheral nerve sheath tumors (MPNSTs) are soft-tissue sarcomas of the peripheral nervous system that develop either sporadically or in the context of neurofibromatosis type 1 (NF1). MPNST diagnosis can be challenging and treatment outcomes are poor. We present here a resource consisting of the genomic characterization of 9 widely used human MPNST cell lines for their use in translational research. NF1-related cell lines recapitulated primary MPNST copy number profiles, exhibited *NF1*, *CDKN2A*, and *SUZ12/EED* tumor suppressor gene (TSG) inactivation, and presented no gain-of-function mutations. In contrast, sporadic cell lines collectively displayed different TSG inactivation patterns and presented kinase-activating mutations, fusion genes, altered mutational frequencies and COSMIC signatures, and different methylome-based classifications. Cell lines re-classified as melanomas and other sarcomas exhibited a different drug-treatment response. Deep genomic analysis, methylome-based classification, and cell-identity marker expression, challenged the identity of common MPNST cell lines, opening an opportunity to revise MPNST differential diagnosis.

## Highlights

• A comprehensive genomic resource of widely used MPNST cell lines is presented
• Genomic, epigenomic, and marker information improve MPNST differential diagnosis
• Genomic analysis detected misidentified and misdiagnosed MPNST cell lines
• Misdiagnosed cell lines exhibited a different drug-treatment response

## Graphical abstract

**Figure:**
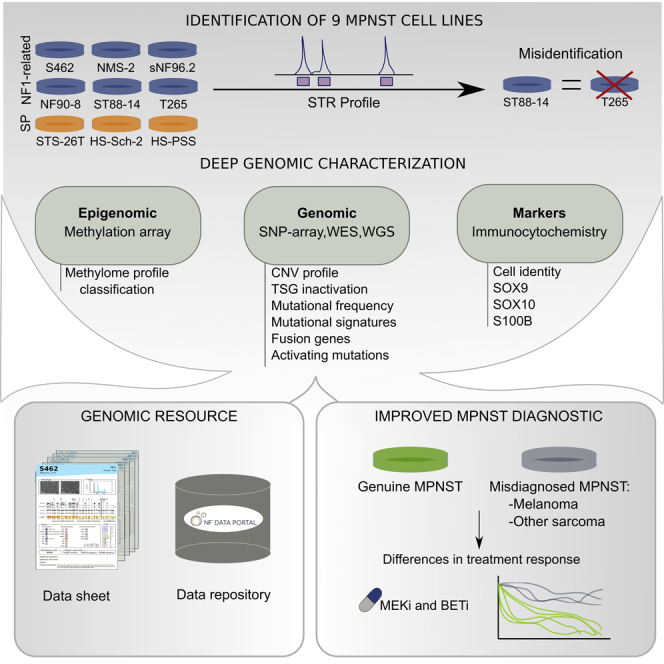

## Introduction

Malignant peripheral nerve sheath tumors (MPNSTs) are aggressive soft tissue sarcomas that arise from cells of the peripheral nervous system and account for 3-10% of all malignant soft tissue tumors.^1^ Half of these tumors develop in the context of the tumor predisposition syndrome Neurofibromatosis type 1 (NF1) while the other half are sporadic neoplasms.^2^^,^^3^ The MPNST incidence in the general population is 1 in 100,000^2^^,^^3^^,^^4^ whereas the lifetime risk of an NF1 individual developing an MPNST is 10-15%.^2^^,^^5^ Due to its invasive growth and propensity to metastasize, MPNSTs have a poor prognosis and are the leading cause of adult NF1-related mortality.^2^^,^^5^ Like many soft tissue sarcomas, complete resection with wide margins is essential in MPNST therapy, followed by radiation and/or chemotherapy.^6^^,^^7^^,^^8^

MPNSTs are usually high-grade malignant spindle cell neoplasms arising in association with large peripheral nerves.^9^ Their diagnosis can be challenging, especially outside of individuals with NF1, since MPNSTs are rare tumors and specific histological criteria have not been completely established.^10^^,^^11^^,^^12^ In the context of NF1, MPNSTs often progress from a pre-existing benign plexiform neurofibroma, commonly through an intermediate discrete nodular tumor termed atypical neurofibroma or ANNUBP.^12^^,^^13^ Although neurofibromas contain numerous S100B/SOX10-positive Schwann cells and CD34-positive fibroblasts, the expression of both markers is significantly reduced or absent in MPNSTs.^12^

MPNSTs contain hyperploid and highly rearranged genomes with a low mutation burden.^14^^,^^15^^,^^16^^,^^17^ Several tumor suppressor genes (TSGs) are commonly mutated, including *NF1*, *CDKN2A*, and components of the polycomb repressive complex 2 (PRC2), including *SUZ12* and *EED*. *TP53* is also frequently lost or mutated. MPNSTs also show recurrently altered chromosomal regions, particularly constituting somatic copy number gains (revised in Serra et al. 2020^18^). Complete loss of *CDKN2A*, often caused by structural alterations, seems to constitute a bottleneck for MPNST formation.^19^^,^^20^

Established cell lines are an important tool for gaining insight into cancer biology and treatment. However, there are also different caveats in their use as faithful and useful models, with issues including misidentification and cross-contamination and poor characterization of similarity to their tumor source.^21^^,^^22^^,^^23^^,^^24^ There is no dedicated registry for MPNST cell lines, but according to Cellosaurus (https://web.expasy.org/cellosaurus/), around forty different MPNST cell lines may have been established by different laboratories, derived from both sporadic and NF1-related MPNSTs. Some of these MPNST cell lines are well distributed among labs^25^^,^^26^ or deposited in global repositories (ATCC, RIKEN). These lines have been used as a primary tool for the identification of molecular pathways involved in MPNST pathogenesis,^27^^,^^28^ and served, for instance, for the identification of MEK inhibitors as useful therapeutic agents.^29^ Some can be engrafted in mice to generate genuine orthotopic MPNST tumors.^30^^,^^31^ However, a systematic and comprehensive genomic characterization of these MPNST cell lines is still missing, limiting the use of these cell lines for precision medicine or pharmacogenomic studies.

In this work, we performed a deep genomic characterization of 8 commonly used MPNST cell lines,^25^^,^^26^ identifying heterogeneity regarding the structure of the genome, the inactivation of tumor suppressor genes, the frequency of mutations, the mutational signatures, and the presence of gain-of-function mutations, especially among sporadic MPNST cell lines. This characterization challenged the identity of the sporadic MPNST cell lines studied and prompted us to use a methylome sarcoma classifier and to perform immunofluorescence of known cell identity markers. Our results, in addition to providing a valuable resource, uncover the necessity of systematically analyzing MPNSTs, combining pathology with genomic and molecular results, for improved differential diagnosis and classification of these malignancies.

## Results

### A genomic resource for neurofibromatosis type 1-associated and sporadic malignant peripheral nerve sheath tumor cell lines

We started with 9 NF1-associated and sporadic MPNST cell lines for a short tandem repeat (STR) authentication analysis and a comprehensive genomic characterization. We first performed an interspecies PCR of all cell lines to identify any possible interspecies cross-contamination (Data S1). Then we performed a human STR authentication analysis to identify any possible cross-contamination or misidentification among cell lines of human origin (Table S1). All STR profiles matched the STR profiles published in Cellosaurus and ATCC when available. However, in this process, we identified the same STR profile for ST88-14 and T265 cell lines (Data S2) in all ST88-14- and T265-related samples provided by different laboratories. To find out which cell line was misidentified we analyzed the oldest ST88-14 and T265 stored vials in their original labs and more conclusively, the primary tumor from which the ST88-14 cell line was isolated (Data S2). We identified the ST88-14 cell line as the genuine cell line for that STR profile, *NF1* germline (c.1649dupT) mutation and somatic copy number alteration landscape, and dismissed the use of the T265 cell line, which we assume was misidentified at some point after its establishment and expansion. Note that ST88-14 *NF1* germline mutation is not correctly described in certain repositories and publications.

We performed a comprehensive genomic characterization of the remaining 8 MPNST cell lines. Table 1 summarizes information on patients and MPNSTs from which cell lines were established and on their *NF1* mutational status. It also provides a reference to the original description of each cell line. With them, we performed flow cytometry, SNP-array analysis, whole-exome sequencing, and whole-genome sequencing techniques. We compiled information about their ploidy, global copy-number profile and loss of heterozygosity (LOH) status, structural rearrangements, single nucleotide variants (SNVs), and mutational signatures, and summarized the mutational status of a set of selected MPNST-related genes. With all these data we elaborated a practical summary sheet for each cell line, containing the most relevant information (Data S3).

**Table 1:** General description of the eight MPNST cell lines analyzed

	S462	ST88-14	NF90-8	sNF96.2	NMS-2	STS-26T	HS-Sch-2	HS-PSS
Type of human MPNST	primary, grade IV	primary	primary, from PNF	primary	primary	metastasis, grade III/III	primary, low grade	UNK
MPNST localization	thigh	retroperitoneum	left forearm	leg	right thigh	left scapula	left thigh	prostate
NF1/Sp	NF1	NF1	NF1	NF1	NF1	Sp	Sp	Sp
Age of patient	19	24	17	27	30	51	54	UNK
Sex of patient	F	M	F	M	M	F	F	M
Original Reference	Frahm-2004	Fletcher-1991	Legius-1994	Perrin-2007	Imaizumi-1998	Dahlberg-1993	Sonobe-2000	n/a
*NF1* constitutional pathogenic variant	c.6855C>A p.Y2264X Frahm-2004	c.1649dupT p.V551Gfs∗7 Varin-2016	c.3904_3910del p.D1302Yfs∗5 Wu-1999	c.3683delC p.N1229Mfs∗11 Perrin-2007	**c.6999+1G>T (splice donor)**			
*NF1* somatic pathogenic variant	LOH	LOH	LOH	LOH	**LOH**	**LOH**	**c.270_288del** **p.E91Nfs∗6; c.3113+1G>A (splice donor)**	

MPNST: Malignant peripheral nerve sheath tumor; NF1/Sp: NF1-related/Sporadic MPNST; F:Female; M: Male; Bold annotations: mutations described in this article; LOH: Loss of Heterozygosity; UNK: Unknown.

### A plethora of different ploidies

**Figure 1:**
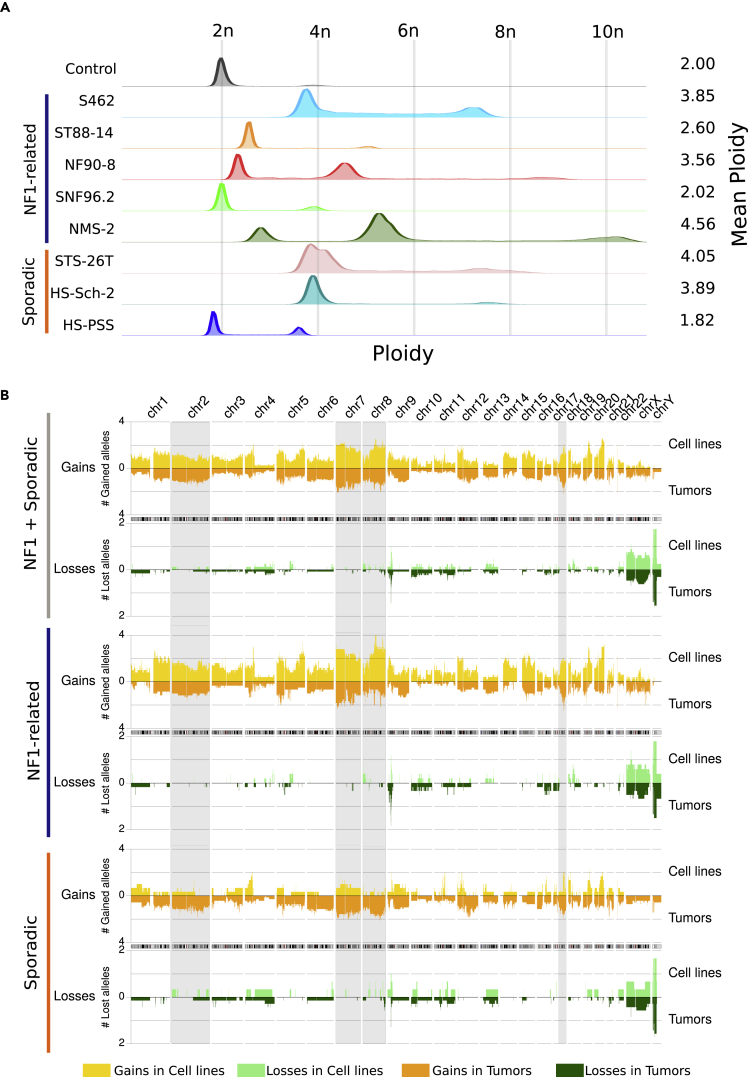
MPNST cell lines have different ploidies and recapitulate the genomic copy-number profile of primary MPNSTs (A) Ploidy status of MPNST cell lines obtained by flow cytometry. Each row represents a cell line, with the mean ploidy shown on the right side. HFF were used as 2n control cells. (B) Aggregated copy number profiles comparing MPNST cell lines and tumors. Y axis represents the mean number of gained (orange) or lost (green) alleles by sample type and Y axis genomic position. Cell lines are plotted in the upper part of each graph, represented by light colors, and tumors in the lower part, represented by dark colors and with inverted Y axis. Three different groups of samples are represented. From top to bottom: all MPNSTs; NF1-associated MPNSTs; and sporadic MPNSTs.

We first intended to characterize the karyotype of each MPNST cell line by spectral karyotyping (SKY) and G-banding staining. Both techniques produced results difficult to summarize consistently (data not shown), possibly due to the high degree of variability when analyzing multiple metaphases from the same cell line and to the highly rearranged nature of MPNST genomes. Therefore, we decided to analyze the ploidy by propidium iodide staining analysis using flow cytometry (Figure 1A), which revealed a striking diversity among the 8 MPNST cell lines. Most of them exhibited ploidies higher than 2n, three clearly around 4n (S462, HS-Sch-2, STS-26T), yet two cell lines were 2n or less (sNF96.2, HS-PSS). In addition, two cell lines, NF90-8 and NMS-2, showed two cell populations with different ploidy, resembling the result of a genome duplication event in the population with higher ploidy (Figure 1A). STS-26T cell line was composed of two subpopulations with slightly different ploidies. The mean ploidy of each cell line was also calculated considering this diversity.

### Malignant peripheral nerve sheath tumor cell lines faithfully recapitulate the genomic copy number profile of primary malignant peripheral nerve sheath tumors

Next, we performed a global copy number analysis using SNP-array data from the 8 MPNST cell lines. To verify whether these cell lines were capturing the copy number profiles present in primary MPNST tumors, we also analyzed SNP-array data from an independent set of primary MPNSTs previously analyzed,^19^ and compared the genomic profiles of cell lines and tumors (Figure 1B). Taken together, the copy number profiles of the 8 MPNST cell lines recapitulated fairly well those present in primary MPNSTs. The most recurrently gained genomic regions were chromosomes 7, 8, and 17q; and chromosome 9p was the most recurrently lost genomic region. When we separated NF1-related and sporadic MPNSTs, NF1-related cell lines mostly maintained these profiles and recurrences, but the three sporadic cell lines (Figure 1B) differ substantially in many genomic regions (see for example shaded gray regions in Figure 1B).

**Figure 2:**
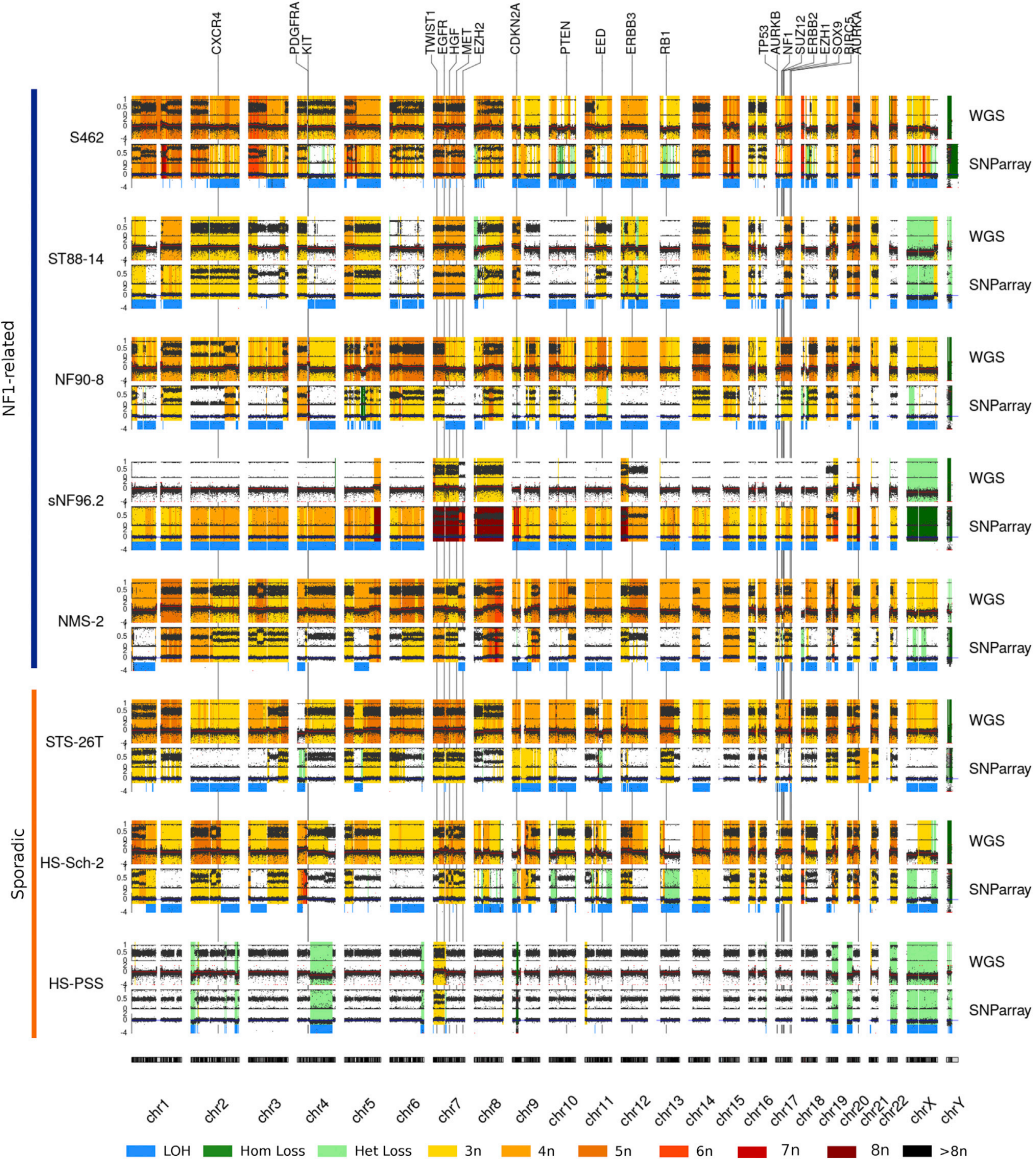
Global view of the copy number profiles of all MPNST cell lines studied Copy number profiles from SNP-array and WGS data. For both technologies, B-allele frequency (BAF) and Log-R Ratio (LRR) are represented (see STAR Methods for details). Warm colors represent copy number gains, whereas light green indicates a heterozygous loss and dark green a homozygous loss. Loss of heterozygosity (LOH) detected by using SNP-array data is highlighted by a thick blue line. The genomic location of MPNST-associated genes is indicated by a vertical black line with the gene symbol at the upper part of the graph. See Data S3 for a high-resolution profile of each cell line, chromosome by chromosome.

We also performed a copy number analysis from all cell lines based on WGS data. In addition, we obtained a B-allele frequency (BAF)-like profile from variant-allele frequencies (VAF) and a log-R ratio (LRR) from coverage. We plotted them together with the resulting LOH determination and copy number calling from SNP-array comparing both independent sets of data (Figure 2; Data S4 for a high-resolution profile). The use of both technologies for generating the same data allowed the validation of the results with all cell lines displaying similar BAF and LRR profiles. It also underscored the difficulty of using copy number callers for analyzing the highly altered MPNST genomes, indicating the necessity of estimating copy number changes in MPNSTs by different means. In addition to its known hyperploid genome, our analysis identified a high degree of generalized LOH (e.g.: almost the entire genome of the sNF96.2 cell line), perhaps pointing to the inactivation of TSGs before the gain of chromosomal regions. Finally, it must be noted that the copy number profile of a given MPNST cell line differed a bit from lab to lab, in terms of a certain number of different genomic alterations. By comparing the genomic profiles of different batches of the same cell lines obtained from distinct labs, we found that despite being quite stable, genomes of MPNST cell lines can accumulate changes in chromosomal regions (Data S5). Part of this inter-laboratory variability could be removed after growing the cell line as a xenograft in mice^31^ when growth selective pressure is applied.

### Structural variants as key players for tumor suppressor gene inactivation

**Figure 3:**
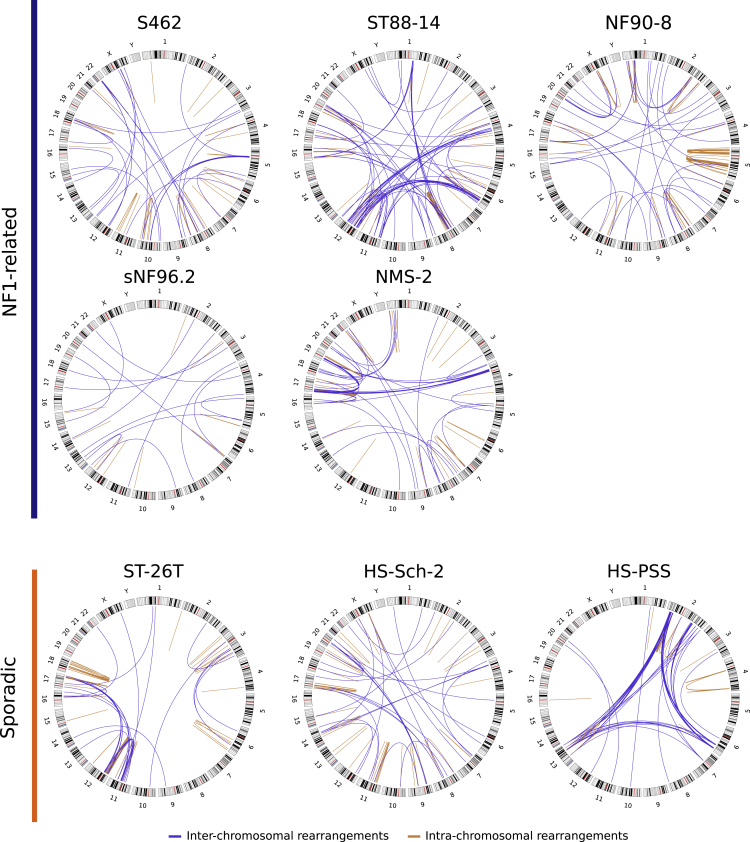
Structural rearrangements as key players for tumor suppressor gene inactivation Circos plots showing the chromosomal rearrangements identified in the different MPNST cell lines. Blue lines represent inter-chromosomal rearrangements and orange lines intra-chromosomal rearrangements. These rearrangements were obtained using Lumpy and CliffHunteR on WGS data.

Ploidy and copy number profile comprise a partial description of the genomic status of MPNST cell lines. We also took advantage of WGS data to analyze the presence of structural variants, represented by different types of chromosomal rearrangements (Figure 3). This analysis revealed that in addition to being hyperploid, MPNST genomes were highly rearranged, adding an extra layer of complexity. Structural changes were spread over all chromosomes, although certain cell lines such as ST88-14 and HS-PSS showed some genomic regions with a high frequency of adjacent rearrangements. Importantly, as previously reported,^19^ structural changes caused the inactivation of key MPNST TSGs, such as *CDKN2A*, PRC2 genes, and *TP53* (Data S6), that had been previously missed when WGS data was not available. Furthermore, WGS also facilitated the unexpected identification of a translocation resulting in the generation of a fusion gene EML4-ALK variant 5a^32^ in the HS-PSS cell line, which was validated by RT-PCR, amplification, and sequencing (Data S6). The significant number of structural variants identified together with the copy number profiles and ploidy exhibited, demonstrates the complex genomic landscape of MPNST cell lines, with highly altered but also fairly stable genomes.

### The limited importance of small genomic variants

**Figure 4:**
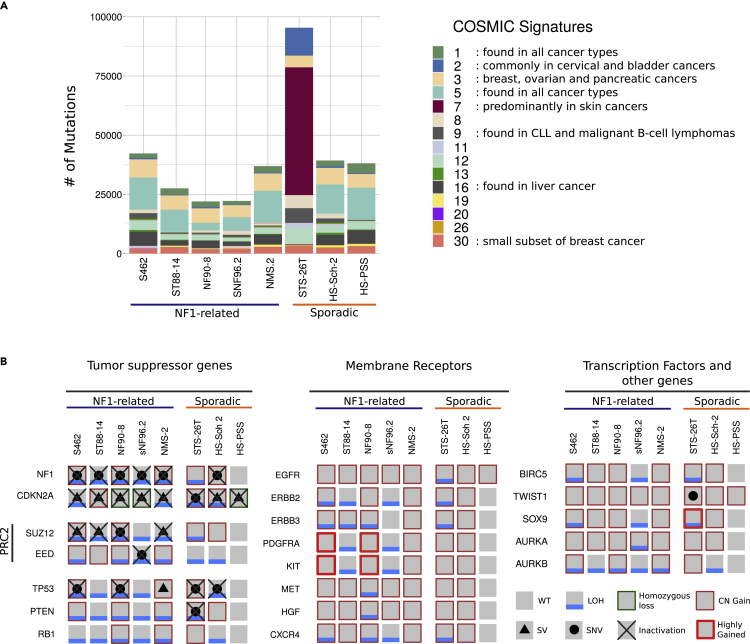
Small genomic variants have limited importance in altering MPNST-associated genes (A) Mutation number and estimated contribution of COSMIC mutational signatures in MPNST cell lines. Each bar represents the total number of somatically enriched single nucleotide variants (SNVs) found in an MPNST cell line. Each color represents a different COSMIC mutational signature. (B) Status of the commonly altered genes in MPNSTs. A gray square represents a wild type (WT) status of the gene; a blue line indicates the presence of loss of heterozygosity (LOH) detected by using SNP-array data; a black dot a small nucleotide variant affecting the gene; a black triangle indicates that the gene is affected by a structural variant (SV); copy number gain (CN gain) is represented by a thin red square; and a thick red square denotes a highly gained region affecting the gene. A homozygous loss of a gene is specified by a thin dark green square and the complete inactivation of a gene is represented by a black cross. Note that all NF1-related MPNST cell lines have the complete inactivation of *NF1*, *CDKN2A* and the *PRC2* complex. In addition, *CDKN2A* is also inactivated in sporadic cell lines.

In contrast to the rich number of gross structural alterations, fine-scale analysis of small variants, including single-nucleotide variants (SNVs) and small indels, uncovered a relatively moderate impact of these alterations in MPNSTs. We used WES and WGS data from all cell lines to call small variants. Since we did not have available non-tumoral tissue counterparts for these cell lines, we filtered the small variants to partially remove germline variants and obtain a call set enriched in somatic variants. The number of variants with a potential impact on protein function in this dataset was modest (Table S2), particularly for NF1-associated MPNST cell lines, which harbored a mean of 129 SNVs. We then used this quasi-somatic variant dataset to estimate the contribution of the COSMIC mutational signatures^33^ in the variant profile of the MPNST cell lines (Figure 4A). This analysis did not identify a particular mutational mechanism prevalent in MPNSTs and showed a major contribution of clock-like signatures (signatures 1 and 5), comparable with previous observations in other sarcomas.^17^ However, the STS-26T cell line in addition to exhibiting a higher number of mutations compared to the other cell lines (about two times the average of the rest of the cell lines), also presented an important contribution from signature 7, predominantly found in skin cancers (see later in discussion).

The functional impact of small variants in oncogenes and TSGs was also moderate. We identified some MPNST-related genes inactivated by pathogenic SNVs (Figure 4B and Table S3). In addition to germline *NF1* mutations, somatic mutations also affected *NF1*, as well as other genes including *TP53*, PRC2 genes, and *PTEN*. Remarkably, we did not identify gain-of-function mutations in oncogenes, except a *BRAF* V600E mutation in the STS-26T cell line. In contrast, we identified gains in genomic regions containing receptors, especially a highly gained region containing *PDGFRA* and *KIT* in two NF1-related cell lines (S462 and NF90-8) (Figure 4B). The most frequently inactivated gene in our set of cell lines was *CDKN2A*, a known bottleneck for MPNST development.^19^^,^^20^ The fact that this gene was inactivated by a point mutation only in one cell line, exemplifies the relatively low functional impact of small variants compared to structural variants in MPNST initiation.

### The combination of genome, methylome, and expression marker analysis, represent useful tools for a better differential diagnosis and classification of malignant peripheral nerve sheath tumors

**Figure 5:**
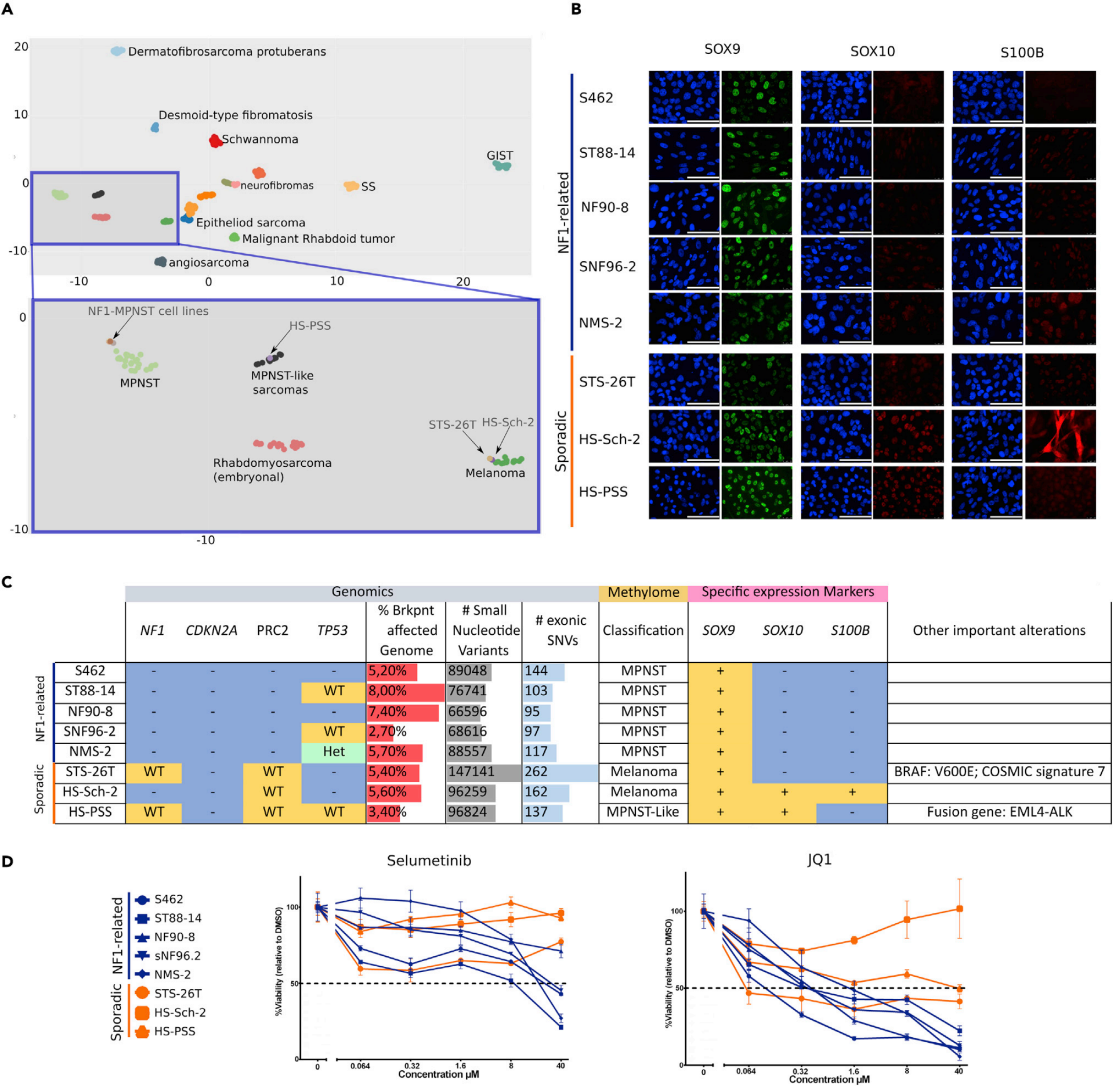
Genome, methylome, and marker analysis: useful tools for a better differential diagnosis and MPNST classification (A) UMAP analysis of the methylome profile of the MPNST cell lines in comparison to different soft tissue tumor types.^34^ The upper part of the plot provides a global view of the classification. Each dot represents a sample and every color is a different sarcoma type. The lower part of the graph is an inset magnification of a specific part containing all the MPNST cell lines analyzed. All NF1-related MPNST cell lines group within the MPNST methylation group (light green). HS-PSS cell line clustered with the MPNST-like sarcomas group (black) and STS-26T and HS-Sch-2 clustered together with the melanoma group (dark green). (B) Immunofluorescence images showing expression of SOX9, SOX10, and S100B markers in the different cell lines. DAPI was used to stain nuclei. Scale bars: 100μm. (C) Summary table of the genomic, methylome, and marker expression status of all MPNST cell lines. Genomics contains the status of *NF1*, *CDKN2A*, PRC2, and *TP53* genes (blue, complete gene inactivation (−); yellow wild type (WT); light green heterozygous deletion (Het)); percentage (%) of breakpoints affecting the genome +/− 1 mb; the number (#) of small nucleotide variants per sample; the # of exonic small nucleotide variants with a potential impact on protein function. Methylome contains the methylome-based classification of each cell line. Expression markers contain the expression of SOX9, SOX10, and S100B identity markers (yellow for expression (+); blue for absence (−). The “other important information” column summarizes additional relevant information identified in each cell line. (D) Cell viability assay upon treatment with different concentrations of Selumetinib and JQ1 of the NF1-related cell lines (blue) and the sporadic cell lines (orange). Each dot represents the mean of three independent experiments and error bars represent the standard deviation. The percentage of cell viability was calculated by normalizing the values to DMSO control cells.

This complete description of the genomic status of MPNST cell lines uncovered a fair degree of diversity among them, prompting us to question the MPNST identity of the sporadic cell lines and to perform a further characterization. Since sarcomas comprise a morphologically heterogeneous class of tumors and their diagnosis has been hampered by a high misclassification rate, we decided to perform methylome analysis of all cell lines in comparison to established reference cohorts of different peripheral nerve sheath and soft tissue tumors^34^ to clarify whether there was a problem in the diagnosis of MPNSTs or on the identification and classification of different MPNST types. Figure 5A shows a dimension reduction using Uni-form Mani-fold Approximation and Projection (UMAP) analysis plot. While all NF1-related MPNST cell lines were located within the MPNST cluster, the sporadic cell lines lay within the melanoma cluster (STS-26T and HS-Sch-2 cell lines) or within the not fully characterized group of MPNST-like sarcomas (HS-PSS cell line). With these results, we decided to further characterize the 8 cell lines by performing immunostaining for 3 markers: SOX9, SOX10, and S100B. SOX9 is normally expressed in MPNSTs^28^ and also in melanomas.^35^ SOX10 and S100B are markers that define the neural crest-Schwann cell differentiation axis but also the neural crest-melanocytic path. Both markers are frequently reduced or absent in MPNSTs according to the WHO classification,^36^ lost in transitions of ANNUBP toward a low-grade MPNST^12^ and also significantly downregulated/absent using expression analysis^28^; and data not shown). In contrast, SOX10 and S100B are frequently expressed in melanoma.^37^ All cell lines stained positive for SOX9 (Figure 5B). All NF1-related cell lines stained negative for SOX10 and S100B, as did the STS-26T sporadic cell line. In contrast, HS-PSS and HS-Sch-2 sporadic cell lines stained positive for SOX10 and, in addition, HS-Sch-2 also stained positive for S100B (Figure 5B), moving them away from a classic MPNST identity.

Altogether, the fine landscape of genomic alterations, methylome-based classification, marker expression, and particular informative gain-of-function mutations, captured a fair degree of variability among MPNST cell lines (Figure 5C) uncovering the probable misidentification of some of the tumors from which the sporadic cell lines were isolated and also the need of a complete and systematic characterization of additional MPNST tumors and cell lines to better understand whether MPNSTs constitute a homogeneous group of tumors or there exist different types.

To investigate the impact of genomic differences identified genuine MPNST cell lines and the ones potentially misidentified (Figure 5C), we performed a drug response assay. We used a MEK inhibitor targeting the loss of the *NF1* gene (Selumetinib) and a BET inhibitor targeting the inactivation of PRC2 (JQ1), since both TSGs had different inactivation patterns in the two groups of cell lines (Figure 4B). Both compounds tested showed a higher impact on cell viability in NF1-related cell lines, compared to the sporadic ones (Figure 5D), highlighting the importance of correct diagnostics of MPNSTs.

## Discussion

We performed a deep genomic characterization of 9 of the most distributed and used MPNST cell lines^25^ including cell lines banked in repositories (ATCC, RIKEN). Before this analysis, we performed authentication assays which resulted in discarding the T265 cell line since it exhibited the same STR profile as the ST88-14 and its matched primary MPNST (Data S2).

Despite the diverse ploidies exhibited by the MPNST cell lines, NF1-related cell lines faithfully reproduced the copy number profiles present in primary MPNST tumors, something that was not true for sporadic cell lines. These results reinforce the idea that despite the high degree of genomic alterations, MPNSTs contain quite stable genomes, as shown by comparing primary tumors with derived orthotopic PDX.^31^ Our use of WGS was crucial for more complete detection of genomic alterations present in MPNSTs, due to the significant number of structural variants present.^19^ This was especially important for detecting the inactivation of MPNST-related tumor suppressor genes (TSGs), particularly for *CDKN2A* and the PRC2-related genes (*SUZ12/EED*). The use of WGS also allowed the identification of fusion genes, such as EML4-ALK, generated by an inversion affecting both genes in the HS-PSS sporadic cell line. The presence of fusion genes is common in other types of soft tissue sarcomas that otherwise contain genomes with few genomic alterations. However, fusion genes are not common in the karyotypically complex MPNSTs.^38^ In concordance with this idea, and supporting a non-MPNST identity, in addition to the EML4-ALK fusion gene, the HS-PSS cell line contained a ploidy close to 2n, containing only a few copy number alterations and structural changes (Figures 1A and Figures 2 and 3, and Data S4).

In contrast to the importance of somatic copy number alterations and structural rearrangements, MPNST cell lines exhibited a modest frequency of mutations, with moderate functional impact, mainly involving the inactivation of *TP53*. Notably, the frequency and the type of mutation signatures exhibited provided an important differential indicator. While all cell lines exhibited a similar number of mutations and similar mutational signatures, the STS-26T cell line contained a much higher number of mutations and an important contribution from mutational signature 7, predominantly found in skin cancers.

Genomic alterations or mutations constituting a gain-of-function were not common in NF1-related MPNST cell lines. In fact, we identified only two cell lines (S462 and NF90-8) with a highly gained region in chromosome 4 containing the *PDGFRA* and *KIT* receptors, consistent with previous reports^39^^,^^40^ but not, for instance, kinase-activating mutations or translocations, like those involving NTRK genes.^41^ In contrast, we identified the *BRAF* V600E mutation in the STS-26T cell line and the already mentioned EML4-ALK fusion gene in the HS-PSS cell line.

Our deep genomic characterization (ploidy, copy number profile, structural variants, mutation frequencies, and signatures, presence of gain-of-function mutations, and altered MPNST-related genes) questioned the MPNST identity of the analyzed sporadic cell lines. Methylome-based classification^34^ and immunofluorescence of cell identity markers (SOX9, SOX10, S100B) complemented genomic analysis. STS-26T cell line contained a functional PRC2 complex and the *BRAF* V600E mutation. It also exhibited a much higher mutation frequency than the other cell lines and an important COSMIC signature 7, predominantly present in skin cancers as previously described by Hayward et al. (2017).^42^ Finally, a methylome classifier unequivocally classified it as a melanoma. Taking together our compiled evidence, in our opinion, the original diagnosis of a “malignant schwannoma”^43^ if made today would probably be “melanoma.” In this regard, it would be interesting to further analyze MPNSTs with *BRAF* V600E mutations described in the literature^44^^,^^45^^,^^46^ using additional tools like mutation frequencies and signatures, methylome classifier, and cell identity marker expression. The HS-Sch-2 cell line showed a WT status for PRC2 genes but harbored the complete inactivation of *NF1* and *CDKN2A*. It was classified as melanoma by the methylome classifier and expressed the markers SOX10 and S100B. The expression of these two markers is lost in transitions from atypical neurofibromas to MPNSTs and is commonly significantly reduced or absent in MPNSTs.^12^ The HS-Sch-2 cell line also stained negative for the melanoma marker Melan-A (data not shown). The combination of positive staining for SOX10 and S100B and negative staining for Melan-A is characteristic of desmoplastic melanoma,^47^ which also commonly exhibits the complete inactivation of *NF1* and *CDKN2A*,^48^^,^^49^ neurotropism and nerve infiltration,^50^^,^^51^ the latter described in the original publication of this cell line.^52^ Finally, HS-PSS also showed a WT status for PRC2 genes, was assigned to the provisional and not fully characterized “MPNST-like sarcoma” methylation group, was positive for SOX10, contained an almost unaltered genome proximal to 2n but harbored a translocation generating the fusion gene EML4-ALK. This fusion gene is associated with a type of sarcoma termed epithelioid inflammatory myofibroblastic sarcoma^53^^,^^54^ which also contain a spindle cell component, being the most probable identity of the tumor from which HS-PSS was derived.

Preliminary data on drug treatment using these cell lines showed a different impact on cell viability between genuine MPNST cell lines and the ones potentially misidentified, highlighting the importance of correct MPNST diagnostics for better clinical management.

Despite the potential misdiagnosis of the three sporadic cell lines studied here, it is important to remark that these results do not imply that all sporadic MPNSTs (and derived cell lines) are not genuine MPNSTs. In fact, deep genomic analysis of primary MPNSTs (Magallón-Lorenz et al. in preparation), confirmed the existence of sporadic MPNSTs exhibiting the same genomic characteristics as the NF1-related MPNST cell lines reported here. Conversely, our results neither discard the misidentification of MPNSTs in the NF1 setting.

In summary, the new genomic and epigenomic characterization of MPNST cell lines provided in this work uncovered the misidentification of the commonly used NF1-related T265 MPNST cell line and, in addition, compiled multiple pieces of evidence to question the identity of the three sporadic MPNST cell lines analyzed here, proposing alternative identities for all of them: a melanoma for STS-26T; a desmoplastic melanoma for the HS-Sch-2 cell line; and an epithelioid inflammatory myofibroblastic sarcoma for the HS-PSS. These results may imply the need of determining the impact of their use in previous and probably current works being performed, considering the new information provided. It also alerts us, as a scientific community, that we need to improve the characterization and control of the cell lines and tissues we use in our research. But above all, it provides an opportunity to look ahead and improve our understanding of what is an MPNST and which types might exist. In this regard, a systematic combination by different laboratories of histological characterization together with these new ways of analyzing genomes and epigenomes opens the door to revising the manner we perform differential diagnostics of MPNSTs and related tumors.

Our results, in addition to generating a valuable resource for the study of new therapeutic strategies for MPNSTs, uncover the need to systematically analyze MPNSTs, combining pathology with genomic and molecular techniques. Genomic analysis such as copy number profiles, structural variants, mutation frequencies and signatures, presence of gain-of-function mutations, and the inactivation of specific TSGs, together with methylome-based sarcoma classification and cell identity marker analysis, emerge as valuable tools for a better differential diagnosis and classification of MPNSTs.

### Limitations of the study

Different limitations of this study exist. The cell lines used here may not completely represent the repertoire of recurrent genomic alterations present in MPNSTs due to their type and limited number. In addition, the use of established MPNST cell lines makes difficult the availability of the original MPNST for comparison purposes or of a normal pair tissue for the identification of a reliable set of somatic variants. Finally, all sporadic cell lines used here may be misclassified, which urges for the deep genomic characterization of additional MPNST cell lines.

## Ethics declarations

This work has been approved by the Germans Trias i Pujol Hospital (HUGTiP) Ethics Committee.

## STAR★Methods

### Key resources table

**Table:** 

REAGENT or RESOURCE	SOURCE	IDENTIFIER
**Antibodies**		

Mouse IgG anti-Sox9	Abcam	Cat# ab76997 RRID:AB_2194156
Rabbit IgG anti-Sox10	Abcam	Cat# ab108408 RRID:AB_10859341
Rabbit IgG anti-S100B	DAKO	Cat# Z0311 RRID:AB_10013383
Goat anti-Mouse IgG (H + L) Alexa Fluor 488	Thermo Fisher Scientific	Cat# A-11001 RRID:AB_2534069
Goat anti-rabbit IgG (H + L) Alexa Fluor 568	Thermo Fisher Scientific	Cat# A-11011 RRID:AB_143157

**Chemicals, peptides, and recombinant proteins**		

DMEM	Biowest	Cat# L0106-500
FBS	Biowest	Cat# S181B-500
L-glutamine	Gibco	Cat# 25030024
Trypsin-EDTA 0.25%	Gibco	Cat# 25200–056
PBS	Biowest	Cat# L0615-500
DMSO	Sigma-Aldrich	Cat# 276855
JQ1	MedChemExpress	Cat# HY-13030
Selumetinib	Tocris	Cat# 6815
DAPI	Stemcell Technology	Cat# 75004
Paraformaldehyde	Santa Cruz Animal Health	Cat# sc-281692
Triton X-100	Sigma-Aldrich	Cat# X100
Vectashield	Vector Laboratories	Cat# H-1000-10

**Critical commercial assays**		

Maxwell 16 LEV simply RNA Purification Kit	Promega	Cat# AS1270
AmpFlSTR Identifiler Plus Amplification kit	Applied Biosystems	Cat# 4322288
Quant-iT™ PicoGreen® dsDNA Assay	Thermo Fisher Scientific	Cat# P7589
BigDye Terminator v.3.1 Sequencing Kit	Applied Biosystems	Cat# 4337455
MTT assay	Sigma-Aldrich	Cat# M2128-1G
Gentra Puregene Core Kit A	Qiagen	Cat# 153667

**Deposited data**		

WES and SNP-array data of 8 MPNST cell lines	Magallón-Lorenz et al. (2021)^19^	https://nf.synapse.org/ Synapse: syn22392179
WGS of 8 MPNST cell lines	This paper	https://nf.synapse.org/ Synapse: syn22392179

**Experimental models: Cell lines**		

Human: S462 cell	Frahm et al. (2014)^55^	RRID: CVCL_1Y70
Human: ST88-14 cells	Fletcher et al. (1991)^56^	RRID: CVCL_8916
Human: NF90-8 cells	Legius et al. (1994)^57^	RRID: CVCL_1B47
Human: sNF96.2 cells	Perrin et al. (2007)^30^ ATCC	CRL-2884; RRID: CVCL_K281
Human: NMS-2 cells	Imaizumi et al. (1998)^58^ RIKEN	RCB2347; RRID: CVCL_4662
Human: T265 cells	Badache et al. (1998)^59^	RRID: CVCL_S805
Human: STS-26T cells	Dahlberg et al. (1993)^43^	RRID: CVCL_8917
Human: HS-Sch-2 cells	Sonobe et al. (2000)^52^ RIKEN	RCB2230; RRID: CVCL_8718
Human: HS-PSS cells	RIKEN	RCB2362; RRID: CVCL_8717
Human: HFF-1	ATCC	SCRC-1041; RRID:CVCL_3285

**Oligonucleotides**		

Primers for interspecies PCR assay, see Supplementary Data S1, Table1	This paper	N/A
Primers for *CDKN2A* and *TP53* breakpoints, see Supplementary Data S6, Table1	This paper	N/A
Primers for *EML4-ALK* fusion gene	Takeuchi et al. (2008)^32^	https://doi.org/10.1158/1078-0432.CCR-08-1018

**Software and algorithms**		

CLC workbench 6 software	Qiagen	https://digitalinsights.qiagen.com/products-overview/discovery-insights-portfolio/analysis-and-visualization/qiagen-clc-main-workbench/
FlowJo	BD Bioscience	https://www.flowjo.com/
Integrative Genomic Viewer (IGV)	Robinson et al. (2011)^60^	https://software.broadinstitute.org/software/igv/
BWA-MEM	Li (2013)^61^	http://arxiv.org/abs/1303.3997
Strelka2	Kim et al. (2018)^62^	v2.9.10 https://github.com/Illumina/strelka/blob/v2.9.x/docs/userGuide/quickStart.md
annovar	Wang et al. (2014)^63^	v 20191024 https://annovar.openbioinformatics.org/en/latest/#annovar-documentation
Lumpy-smoove	Layer et al. (2014)^64^	Lumpy (v0.2.13)-smoove (v0.2.5) https://github.com/brentp/smoove
CNVkit	Talevich et al. (2016)^65^	v0.9.7 https://cnvkit.readthedocs.io/en/stable/
CliffHunteR	In-house software	https://github.com/TranslationalBioinformaticsIGTP/CliffHunteR
Circos	Connors et al. (2009)^66^	v0.69–8 http://circos.ca/
R	https://cran.r-project.org/	4.0.2
Bioconductor	https://bioconductor.org	3.11
CopyNumberPlots	https://doi.org/10.18129/B9.bioc.CopyNumberPlots	v1.4.0 https://bioconductor.org/packages/release/bioc/html/CopyNumberPlots.html
KaryoploteR	Gel and Serra (2017)^67^	v1.14.0 http://bioconductor.org/packages/release/bioc/html/karyoploteR.html
mutSignatures	Fantini et al. (2020)^68^	v2.1.1 https://cran.r-project.org/web/packages/mutSignatures/index.html
umap	R package	v0.2.7.0 https://cran.r-project.org/web/packages/umap/index.html

**Other**		

*H*.*sapiens* NCBI reference genome GRCh38 with no ALT sequences	NCBI	https://ftp.ncbi.nlm.nih.gov/genomes/all/GCA/000/001/405/GCA_000001405.15_GRCh38/seqs_for_alignment_pipelines.ucsc_ids/GCA_000001405.15_GRCh38_no_alt_analysis_set.fna.gz
COSMIC Mutation Data	The Catalog Of Somatic Mutations In Cancer (COSMIC)	COSMIC v92
ICGC Somatic Mutations	International Cancer Genome Consortium (ICGC)	ICGC Release 28
Structural Variants problematic regions	Chiang et al. (2015)^69^	https://github.com/hall-lab/speedseq/blob/master/annotations/exclude.cnvnator_100bp.GRCh38.20170403.bed
Database of Genomic Variants (DGV)	MacDonald et al. (2014)^70^	https://genome.ucsc.edu/cgi-bin/hgTables

### Resource availability

#### Lead contact

Further information and requests for resources and reagents should be directed to and will be fulfilled by the lead contact, Eduard Serra (eserra@igtp.cat) and Bernat Gel (bgel@igtp.cat).

#### Materials availability

HS-Sch-2, HS-PSS, NMS-2 and HFF cell lines are commercially available. The other MPNST cell lines used in this study are available from the lead contact upon request.

### Experimental model and subject details

#### MPNST and other established cell lines

In this study, we used a set of MPNST cell lines that contains some of the most frequently used MPNST cell lines together with a few which can be found in known repositories (ATCC, RIKEN). We studied six NF1-associated cell lines: S462 (RRID:CVCL_1Y70),^55^ ST88-14 (RRID:CVCL_8916),^56^ NF90-8 (RRID:CVCL_1B47),^57^ sNF96.2 (RRID:CVCL_K281),^30^ NMS-2 (RRID:CVCL_4662),^58^ and T265 (RRID:CVCL_S805),^59^ although the latter was discarded as it was found to be misidentified; and three sporadic lines: STS-26T (RRID:CVCL_8917),^43^ HS-Sch-2 (RRID:CVCL_8718)^52^ and HS-PSS (RRID:CVCL_8717). Table 1 summarizes clinical information about patients and tumors from whom these cell lines were established. Human foreskin fibroblast (HFF-1, ATCC: SCRC-1041) were used as control cells for ploidy analysis. All cell lines were cultured under standard conditions (37°C and 5% CO2) with High Glucose DMEM with sodium pyruvate (Biowest) supplemented with 10% FBS (Biowest) and 2 mM L-glutamine (Gibco). They were passaged and harvested using trypsin-EDTA (Gibco).

### Method details

#### DNA extraction

Total DNA was extracted from cell lines using the Gentra Puregene Kit (Qiagen). DNA was quantified with Nanodrop 1000 spectrophotometer (Thermo Scientific). For SNP array, whole exome and genome sequencing and methylome experiments, a fluorescence-based quantification of DNA was performed either by using the Quant-iT PicoGreen dsDNA Assay (Thermo Fisher Scientific) or a Qubit fluorometer (Life Technologies).

#### STR profiling

DNA fingerprinting of short tandem repeats (STRs) was conducted for all MPNST cell lines using the AmpFlSTR Identifiler Plus Amplification kit (Applied Biosystems) following the manufacturer’s instructions. This kit is based on the analysis of 16 microsatellites, including the nine STRs used by the ATCC.

#### Calculation of cell ploidy by flow cytometry

About 1–2x10^6^ cells from each cell line were trypsinized, washed with PBS, and fixed in ice-cold 70% ethanol for 2h at −20°C. Then, cells were washed with PBS and resuspended in a citrate-phosphate buffer for at least 30 min, up to 2h. Cells were then washed with PBS- 1% FBS and propidium iodide (PI) was added. Cells in PI solution were treated with DNAse-free RNAse A for 30–45 min at 37°C and were ready for flow cytometry analysis. All samples were analyzed on a FACSCanto II flow cytometer (BD Biosciences, San Jose CA) and a total of 10,000 single cells were analyzed for each sample. Aggregated cells were excluded by gating out on a biparametric plot with DNA content pulse area versus width. Data was analyzed using FlowJo software (BD Biosciences, San Jose, CA). HFF-1 were used as 2n control cells.

#### *NF1* mutational status

Sanger sequencing was used to confirm previously described *NF1* constitutional pathogenic variant of the cell lines S462,^55^ ST88-14,^71^ NF90-8,^72^ sNF96.2.^30^ In this project, we identified the constitutional *NF1* pathogenic variant of NMS-2 cell line, and the *NF1* pathogenic variants present in the HS-Sch-2 cell line by whole-exome sequencing (see below) which were also confirmed by Sanger sequencing. We used specific primers targeting the mutation region in each case and the BigDye Terminator v.3.1 Sequencing Kit (Applied Biosystems). Sequences were generated with the ABI Prism 3100 Genetic Analyzer (Applied Biosystems) and analyzed with CLC Main Workbench 6 software.

#### SNP-array analysis

SNP-array data from the different cell lines and tumors was obtained from Magallon-Lorenz et al. (2021).^19^ In short, the analysis was performed using Illumina BeadChips (Human660W-Quad, OmniExpress v1.0 and OmniExpress 1.2) at the IGTP High Content Genomics Core Facility. Raw data were processed with Illumina Genome Studio to extract B allele frequency (BAF) and log R ratio (LRR). We used GAP^73^ to perform copy-number calling.

#### Whole-exome sequencing (WES) and whole-genome sequencing (WGS)

WES from the 8 MPNST cell lines was also previously analyzed in Magallón-Lorenz et al. (2021).^19^ In short, the exome was captured using Agilent SureSelect Human All Exon V5 kit (Agilent, Santa Clara, CA, US) and sequenced in a HiSeq instrument (Illumina, San Diego, CA, US) at Centro Nacional de Analisis Genomicos (CNAG, Barcelona, Spain) to a median of 165.5 million 100 bp paired-end reads per sample. Sequencing reads were then mapped with BWA-MEM^61^ against GRCh38 genome.

The whole genome of two cell lines (ST88–14 and S462) had already been sequenced for Magallón Lorenz et al. (2021).^19^ The WGS of the other 6 cell lines were produced for this work at BGI (Shenzhen, China). In short, the 6 libraries were prepared following standard DNBseq protocols, sequenced in a BGISEQ-500 to a median of 881 million 150 bp paired-end reads per sample and mapped with bwa mem against the GRCh38 genome.

#### Selection of putatively pathogenic somatic variants using WES and WGS

Small nucleotide variants were called with strelka2^62^ and annotated with annovar.^63^ We filtered strelka2 results from WGS data to select potentially driver variants affecting protein function as follows: we selected exonic and splicing variants and removed all synonymous variants then, we filtered out variants with a population frequency (AF_popmax) higher than 1%, classified as benign in ClinVar,^74^ annotated as benign or likely benign in Inter-Var automated,^75^ present in 3 or more of the cell lines or classified as pathogenic in more than 5 out of 7 in-silico predictors (SIFT pred,^76^ PolyPhen2 HDIV pred,^77^ LRT pred, Mutation Taster pred,^78^ Mutation Assessor pred,^79^ FATHMM pred,^80^ CLNSIG^74^) Then, we filtered out those variants with a variant allele frequency (VAF) lower than 0.1 as these variants were deemed as unlikely to be present in the original malignant cell. In addition, we removed non-frameshift deletion or insertion variants present in dbSNP and variants in highly variable genes (*MUC3A*, *MUC5AC*, *OR52E5*, *OR52L1*, *SMPD1*, *PRAMEF* and *LILR*). Finally, we filtered out the variants present in dbSNP except for those included in COSMIC somatic mutations (https://ftp.ncbi.nlm.nih.gov/snp/others/rs_COSMIC.vcf.gz) or the International Cancer Genome Consortium (ICGC) (https://ftp.ncbi.nlm.nih.gov/snp/others/snp_icgc.vcf.gz) variant lists. WES data was processed using the same approach and used to validate the variants identified in WGS data.

#### Mutational signatures

Raw variants called by Strelka2 in WGS data were also the basis for the mutational signature analysis. Since normal pairs were not available, we applied a series of filters to approximate a somatic callset: we filtered out the variants with a population frequency (AF_popmax) higher than 1%, called in more than one cell line, with a variant allele frequency (VAF) lower than 0.1 and, variants in highly variable genes (*MUC3A*, *MUC5AC*, *OR52E5*, *OR52L1*, *SMPD1*, *PRAMEF* and *LILR*). We also filtered out the variants in dbSNP except for those present in COSMIC and ICGC. We used this call set enriched in somatic variants with the mutSignatures^68^ R package to estimate the contribution of each of the thirty COSMIC mutational signatures to the mutational profile of each cell line.

#### Copy number variants (CNVs) from WGS

We called copy-number alterations from WGS using CNVkit^65^ with the recommended settings for WGS data with no matched normal pair (flat reference, difficult region black-list (https://github.com/Boyle-Lab/Blacklist/blob/master/lists/hg38-blacklist.v2.bed.gz), -no-edge option and 1000 bp bins). To obtain the exact copy number profile of each sample we used the threshold method with sample-specific thresholds defined considering the ploidy of each cell line obtained by flow cytometry. Summarized and per-cell line copy number profiles were plotted using the CopyNumberPlots (10.18129/B9.bioc.CopyNumberPlots) and karyoploteR^67^ R packages.

#### Structural variants and detection of fusion genes

We used LUMPY^64^ via Smoove (https://github.com/brentp/smoove) as a structural variant (SV) caller with parameters for small cohorts and excluding the problematic regions defined in https://github.com/hall-lab/speedseq/blob/master/annotations/exclude.cnvnator_100bp.GRCh38.20170403.bed.^60^ We also used CliffHunteR (https://github.com/TranslationalBioinformaticsIGTP/CliffHunteR), an in-house developed sensitivity-oriented R package for breakpoint detection, and a thorough visual inspection using Integrative Genomic Viewer (IGV)^60^ to detect breakpoints affecting tumor suppressor genes associated with MPNSTs (*NF1*, *CDKN2A*, *SUZ12*, *EED*, *TP53*, *PTEN*, *RB1*). To discard germline structural variants, we filtered out SVs present in the Database of Genomic Variants (DGV)^70^ and the SVs with the same breakpoints in more than two MPNST cell lines. Inter-chromosomal and intra-chromosomal rearrangements were plotted using circos.^66^ We defined the genome region affected by an SV as 1 Mb upstream and downstream of its breakpoints. To investigate the presence of known fusion genes, we crossed the SV breakpoints detected by LUMPY and CliffHunteR with the fusion genes in COSMIC (https://cancer.sanger.ac.uk/census).

#### DNA methylation and Uni-form Mani-fold Approximation and Projection (UMAP) analysis

DNA methylation profiles were generated using the Infinium MethylationEPIC (850k) BeadChip array (Illumina, San Diego, USA) according to the manufacturer’s instructions. The data was processed as previously described.^34^ The two-dimensional UMAP^81^ embedding was created using the 20,000 most variable CpGs from the DNA methylation profiles of the cell lines and the reference cohorts of soft tissue tumors.^34^ The UMAP analysis was performed using the R package umap (version 0.2.7.0) with default parameters except for n_neighbors = 8.

#### Validation of inter-chromosomal rearrangements

Inter-chromosomal rearrangements detected by LUMPY or CliffHunteR affecting genes commonly altered in MPNST were validated by PCR and Sanger sequencing. PCR primers, annealing temperatures and amplicon lengths are summarized in Data S6.

#### Fusion gene validation

EML4-ALK v5a fusion gene breakpoints were detected by LUMPY in HS-PSS cell line. EML4-ALK fusion gene was validated by RT-PCR and Sanger sequencing. Total RNA from HS-PSS cell line was extracted using the 16 LEV simplyRNA Purification Kit (Promega) following the manufacturer’s instructions in the Maxwell 16 Instrument (Promega). RNA was quantified with a Nanodrop 1000 spectrophotometer (Thermo Scientific). RNA (0.5 μg) was reverse transcribed using the Superscript III reverse transcriptase enzyme (Life technologies) according to the manufacturer’s instructions. PCR primers, annealing temperatures, and amplicon lengths were previously described by Takeuchi et al. (2008).^32^

#### Immunocytochemical analysis

Cells were fixed in 4% paraformaldehyde (PFA) (Santa Cruz Animal Health) in PBS for 15 min at room temperature, permeabilized with 0.1% Triton X-100 in PBS for 10 min, blocked in 10% FBS in PBS for 15 min, and incubated with the primary antibodies, SOX10, SOX9 and S100B overnight at 4°C. Secondary antibodies were Alexa Fluor 488- and Alexa Fluor 568- (Thermo Fisher Scientific). Nuclei were stained with DAPI (Stem Cell Technologies, 1:1000). Slides were mounted with Vectashield (Vector laboratories), and coverslips were secured with polish nail.

#### Cell viability assay

Compounds (Selumetinib and JQ1) were prepared at 10 mmol/L in DMSO, and were diluted 5-fold from 40 μmol/L to 0.064 μmol/L, with DMSO. Cells were seeded in 96-well plates (Corning) at a density between 2.000 and 10.000 cells/well. 24 h later, drugs were added in three replicates. After 48 h of incubation with the drugs, cell viability was analyzed using MTT assay (Sigma-Aldrich), following manufacturer’s instructions. The percentage of cell viability was calculated by normalizing the values to DMSO control cells.

### Quantification and statistical analysis

Bioinformatic analysis is thoroughly described in the method details section, including the exact software and statistical methods used. The meaning of value of n, and/or dispersion and precision measure (SEM) can be found in the Figure 5 legends.

## Data Availability

• WGS data generated in this paper, SNP-array and WES data of the 8 different MPNST cell lines data previously generated in Magallón-Lorenz et al. (2021),^19^ are jointly deposited in a publicly accessible unified repository at Synapse (https://www.synapse.org/#!Synapse:syn22392179/wiki/605466) (https://doi.org/10.7303/syn22392179) and is part of the NF Data Portal (https://nf.synapse.org/). • The code used in this paper is available on GitHub (https://github.com/TranslationalBioinformaticsIGTP/MPNSTcellLines) and archived on Zenodo (https://doi.org/10.5281/zenodo.7524265). CliffHunteR, an in-house software, is available on GitHub (https://github.com/TranslationalBioinformaticsIGTP/CliffHunteR) and also archived at Zenodo (https://doi.org/10.5281/zenodo.7524539). • Any additional information required to analyze the data reported in this paper is available from the lead contact upon request.

## Supplemental information

Document S1. Table S1 and Data S1-S6

Table S2. Variants present in cell lines with a potential impact on protein function, related to Figure 4

Table S3. Status of commonly altered genes in MPNSTs, related to Figure 4
