# Supplementary material for: Deep genomic analysis of malignant peripheral nerve sheath tumor cell lines challenges current malignant peripheral nerve sheath tumor diagnosis
Source: iScience. 2023 Jan 31;26(2):106096. doi: 10.1016/j.isci.2023.106096 (PMC9929861; doi:10.1016/j.isci.2023.106096)
Supplement: Document S1. Table S1 and Data S1-S6 [file mmc1.pdf]

iScience, Volume *26*

Supplemental information

Deep genomic analysis of malignant peripheral nerve sheath tumor cell lines challenges current malignant peripheral nerve sheath tumor diagnosis

Miriam Magallón-Lorenz, Ernest Terribas, Sara Ortega-Bertran, Edgar Creus-Bachiller, Marco Fernández, Gerard Requena, Inma Rosas, Helena Mazuelas, Itziar Uriarte-Arrazola, Alex Negro, Tereza Lausová, Elisabeth Castellanos, Ignacio Blanco, George DeVries, Hiroyuki Kawashima, Eric Legius, Hilde Brems, Viktor Mautner, Lan Kluwe, Nancy Ratner, Margaret Wallace, Juana Fernández-Rodriguez, Conxi Lázaro, Jonathan A. Fletcher, David Reuss, Meritxell Carrió, Bernat Gel, and Eduard Serra

Supplementary Table1: Short Tandem Repeats (STR) profile, Related to Table 1

Microsatellite	Chr. location	S462	ST88-14	NF90-8	sNF96.2	NMS-2	STS26T	HS-Sch-2	HS-PSS
**D8S1179**	8	10, 12	14	10, 12	13	12, 13	13, 14	14	14, 15
**D21S11**	21q11.2-q21	29	29, 32.3	30	30	30	30, 31	29, 30	28, 32
**D7S820**	7q11.21-22	8, 10	8	11	10, 11	9, 12	8, 11	8, 11	10, 12
**CSF1PO**	5q33.3-34	12	9, 12	12	12	12	10, 13	11, 13	10, 12
**D3S1358**	3p	14, 17	15, 18	15	16	15, 16	14	18	16
**TH01**	11p15.5	7, 8	9	6	6	6	6, 9.3	6, 7	6, 9
**D13S317**	13q22-31	12	12	8	10	12	9, 10	11	8, 10
**D16S539**	16q24-qter	11, 13	13	9	11	9	12, 13	11, 12	10
**D2S1338**	2q35-37.1	23	17, 23	16, 24	17	18, 19	20	20	19
**D19S433**	19q12-13.1	14	13, 14	13, 16	13, 14	13.2, 14.2	14	14.2	13
**vWA**	12p12-pter	19	16	17	17, 19	14, 19	17	15, 16	14, 15
**TPOX**	2p23-2per	8	11, 12	11	11	8, 9	8	8	8
**D18S51**	18q21.3	16	12	14, 18	16	17	17, 18	13, 14	14, 15
**AMEL**	Xp22.1-22.3 Yp11.2	X	X, Y	X	X	X, Y	X	X	X, Y
**D5S818**	5q21-31	12	12, 13	11	11	12, 13	11, 12	13	9, 11
**FGA**	4q28	20	21	25	22	21, 22	22, 23	23, 26	22

Data S1: Interspecies PCR.

Related to Table 1

In order to find in our MPNST cell line set any possible cross-contamination of cell lines from the most used laboratory species mouse and rat we performed an interspecies PCR assay based on the amplification of the mitochondrial DNA gene cytochrome c oxidase subunit I (*COX1*) that discriminated among human, mouse and rat DNA. **Figure 1** shows the results of our interspecies PCR: no cross-contamination from either mouse or rat cell line was detected in our MPNST cell line set.

Specific primers for *COX1* gene for the three species were obtained from Parodi *et al*. [1]. Moreover, *COX1* gene sequences of human, mouse and rat were used for the design of a fourth primer pair to obtain a shared amplicon of this gene among the three species. **Table 1** shows a full list of all the primers used. Each interspecies PCR reaction included 100 ng of DNA, mixed with 5 uL of GoTaq® Flexi Buffer (Promega), 1.5 mM of $MgCl_2$ (Promega), 0.2 mM of each dNTP (vWR), 0.4 μM of each primer (Life technologies), and 0.75 U of GoTaq® G2 Flexi DNA Polymerase (Promega), in a total volume of 25 uL. PCR experiments were performed in a thermocycler (Applied Biosystems). Conditions for amplification were as follows: 95°C for 5 min; 30 cycles of 95°C for 30 s, 58°C for 30 s, and 72°C for 30 s; and a final step at 72°C for 5 min. A negative control without DNA template and three positive controls with DNA from human 293T cell line and rodent cell lines C2C12 (mouse) and PC12 (rat) were also included. Finally, 5 uL of each amplification product were run on a 1% agarose gel, stained with SybrSafe (Life technologies), visualized in a transilluminator, and photographed.

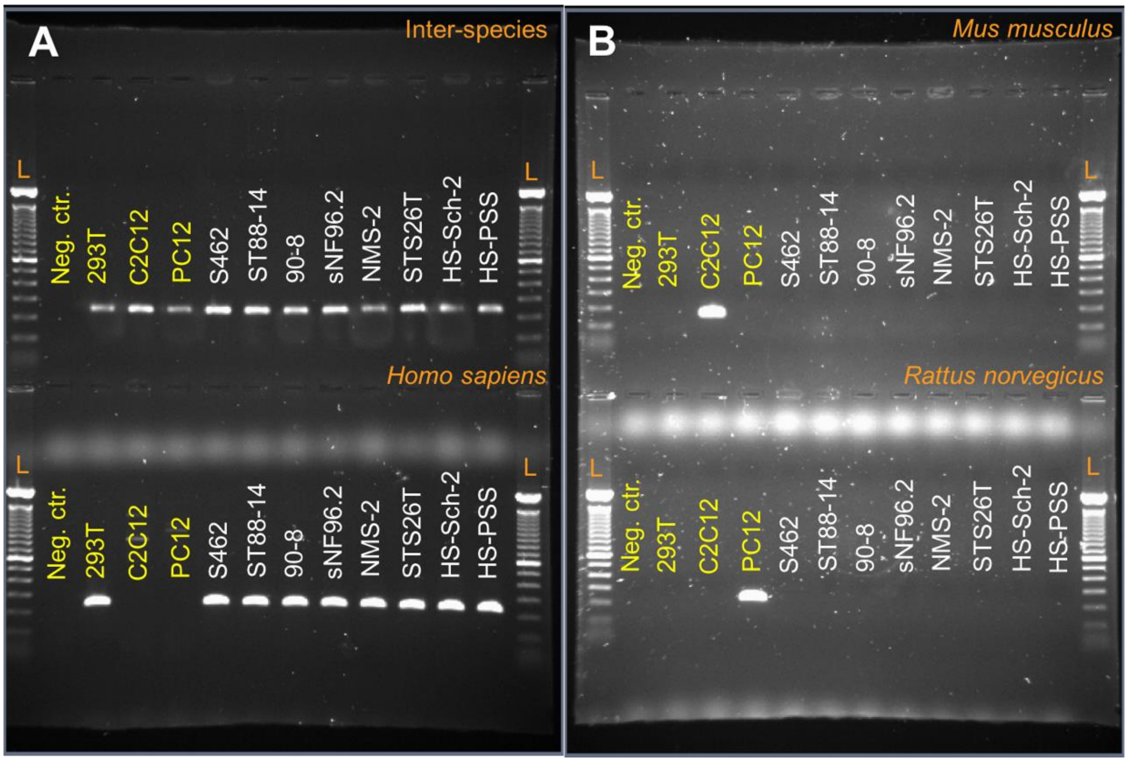

***Figure 1**. Agarose gel electrophoresis showing PCR amplicons from all four cytochrome c oxidase subunit I PCR experiments. A. Top: inter-species PCR showed 233-bp amplicons in all DNA samples. Bottom: Homo sapiens PCR showed 228-bp amplicons in human cell lines (293T and MPNST cell lines). B. Top: Mus musculus PCR showed a 150-bp amplicon only in the murine C2C12 cell line. Bottom: Rattus norvegicus PCR showed a 196-bp amplicon only in the PC12 cell line. Neg. ctr.: negative control; L: DNA ladder.*

Species	Primer name	Sequence 5'-3'	Amplicon length
Interspecies	IC-COX1-F	GATGCWTACACCACATGAAA	233 bp
	IC-COX1-R	GGGTTCGAWTCCTTCCTT	
Human	Hs-COX1-F	TTCGGCGCATGAGCTGGAGTCC	228 bp
	Hs-COX1-R	TATGCGGGGAAACGCCATATCG	
Mouse	Mm-Cox1-F	ATTACAGCCGTACTGCTCCTAT	150 bp
	Mm-Cox1-R	CCCAAAGAATCAGAACAGATGC	
Rat	Rn-Cox1-F	CGGCCACCCAGAAGTGTACATC	196 bp
	Rn-Cox1-R	GGCTCGGGTGTCTACATCTAGG	

***Table 1**. Primer sequences and amplicon sizes of interspecies, human, mouse and rat PCR experiments. W = A + T.*

References

1. Parodi B, Aresu O, Bini D, Lorenzini R, Schena F, Visconti P, et al. Species identification and confirmation of human and animal cell lines: A PCR-based method. Biotechniques. 2002;32:432–40.

Data S2:
Misidentification of the T265 MPNST cell line.

Related to Table 1

We obtained the MPNST cell lines used in this paper either from dedicated bioresources (RIKEN and ATCC) or from different collaborating laboratories. In some cases, the same cell line was sourced from distinct groups. That is the case of ST88-14, which was obtained from two labs. (**Figure 1**).

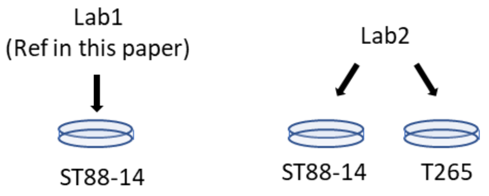

Figure 1*. MPNST cell lines were obtained from different labs.*

We first analyzed the small tandem repeat (STR) profile, to authenticate each cell line. The two cell lines from Lab2, ST88-14, and T265, showed the exact same STR profile. We compared them with the STR profile of the ST88-14 cell line we received from Lab 1, and they were all the same (**Table 1**). ST88-14 from Lab1 was finally used as the reference ST88-14 cell line (Ref) for this work.

Table 1. Small Tandem Repeat profile of ST88-14 and T265 cell lines

Microsatellite	Chr. location	ST88-14 (Ref)	ST88-14	T265
		Lab 1	Lab2	Lab2
D8S1179	8	14	14	14
D21S11	21q11.2-q21	29, 32.3	29, 32.3	29, 32.3
D7S820	7q11.21-22	8	8	8
CSF1PO	5q33.3-34	9, 12	9, 12	9, 12
D3S1358	3p	15, 18	15, 18	15, 18
TH01	11p15.5	9	9	9
D13S317	13q22-31	12	12	12
D16S539	16q24-qter	13	13	13
D2S1338	2q35-37.1	17, 23	17, 23	17, 23
D19S433	19q12-13.1	13, 14	13, 14	13, 14
vWA	12p12-pter	16	16	16
TPOX	2p23-2per	11, 12	11, 12	11, 12
D18S51	18q21.3	12	12	12
AMEL	Xp22.1-22.3 Yp11.2	X, Y	X, Y	X, Y
D5S818	5q21-31	12, 13	12, 13	12, 13
FGA	4q28	21	21	21

Both cell lines sharing the exact same STR profile pointed to a probable misidentification of one of the cell lines, so we decided to analyze either the original tumor from which the cell line was established, or the oldest possible cell culture passage preserved in the labs that generated these cell lines. In this regard, for the ST88-14 cell line [1] we were able to test DNA from the primary tumor (here labeled as ST88-14_PT), DNA from an early ST88-14 culture passage, passage 69 (ST88-14_CLp69) (Lab3). On the other hand, for the T265 cell line [2] we were able to test DNA from the oldest cryopreserved T265 cell line vial stored in Lab4 where the cell line was established (**Figure 2**). From all these samples we performed both an STR profile (**Table 2**) and an SNP-array analysis (**Figure 3**).

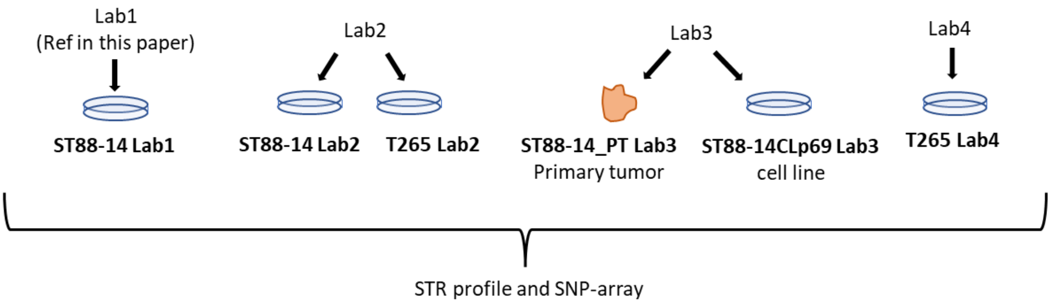

Figure 2. Scheme explaining the origin of the different samples and the analysis performed

*__Table 2__. STR profiles of all samples are represented in Figure 2, PT: primary tumor, CL: cell line; *: small peak.*

Microsatellite	Chr. location	ST88-14 Lab 1 (Ref)	ST88-14_PT Lab 3	ST88-14_CLp69 Lab 3	T265_Lab4 Lab4
D8S1179	8	14	14	14	14
D21S11	21q11.2-q21	29, 32.3	29, 32.3	29, 32.3	29, 32.3
D7S820	7q11.21-22	8	8	8	8
CSF1PO	5q33.3-34	9, 12	9, 12	9, 12	9, 12
D3S1358	3p	15, 18	15, 18*	15, 18	15, 18
TH01	11p15.5	9	9	9	9
D13S317	13q22-31	12	12	12	12
D16S539	16q24-qter	13	13	13	13
D2S1338	2q35-37.1	17, 23	17, 23	17, 23	17, 23
D19S433	19q12-13.1	13, 14	13, 14	13, 14	13, 14
vWA	12p12-pter	16	16	16	16
TPOX	2p23-2per	11, 12	11, 12	11, 12	11, 12
D18S51	18q21.3	12	12	12	12
AMEL	Xp22.1-22.3 Yp11.2	X, Y	X, Y	X, Y	X, Y
D5S818	5q21-31	12, 13	12, 13	12, 13	12, 13
FGA	4q28	21	21	21	21

The STR analysis **(Table 2)** demonstrated that all tested cell lines shared the same STR profile as the primary tumor from which the cell line ST88-14 was derived, and thus, both cell lines that arrived at our laboratory (ST88-14 and T265) were actually the same ST88-14 cell line. We cannot rule out the possibility that there exists a genuine T265 cell line in some laboratory. However, the oldest available vial of T265 shared the STR profile of ST88-14, suggesting that the misidentification happened early after the cell line establishment.

In addition to the STR profile, we performed an SNP-array analysis of all available samples (Figure 3 and Figure 4).

In **Figure 3** we can observe the SNP-array profile of the samples obtained from Lab 3 compared with the reference ST88-14 used in this paper from Lab 1. All samples share the same genomic profile, with only minor differences between them, consistent with the expected *in-vitro* evolution of the cell culture.

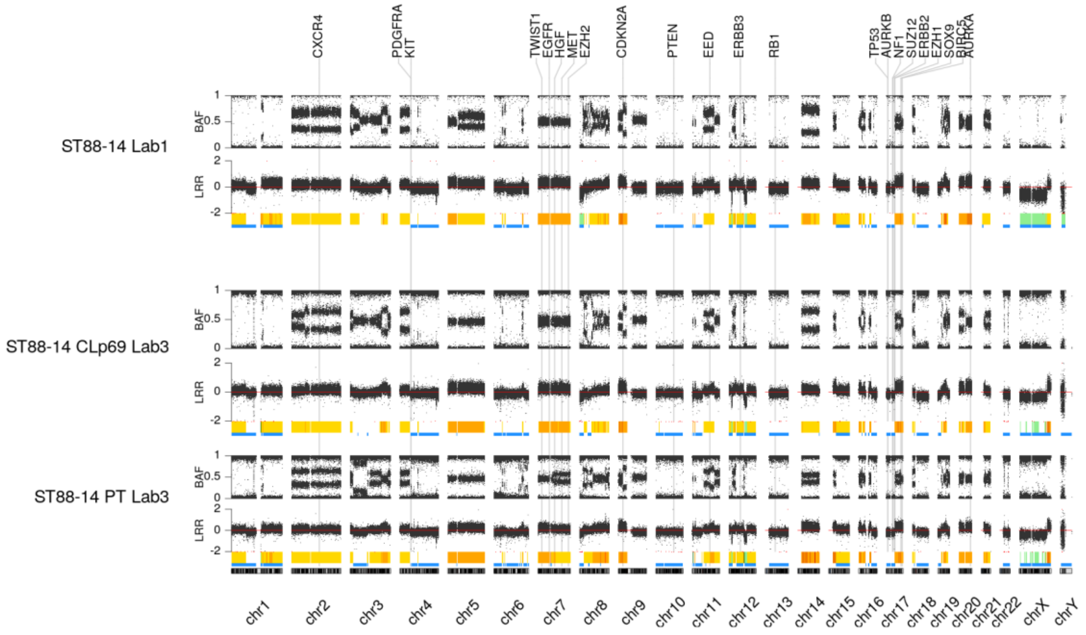

***Figure 3**. SNP-array profile of the samples we obtained from Lab 3 compared with the reference ST88-14 from Lab 1.*

Figure 4 represents the SNP-array profile of T265 cell lines we obtained from Lab 2 and from Lab 4 compared to our reference ST88-14 cell line. T265 samples again share the genomic profile with the reference ST88-14, although with a few additional differences when compared with ST88-14 samples in **Figure 3**. This could indicate an earlier separation from the original ST88-14 or be the result of culture bottlenecks. In any case, the genomic profiles of both T265 cell line samples show a common origin with the ST88-14 cell line.

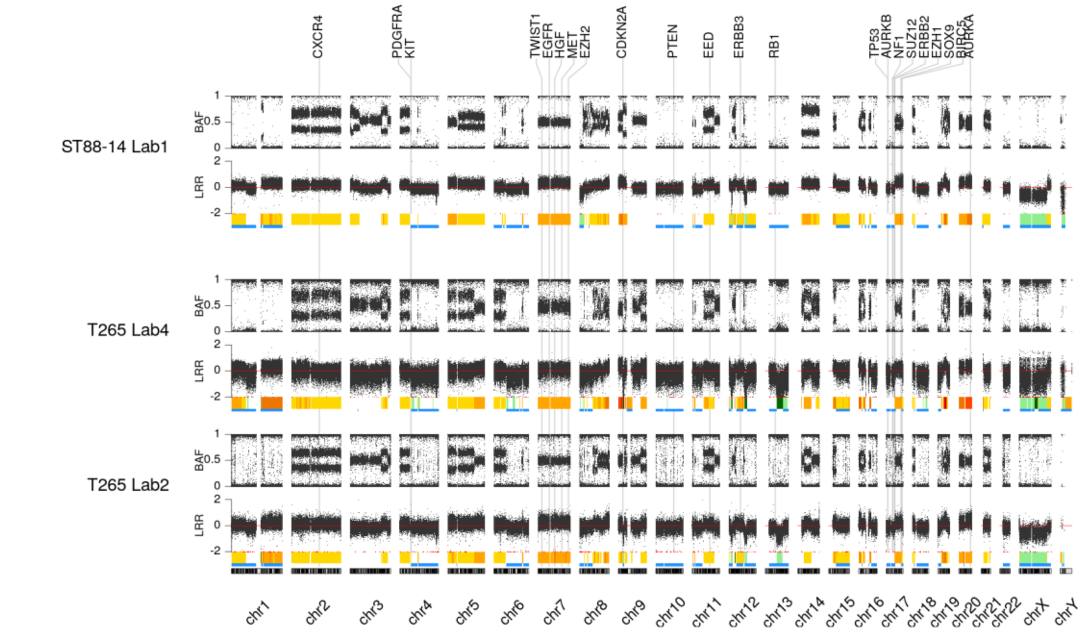

***Figure 4**. SNP-array profile of the T265 cell lines we obtained from Lab 2 and Lab 4compared with the reference ST88-14 from Lab 1.*

All in all, the STR profile and SNP-array analysis of current cell lines and the original primary tumor for ST88-14, confirm that the ST88-14 cell line originated from the expected primary tumor and that the current T265 cell line is, in reality, a misidentified ST88-14 cell line. In addition, the analysis of the oldest available T265 vial confirms that the misidentification happened very early after the establishment of the cell line. It is not clear if a real T265 cell line currently exists.

References

1. Fletcher JA, Kozakewich HP, Hoffer FA, Lage JM, Weidner N, Tepper R, et al. Diagnostic Relevance of Clonal Cytogenetic Aberrations in Malignant Soft-Tissue Tumors. New England Journal of Medicine. 1991;324:436–43.

2. Badache A, De Vries GH. Neurofibrosarcoma-derived Schwann cells overexpress platelet-derived growth factor (PDGF) receptors and are induced to proliferate by PDGF BB. Journal of Cellular Physiology. 1998;177:334–42.

Data S3: MPNST cell lines Data sheets

Related to Figure 1 , Figure 2, Figure 4 and Figure 5

S462

RRID:CVCL_1Y70

NF1

Female, 19yo

Phenotype

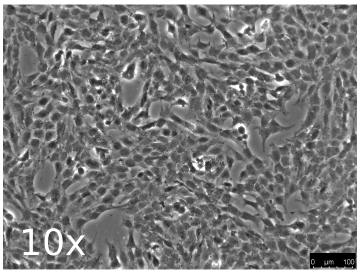

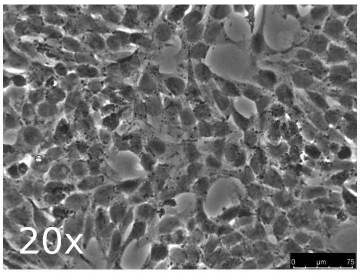

Ploidy

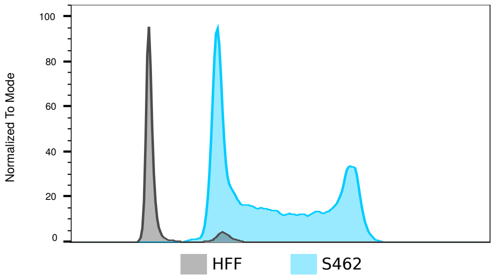

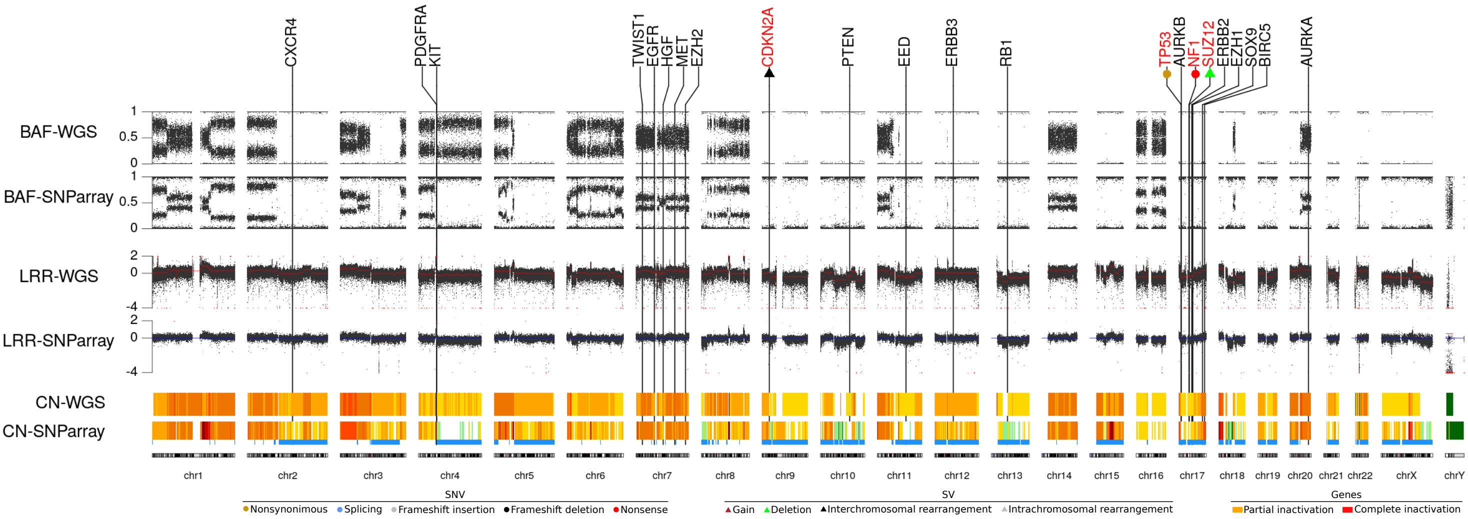

Genes

Tumor Supressor

- NF1 — c.6855C>A;LOH
- CDKN2A — Partial homozygous deletion
- SUZ12 — Heterozygous deletion;LOH
- EED
- TP53 — c.329G>C;LOH
- PTEN
- RB1

Membrane Receptors

- EGFR
- ERBB2
- ERBB3
- PDGFRA
- KIT
- MET
- HGF
- CXCR4

Transcription factors & other

- BIRC5
- TWIST1
- SOX9
- AURKA
- AURKB

WT · LOH · Homozygous loss · CN Gain · SV · SNV · Inactivation · Highly Gained

STR

D8S1179	10, 12
D21S11	29
D7S820	8, 10
CSF1PO	12
D3S1358	14, 17
TH01	7, 8
D13S317	12
D16S539	11, 13
D2S11338	23
D19S433	14
vWA	19
TPOX	8
D18S51	16
AMEL	X
D5S818	12
FGA	20

Methylome class

MPNST

Marker expression

SOX9: Positive SOX10: Negative S100B: Negative

Additional alterations:

Additional information:

Aliases:

Original publication: Silke Frahm, Victor-F Mautner, Hilde Brems, et. al., Genetic and phenotypic characterization of tumor cells derived from malignant peripheral nerve sheath tumors of neurofibromatosis type 1 patients, Neurobiology of Disease, Volume 16, Issue 1, Pages 85-91, 2004. https://doi.org/10.1016/j.nbd.2004.01.006

ST88-14

RRID:CVCL_8916

NF1

Male, 24yo

Phenotype

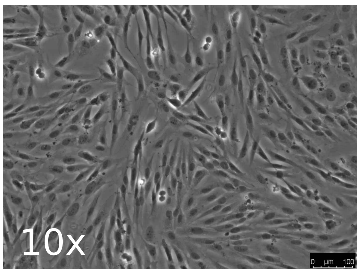

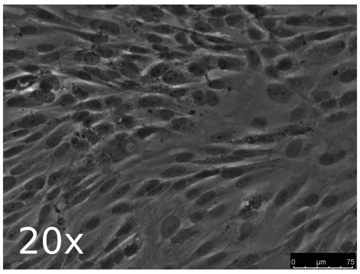

Ploidy

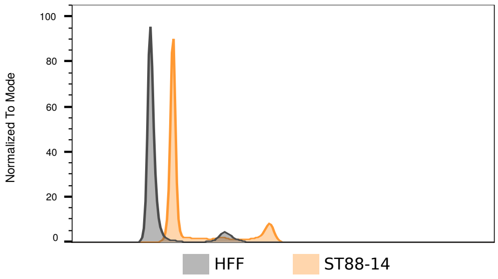

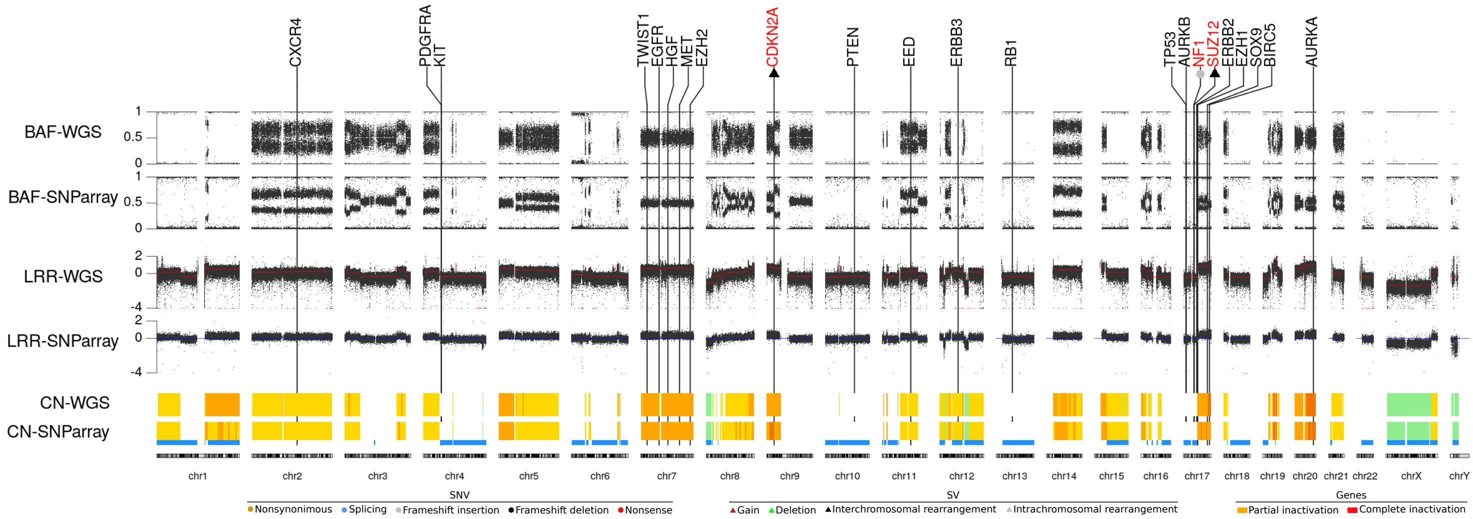

Genes

Tumor Supressor

Gene	Status
NF1	c.1649dupT;LOH
CDKN2A	Complete inactivation by translocations
SUZ12	Translocation;LOH
EED	
TP53	
PTEN	
RB1	

Membrane Receptors

EGFR
ERBB2
ERBB3
PDGFRA
KIT
MET
HGF
CXCR4

Transcription factors & other

BIRC5
TWIST1
SOX9
AURKA
AURKB

WT · LOH · Homozygous loss · CN Gain · SV · SNV · Inactivation · Highly Gained

STR

Marker	Alleles
D8S1179	14
D21S11	29, 32.3
D7S820	8
CSF1PO	9, 12
D3S1358	15, 18
TH01	9
D13S317	12
D16S539	13
D2S11338	17, 23
D19S433	13, 14
vWA	16
TPOX	11, 12
D18S51	12
AMEL	X, Y
D5S818	12, 13
FGA	21

Methylome class

MPNST

Marker expression

SOX9: Positive SOX10: Negative S100B: Negative

Additional alterations:

Additional information: In some repositories and journal articles the NF1 germline mutation has been erroneously identified as c.910C>T

Aliases: ST88.14; ST 88-14; ST-8814; ST8814; 88-14; NF188-14

Original publication: Fletcher JA, Kozakewich HP, Hoffer FA, Lage JM, Weidner N, Tepper R, Pinkus GS, Morton CC, Corson JM. Diagnostic relevance of clonal cytogenetic aberrations in malignant soft-tissue tumors. N Engl J Med. 1991 Feb 14;324(7):436-42. https://doi.org/10.1056/nejm199102143240702

NF90-8

RRID:CVCL_1B47

NF1

Female, 17yo

Phenotype

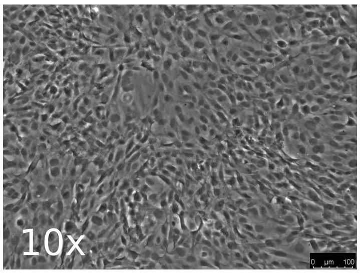

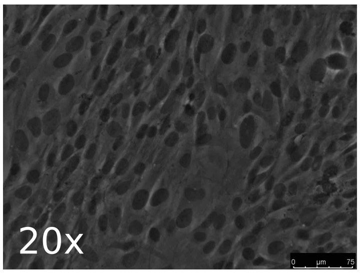

Ploidy

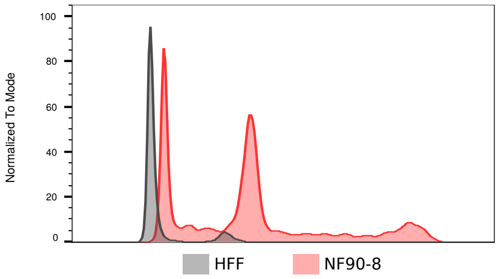

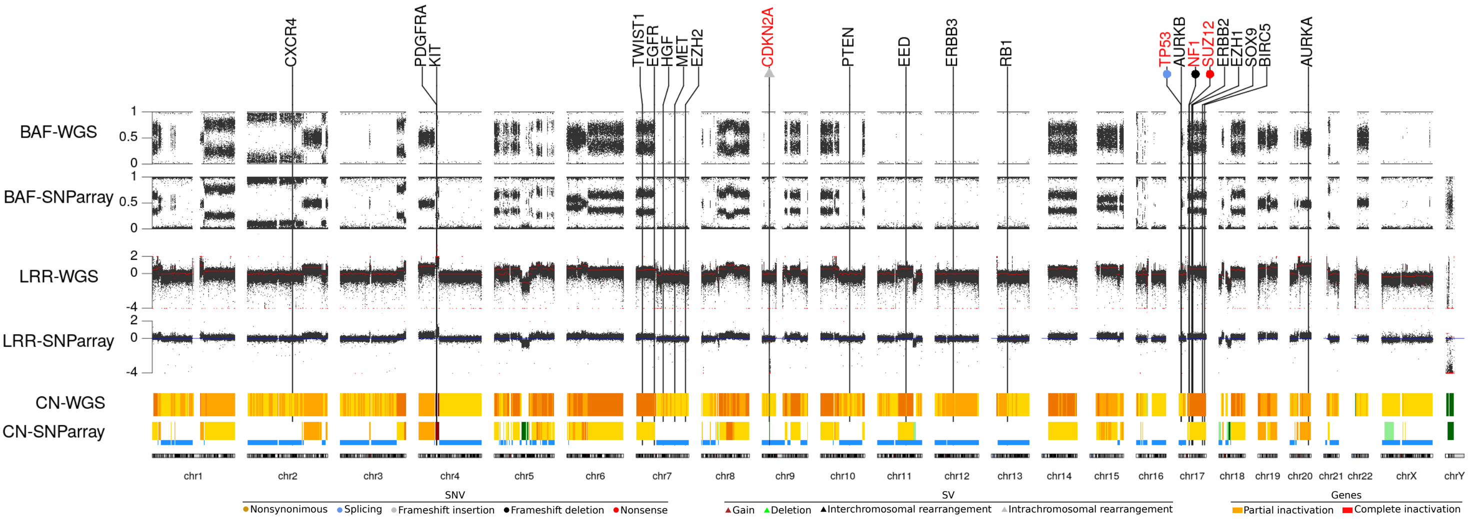

Genes

Tumor Supressor		Membrane Receptors	Transcription factors & other
NF1	c.3904_3910del;LOH	EGFR	BIRC5
CDKN2A	Homozygous deletion	ERBB2	TWIST1
SUZ12	c.T1290A;LOH	ERBB3	SOX9
EED		PDGFRA	AURKA
TP53	c.96+1G>A;LOH	KIT	AURKB
PTEN		MET	
RB1		HGF	
		CXCR4	

WT · LOH · Homozygous loss · CN Gain · SV · SNV · Inactivation · Highly Gained

STR

D8S1179	10, 12
D21S11	30
D7S820	11
CSF1PO	12
D3S1358	15
TH01	6
D13S317	8
D16S539	9
D2S11338	16, 24
D19S433	13, 16
vWA	17
TPOX	11
D18S51	14, 18
AMEL	X
D5S818	11
FGA	25

Methylome class

MPNST

Marker expression

SOX9: Positive SOX10: Negative S100B: Negative

Additional alterations:

Additional information:

Aliases: MPNST 90-8; 90-8TL; 90-8-TL; 90-8; NF190-8

Original publication: Legius E, Dierick H, Wu R, Hall BK, Marynen P, Cassiman JJ, Glover TW. TP53 mutations are frequent in malignant NF1 tumors. Genes Chromosomes Cancer. 1994 Aug;10(4):250-5. https://doi.org/10.1002/gcc.2870100405

sNF96.2

RRID:CVCL_K281

NF1

Male, 27yo

Phenotype

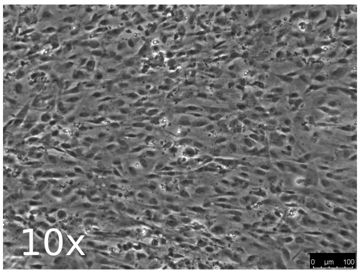

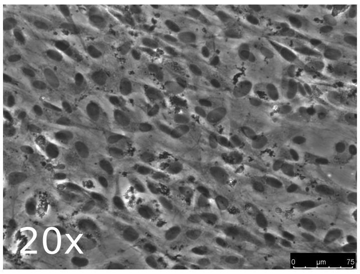

Ploidy

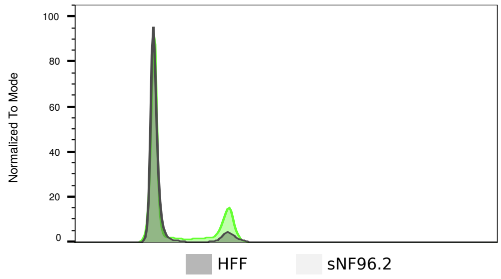

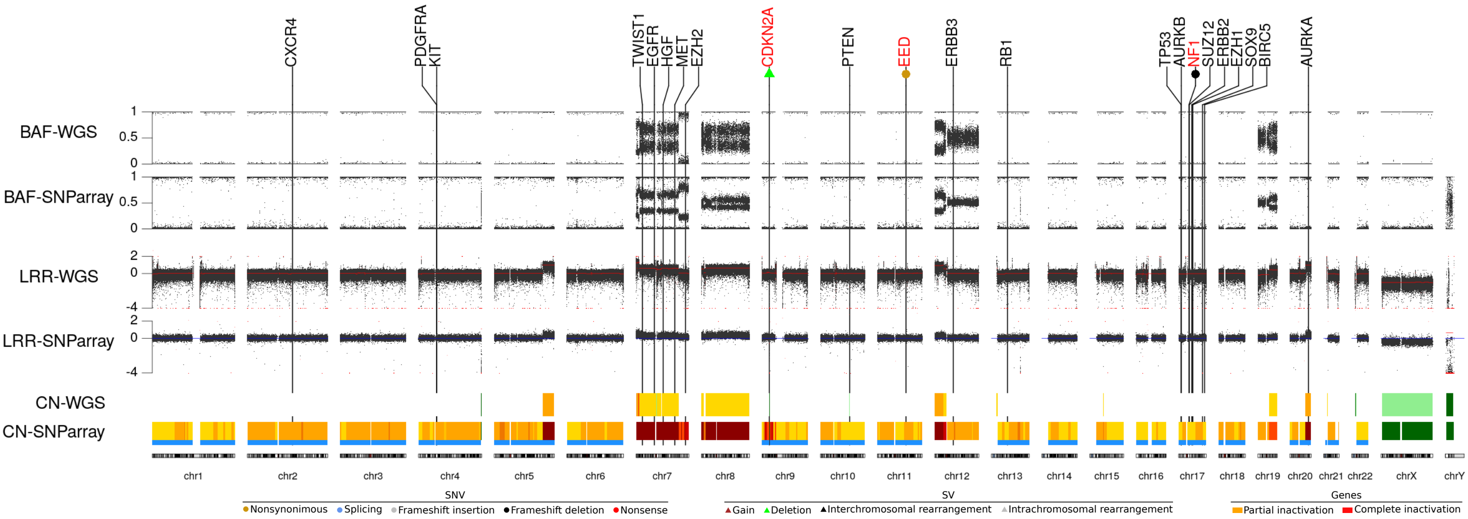

Genes

Tumor Supressor		Membrane Receptors	Transcription factors & other
NF1	c.3683delC;LOH	EGFR	BIRC5
CDKN2A	Homozygous deletion	ERBB2	TWIST1
SUZ12		ERBB3	SOX9
EED	c.721A>C;LOH	PDGFRA	AURKA
TP53		KIT	AURKB
PTEN		MET	
RB1		HGF	
		CXCR4	

WT, LOH, Homozygous loss, CN Gain, SV, SNV, Inactivation, Highly Gained

STR

D8S1179	13
D21S11	30
D7S820	10, 11
CSF1PO	12
D3S1358	16
TH01	6
D13S317	10
D16S539	11
D2S11338	17
D19S433	13, 14
vWA	17, 19
TPOX	11
D18S51	16
AMEL	X
D5S818	11
FGA	22

Methylome class

MPNST

Marker expression

SOX9: Positive SOX10: Negative S100B: Negative

Additional alterations:

Additional information:

Aliases: SNF96.2; sNF96-2

Original publication: Perrin GQ, Li H, Fishbein L, Thomson SA, Hwang MS, Scarborough MT, Yachnis AT, Wallace MR, Mareci TH, Muir D. An orthotopic xenograft model of intraneural NF1 MPNST suggests a potential association between steroid hormones and tumor cell proliferation. Lab Invest. 2007 Nov;87(11):1092-102. https://doi.org/10.1038/labinvest.3700675

NMS-2

RRID:CVCL_4662

NF1

Male, 30yo

Phenotype

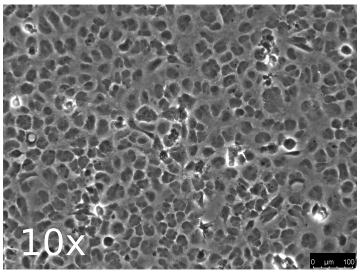

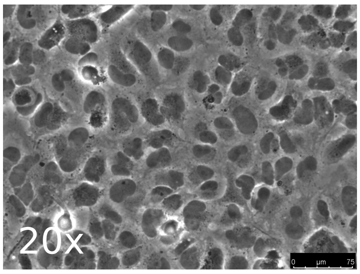

Ploidy

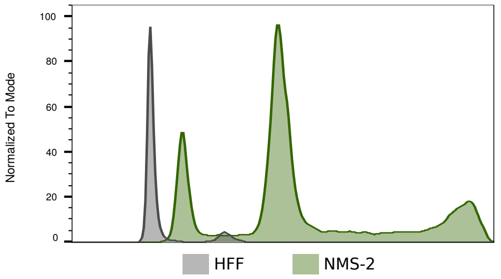

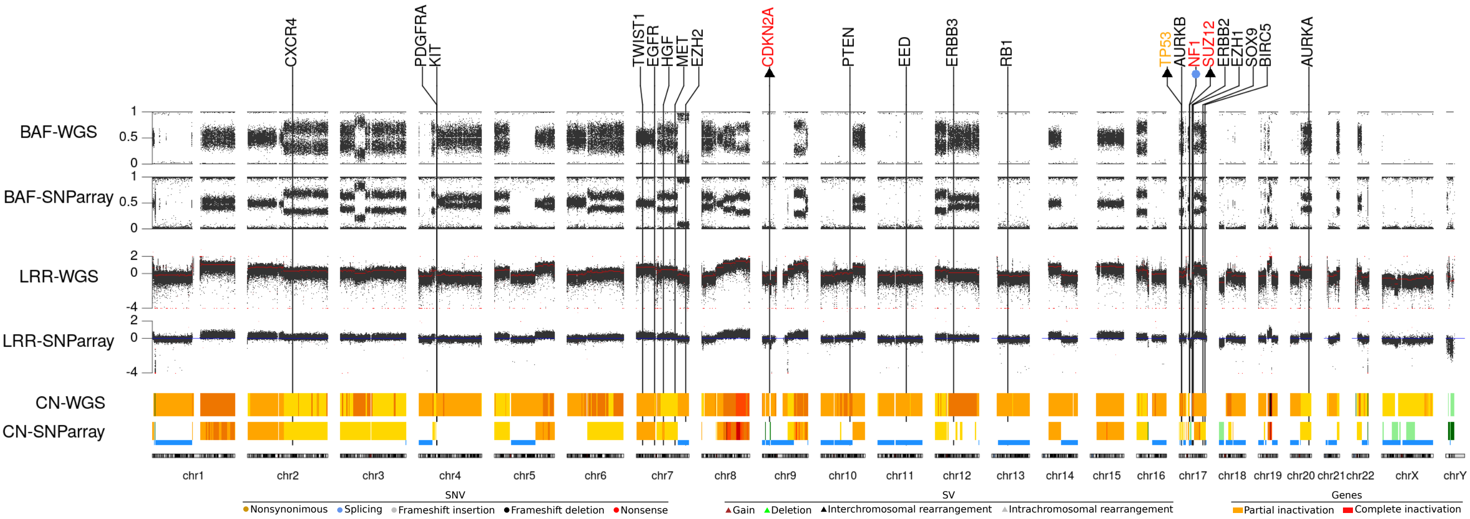

Genes

Tumor Supressor

- NF1 — c.7062+1G>T;LOH
- CDKN2A — Complete inactivation by translocations
- SUZ12 — Translocation;LOH
- EED
- TP53 — Translocation
- PTEN
- RB1

Membrane Receptors

- EGFR
- ERBB2
- ERBB3
- PDGFRA
- KIT
- MET
- HGF
- CXCR4

Transcription factors & other

- BIRC5
- TWIST1
- SOX9
- AURKA
- AURKB

Legend: WT, LOH, Homozygous loss, CN Gain, SV, SNV, Inactivation, Highly Gained

STR

D8S1179	12, 13
D21S11	30
D7S820	9, 12
CSF1PO	12
D3S1358	15, 16
TH01	6
D13S317	12
D16S539	9
D2S11338	18, 19
D19S433	13.2,14.2
vWA	14, 19
TPOX	8, 9
D18S51	17
AMEL	X, Y
D5S818	12, 13
FGA	21, 22

Methylome class

MPNST

Marker expression

SOX9: Positive   SOX10: Negative   S100B: Negative

Additional alterations:

Additional information:

Aliases:

Original publication: Imaizumi S, Motoyama T, Ogose A, Hotta T, Takahashi HE. Characterization and chemosensitivity of two human malignant peripheral nerve sheath tumour cell lines derived from a patient with neurofibromatosis type 1. Virchows Arch. 1998 Nov;433(5):435-41. https://doi.org/10.1007/s004280050271

STS-26T

RRID:CVCL_8917

Sporadic

Female, 51yo

Phenotype

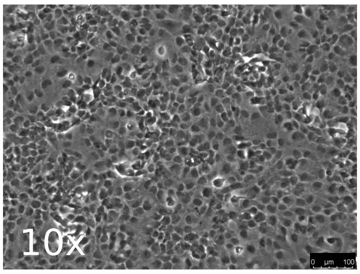

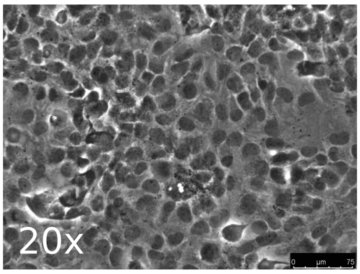

Ploidy

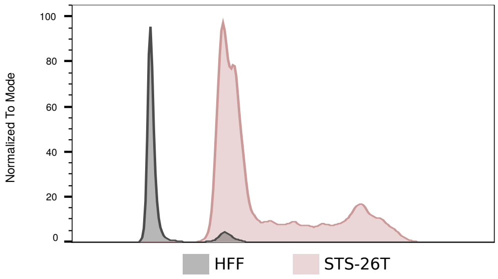

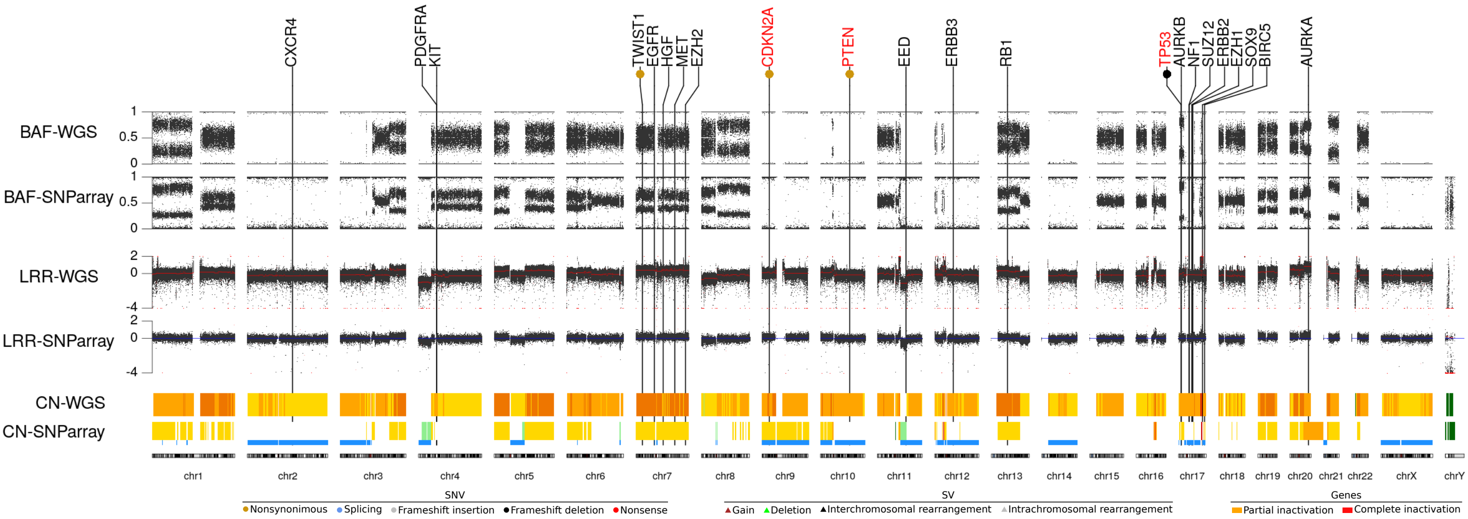

Genes

Tumor Supressor				Transcription factors & other	
NF1		EGFR		BIRC5	
CDKN2A	c.C341T;LOH	ERBB2		TWIST1	c.446T>C
SUZ12		ERBB3		SOX9	
EED		PDGFRA		AURKA	
TP53	c.232_241del;LOH	KIT		AURKB	
PTEN	c.1021TT>AA;LOH	MET			
RB1		HGF			
		CXCR4			

Legend: WT, LOH, Homozygous loss, CN Gain, SV, SNV, Inactivation, Highly Gained

STR

D8S1179	13, 14
D21S11	30, 31
D7S820	8, 11
CSF1PO	10, 13
D3S1358	14
TH01	6, 9.3
D13S317	9, 10
D16S539	12, 13
D2S11338	20
D19S433	14
vWA	17
TPOX	8
D18S51	17, 18
AMEL	X
D5S818	11, 12
FGA	22, 23

Methylome class

Melanoma

Marker expression

SOX9: Positive   SOX10: Negative   S100B: Negative

Additional alterations: BRAF:p.V600E

Additional information: High mutation frequency; Important contribution of COSMIC mutational signature 7 (predominantly seen in skin cancers)

Aliases: STS26T; STS26

Original publication: Dahlberg WK, Little JB, Fletcher JA, Suit HD, Okunieff P. Radiosensitivity in vitro of human soft tissue sarcoma cell lines and skin fibroblasts derived from the same patients. Int J Radiat Biol. 1993 Feb;63(2):191-8. https://doi.org/10.1080/09553009314550251.

HS-Sch-2

RRID:CVCL_8718

Sporadic

Female, 54yo

Phenotype

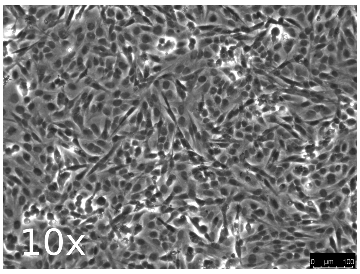

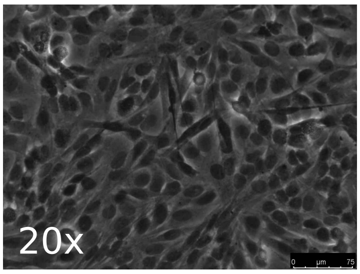

Ploidy

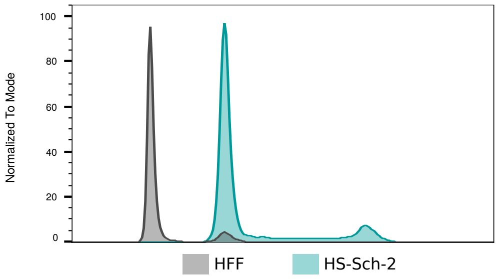

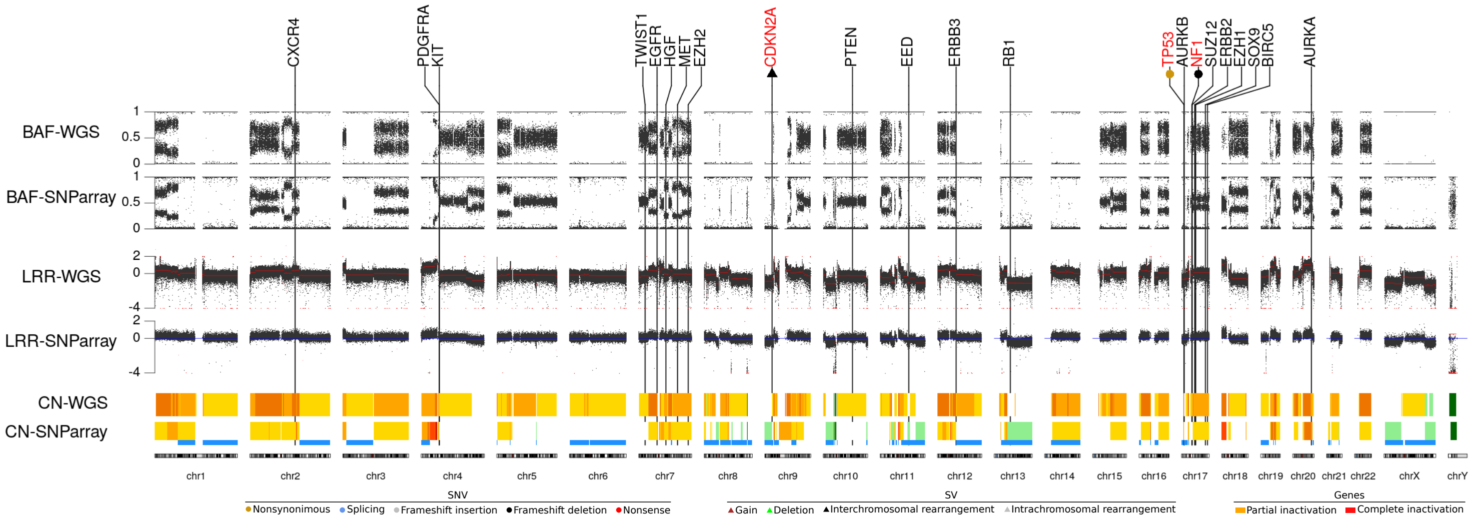

Genes

Tumor Supressor		Membrane Receptors	Transcription factors & other
NF1	c.270_288del;c.3113+1G>A	EGFR	BIRC5
CDKN2A	Translocation;LOH	ERBB2	TWIST1
SUZ12		ERBB3	SOX9
EED		PDGFRA	AURKA
TP53	c.818G>A;LOH	KIT	AURKB
PTEN		MET	
RB1		HGF	
		CXCR4	

WT LOH Homozygous loss CN Gain SV SNV Inactivation Highly Gained

STR

D8S1179	14
D21S11	29, 30
D7S820	8, 11
CSF1PO	11, 13
D3S1358	18
TH01	6, 7
D13S317	11
D16S539	11, 12
D2S11338	20
D19S433	14.2
vWA	15, 16
TPOX	8
D18S51	13, 14
AMEL	X
D5S818	13
FGA	23, 26

Methylome class

Melanoma

Marker expression

SOX9: Positive SOX10: Positive S100B: Positive

Additional alterations:

Additional information:

Aliases: HS-sch-2; HSSCH2

Original publication: Sonobe H, Takeuchi T, Furihata M, Taguchi T, Kawai A, Ohjimi Y, Iwasaki H, Kaneko Y, Ohtsuki Y. A new human malignant peripheral nerve sheath tumour-cell line, HS-sch-2, harbouring p53 point mutation. Int J Oncol. 2000 Aug; 17(2):347-52. https://doi.org/10.3892/ijo.17.2.347

HS-PSS

Sporadic

Male

RRID:CVCL_8717

Phenotype

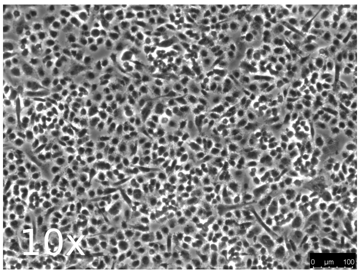

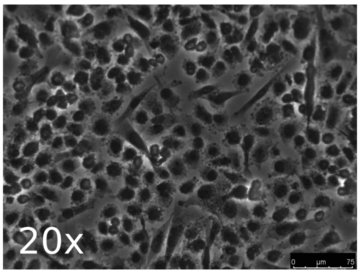

Ploidy

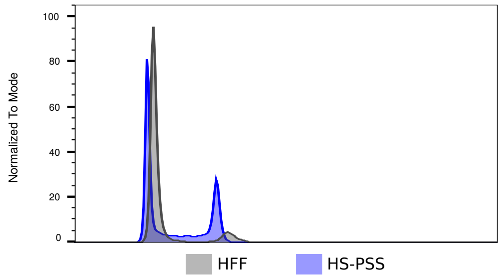

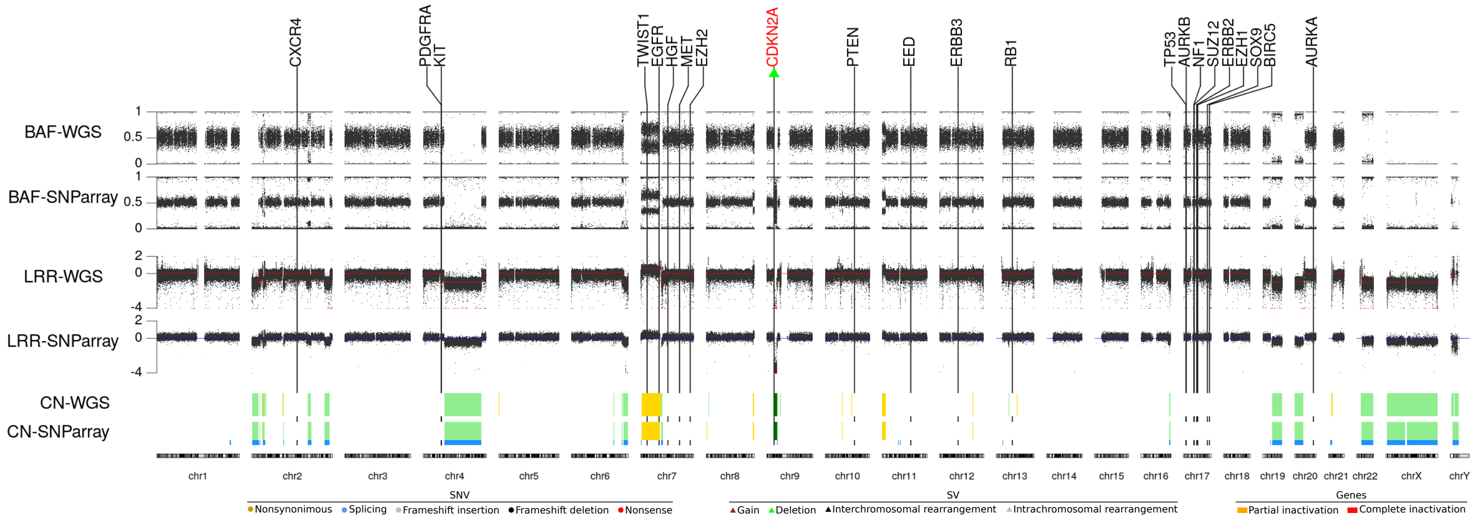

Genes

Tumor Supressor		Membrane Receptors	Transcription factors & other
NF1		EGFR	BIRC5
CDKN2A	Homozygous deletion	ERBB2	TWIST1
SUZ12		ERBB3	SOX9
EED		PDGFRA	AURKA
TP53		KIT	AURKB
PTEN		MET	
RB1		HGF	
		CXCR4	

WT LOH Homozygous loss CN Gain

SV SNV Inactivation Highly Gained

STR

D8S1179	14, 15
D21S11	28, 32
D7S820	10, 12
CSF1PO	10, 12
D3S1358	16
TH01	6, 9
D13S317	8, 10
D16S539	10
D2S11338	19
D19S433	13
vWA	14, 15
TPOX	8
D18S51	14, 15
AMEL	X, Y
D5S818	9, 11
FGA	22

Methylome class

MPNST-like

Marker expression

SOX9: Positive SOX10: Positive S100B: Negative

Additional alterations: Intrachromosomal rearrangement in chromosome 2 creating an EML4-ALK fusion gene

Additional information:

Aliases: Hs-PSS; HSPSS

Original publication: .

Data S4: Copy number profile for each MPNST cell line, chromosome by chromosome.

Related to Figure 2

S462

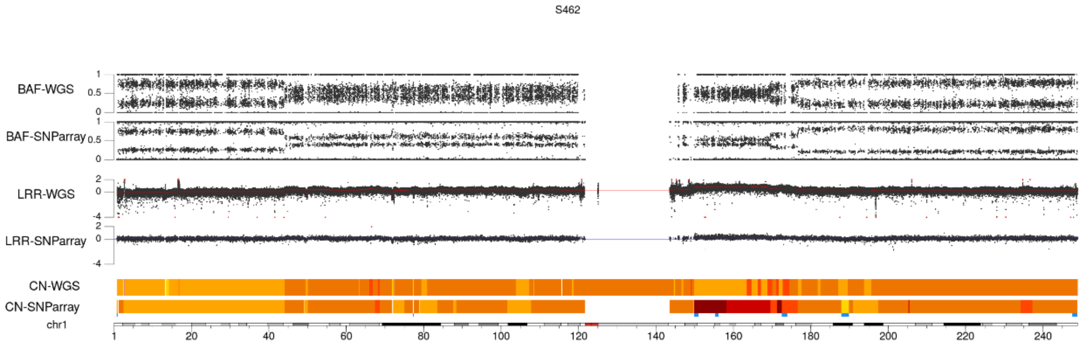

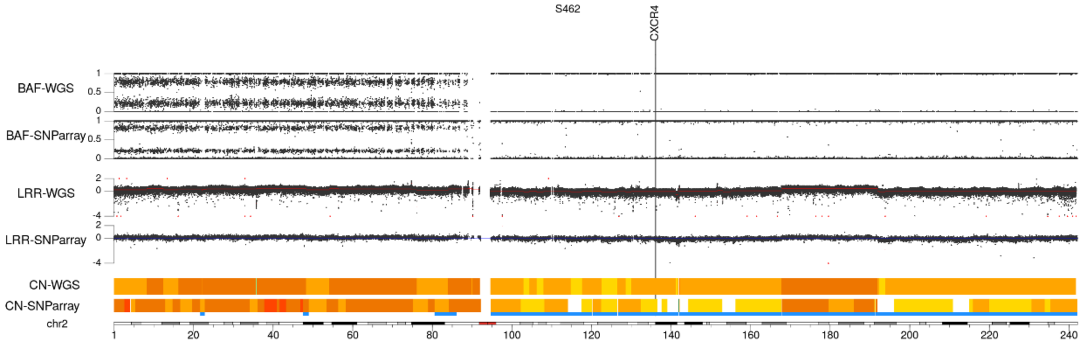

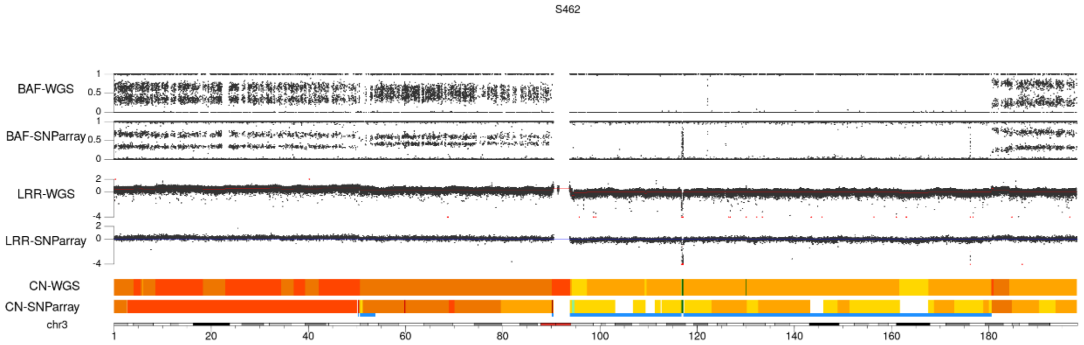

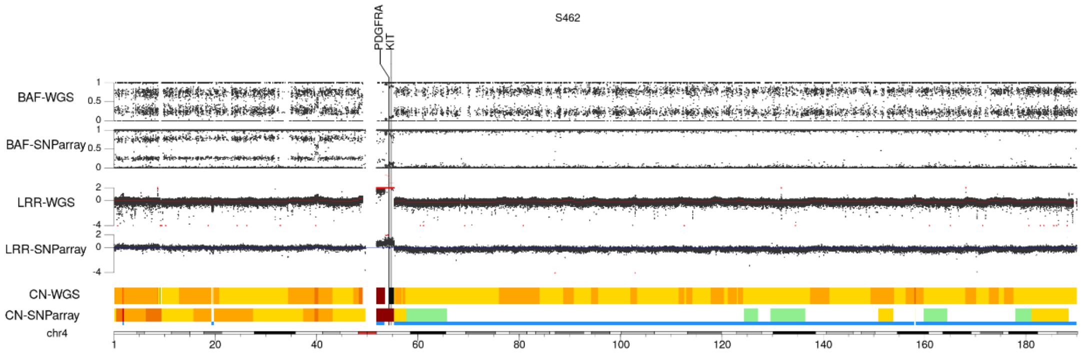
S462
PDGFRA
KIT
BAF-WGS
BAF-SNParray
LRR-WGS
LRR-SNParray
CN-WGS
CN-SNParray
chr4
1 20 40 60 80 100 120 140 160 180

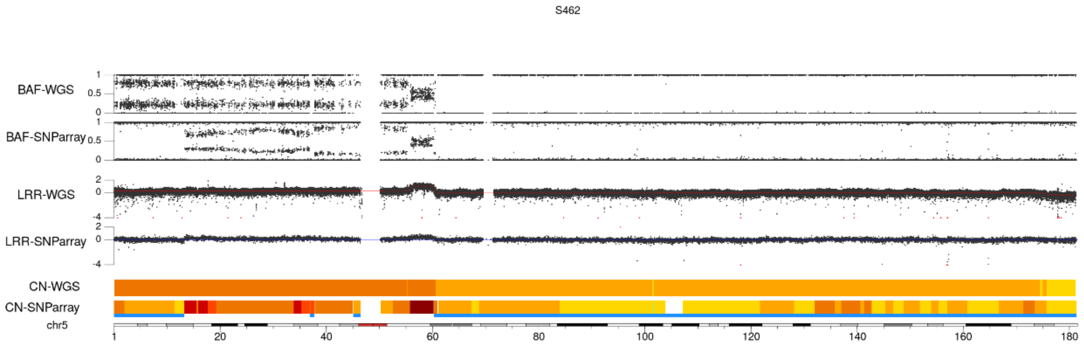
S462
BAF-WGS
BAF-SNParray
LRR-WGS
LRR-SNParray
CN-WGS
CN-SNParray
chr5
1 20 40 60 80 100 120 140 160 180

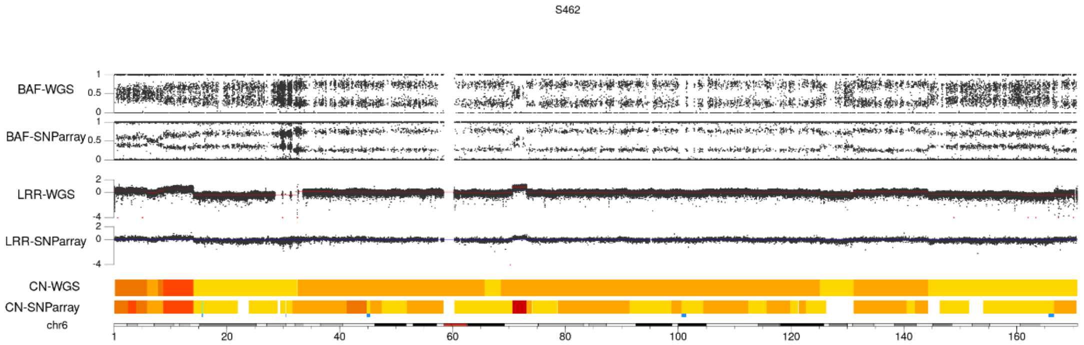
S462
BAF-WGS
BAF-SNParray
LRR-WGS
LRR-SNParray
CN-WGS
CN-SNParray
chr6
1 20 40 60 80 100 120 140 160

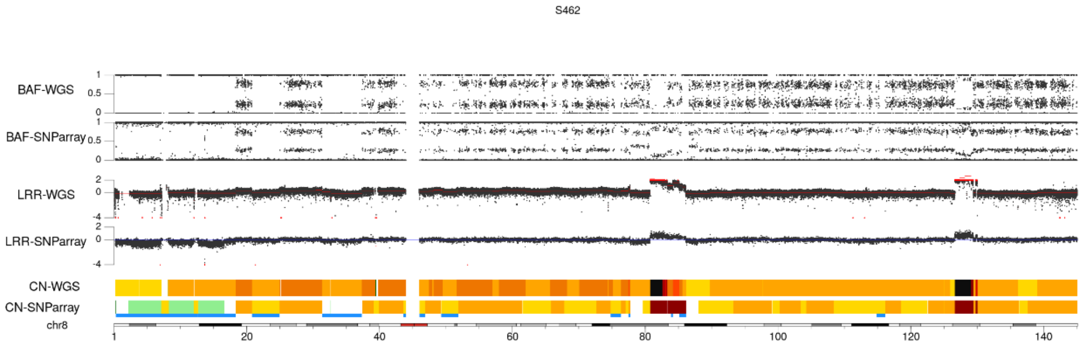
S462
BAF-WGS
BAF-SNParray
LRR-WGS
LRR-SNParray
CN-WGS
CN-SNParray
chr8
1 20 40 60 80 100 120 140

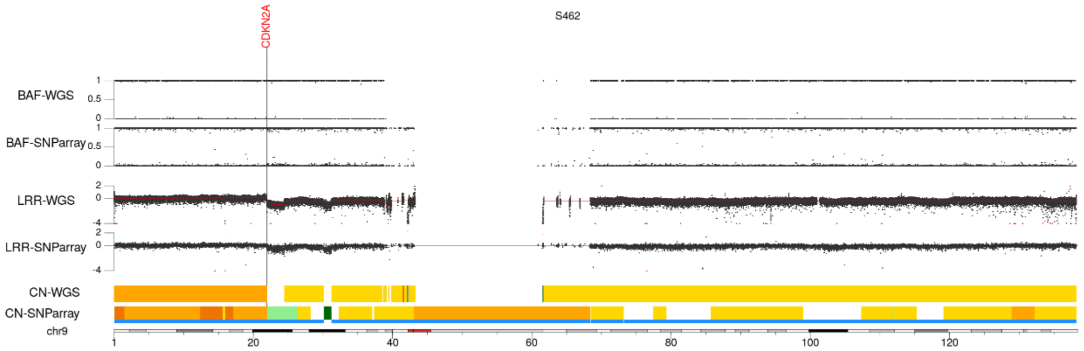
S462
CDKN2A
BAF-WGS
BAF-SNParray
LRR-WGS
LRR-SNParray
CN-WGS
CN-SNParray
chr9
1 20 40 60 80 100 120

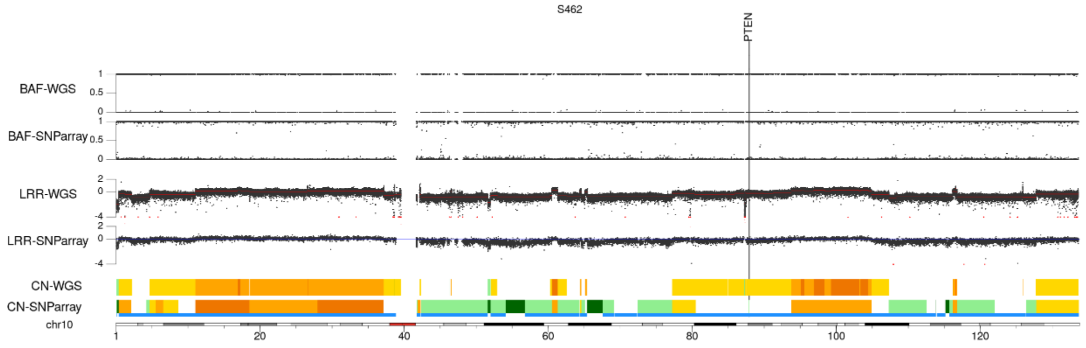
S462
PTEN
BAF-WGS
BAF-SNParray
LRR-WGS
LRR-SNParray
CN-WGS
CN-SNParray
chr10
1 20 40 60 80 100 120

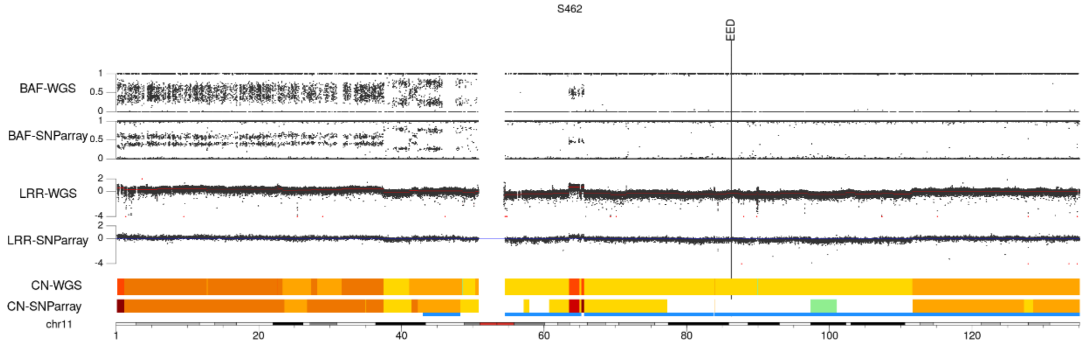
S462
EED
BAF-WGS
BAF-SNParray
LRR-WGS
LRR-SNParray
CN-WGS
CN-SNParray
chr11
1 20 40 60 80 100 120

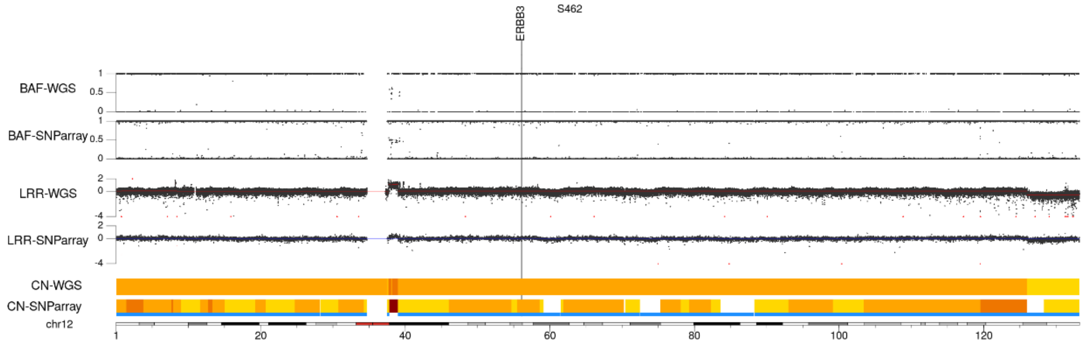
S462
ERBB3
BAF-WGS
BAF-SNParray
LRR-WGS
LRR-SNParray
CN-WGS
CN-SNParray
chr12
1 20 40 60 80 100 120

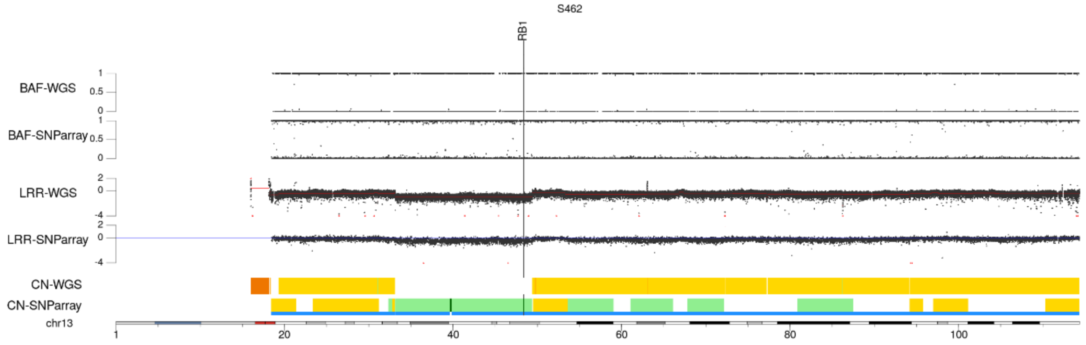
S462
RB1
BAF-WGS
BAF-SNParray
LRR-WGS
LRR-SNParray
CN-WGS
CN-SNParray
chr13
1 20 40 60 80 100

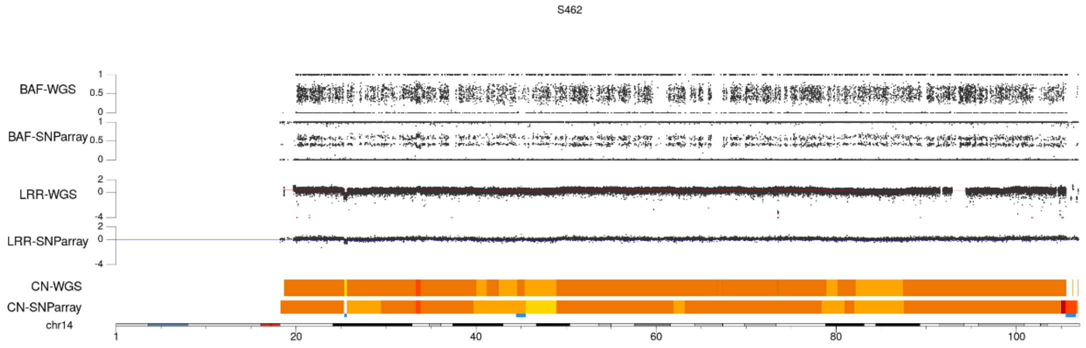
S462
BAF-WGS
BAF-SNParray
LRR-WGS
LRR-SNParray
CN-WGS
CN-SNParray
chr14
1 20 40 60 80 100

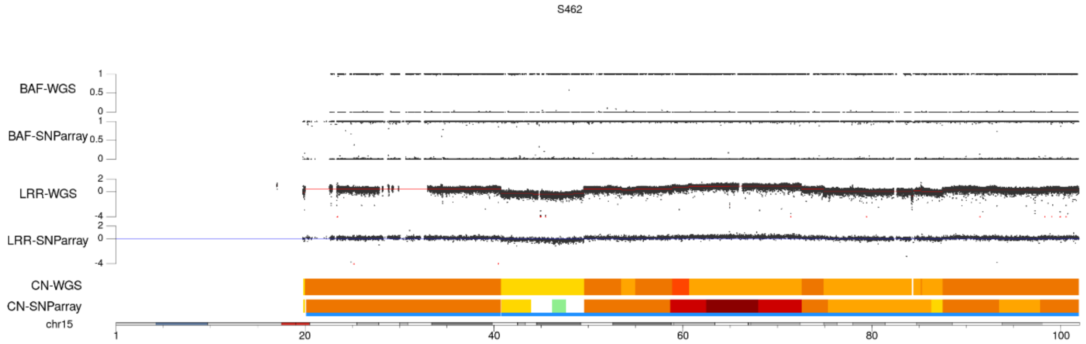
S462
BAF-WGS
BAF-SNParray
LRR-WGS
LRR-SNParray
CN-WGS
CN-SNParray
chr15
1
20
40
60
80
100

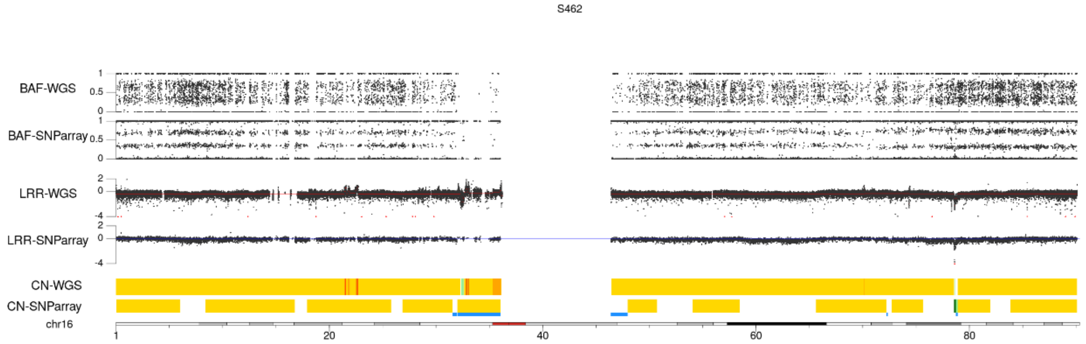
S462
BAF-WGS
BAF-SNParray
LRR-WGS
LRR-SNParray
CN-WGS
CN-SNParray
chr16
1
20
40
60
80

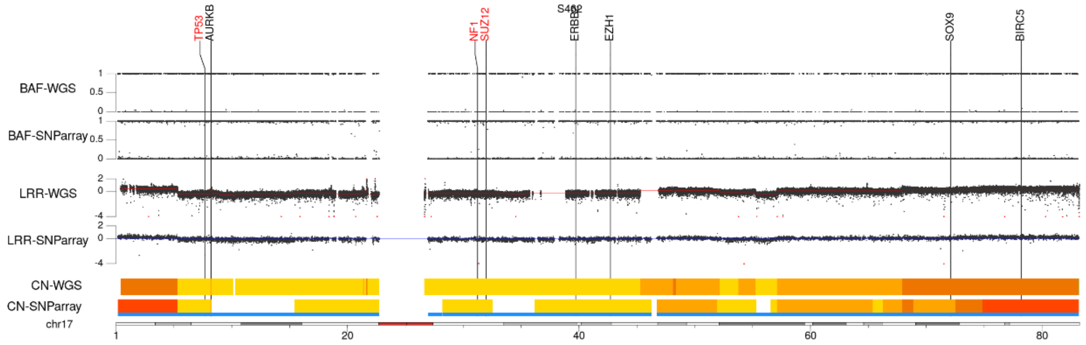
TP53
AURKB
NF1
SUZ12
S462
ERBB2
EZH1
SOX9
BIRC5
BAF-WGS
BAF-SNParray
LRR-WGS
LRR-SNParray
CN-WGS
CN-SNParray
chr17
1
20
40
60
80

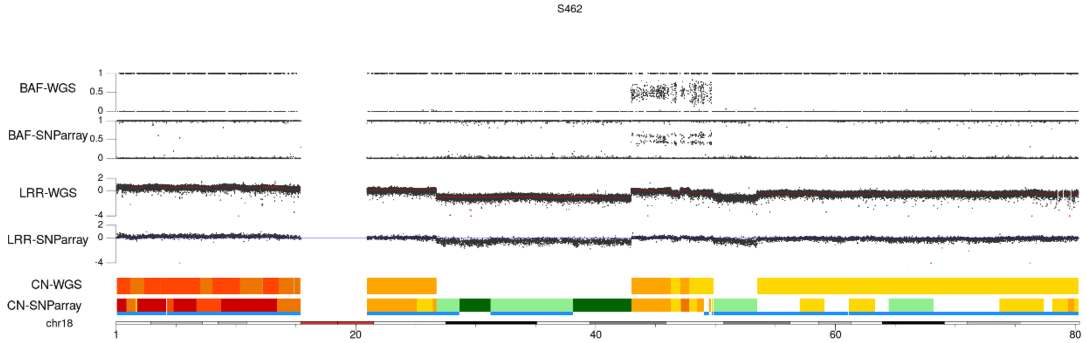
S462
BAF-WGS
BAF-SNParray
LRR-WGS
LRR-SNParray
CN-WGS
CN-SNParray
chr18
1
20
40
60
80

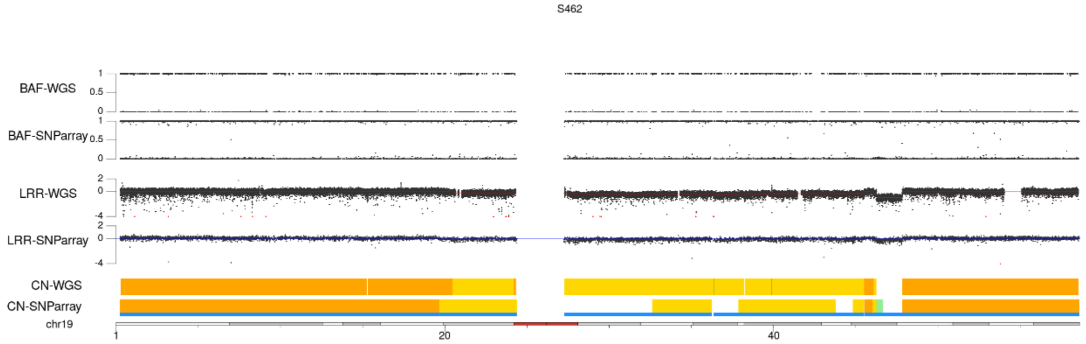
S462
BAF-WGS
BAF-SNParray
LRR-WGS
LRR-SNParray
CN-WGS
CN-SNParray
chr19
1
20
40

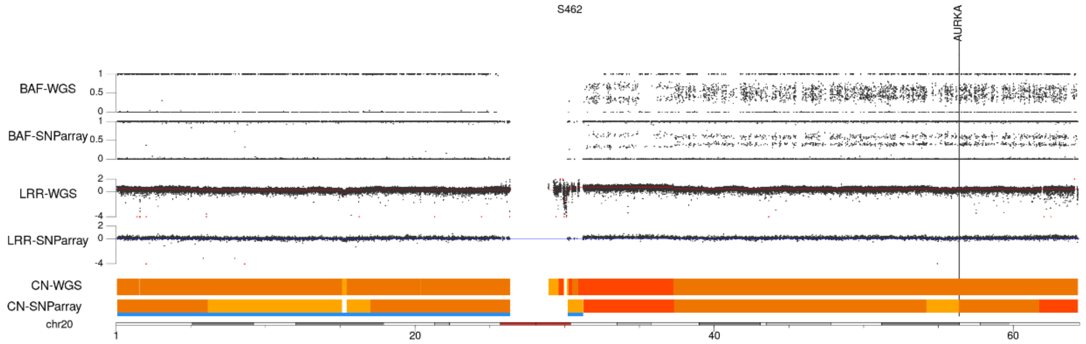
S462
AURKA
BAF-WGS
BAF-SNParray
LRR-WGS
LRR-SNParray
CN-WGS
CN-SNParray
chr20
1
20
40
60

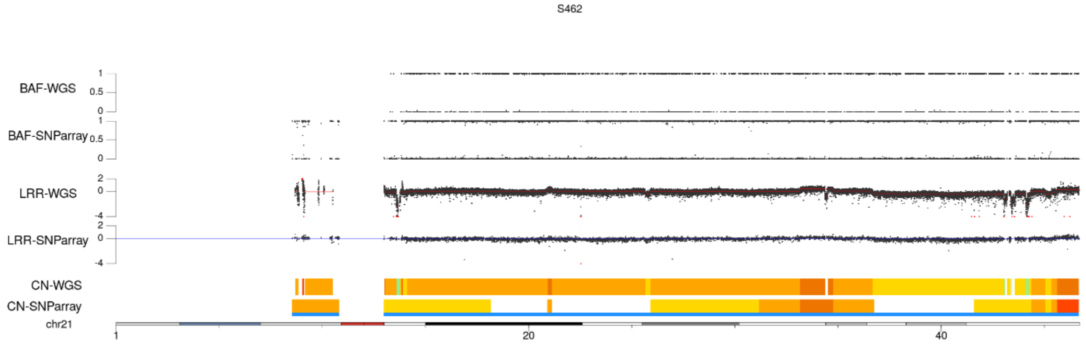
S462
BAF-WGS
BAF-SNParray
LRR-WGS
LRR-SNParray
CN-WGS
CN-SNParray
chr21
1
20
40

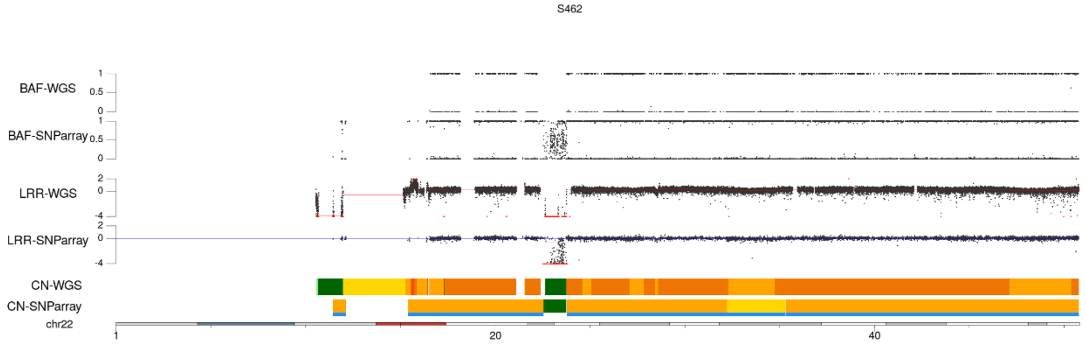
S462
BAF-WGS
BAF-SNParray
LRR-WGS
LRR-SNParray
CN-WGS
CN-SNParray
chr22
1
20
40

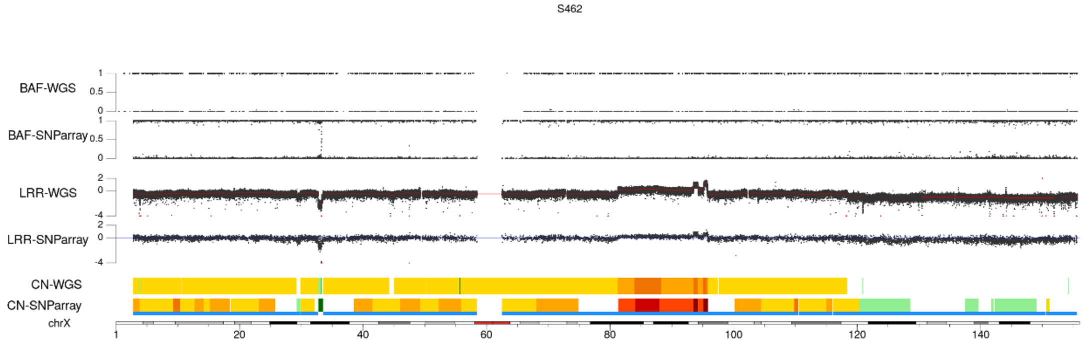
S462
BAF-WGS
BAF-SNParray
LRR-WGS
LRR-SNParray
CN-WGS
CN-SNParray
chrX
1
20
40
60
80
100
120
140

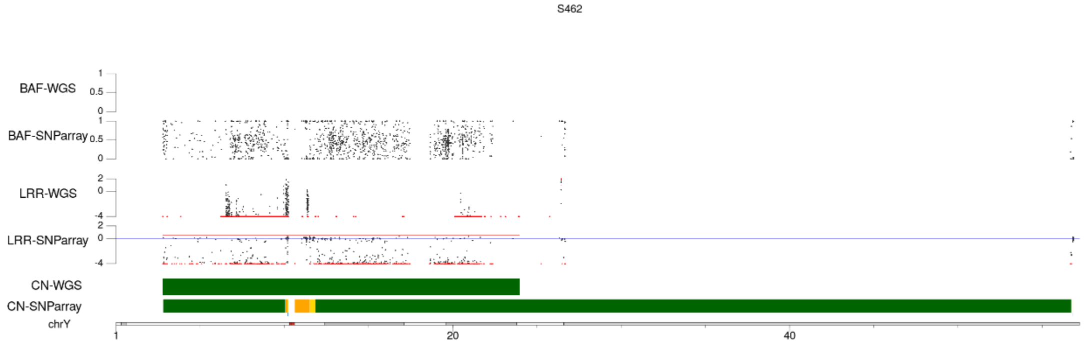
S462
BAF-WGS
BAF-SNParray
LRR-WGS
LRR-SNParray
CN-WGS
CN-SNParray
chrY
1
20
40

ST88-14

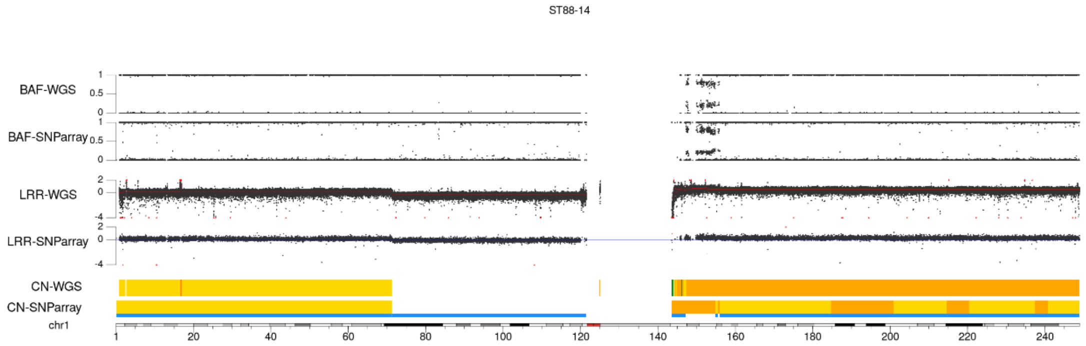
ST88-14
BAF-WGS
BAF-SNParray
LRR-WGS
LRR-SNParray
CN-WGS
CN-SNParray
chr1
1 20 40 60 80 100 120 140 160 180 200 220 240

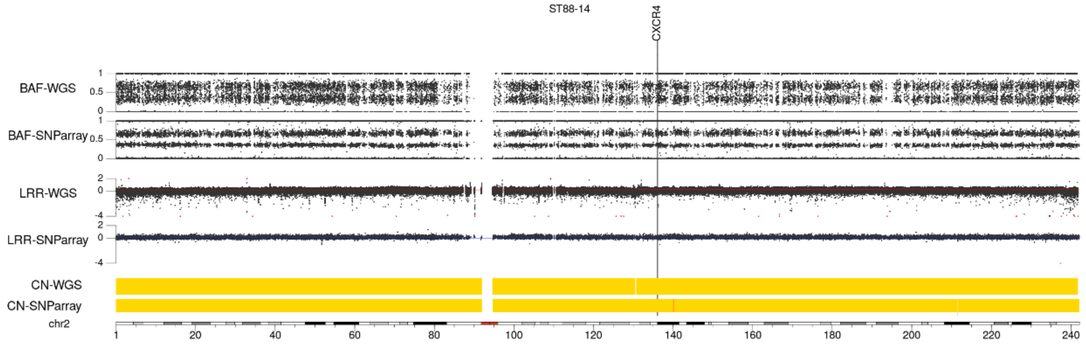
ST88-14
CXCR4
BAF-WGS
BAF-SNParray
LRR-WGS
LRR-SNParray
CN-WGS
CN-SNParray
chr2
1 20 40 60 80 100 120 140 160 180 200 220 240

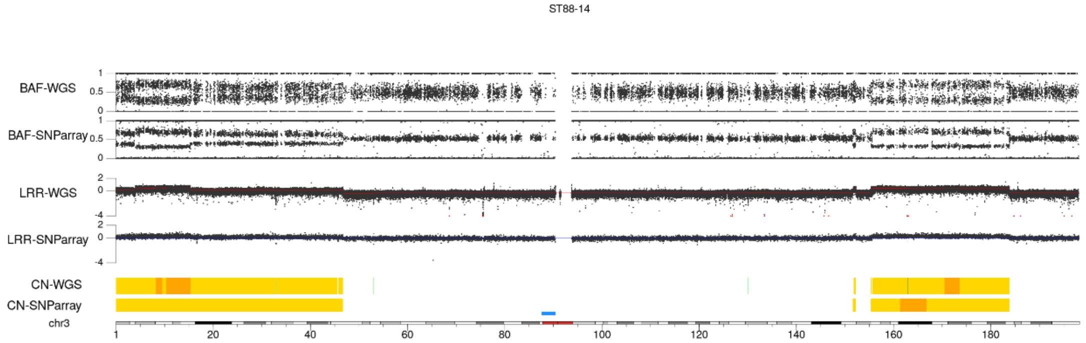
ST88-14
BAF-WGS
BAF-SNParray
LRR-WGS
LRR-SNParray
CN-WGS
CN-SNParray
chr3
1 20 40 60 80 100 120 140 160 180

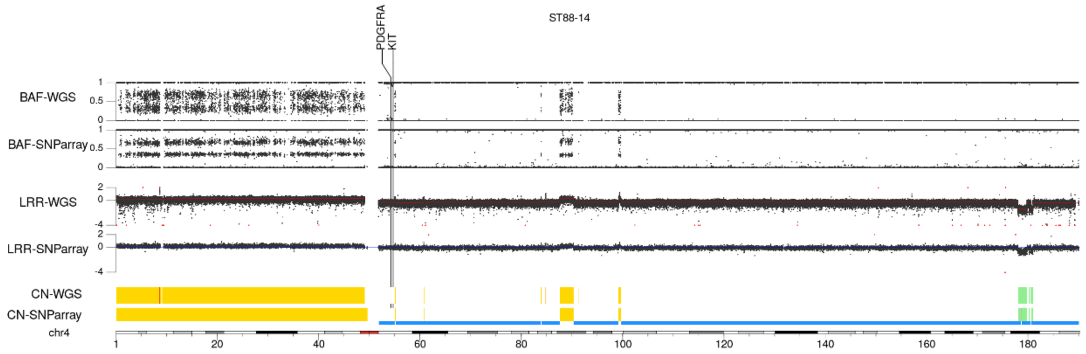
ST88-14
PDGFRA
KIT
BAF-WGS
BAF-SNParray
LRR-WGS
LRR-SNParray
CN-WGS
CN-SNParray
chr4
1 20 40 60 80 100 120 140 160 180

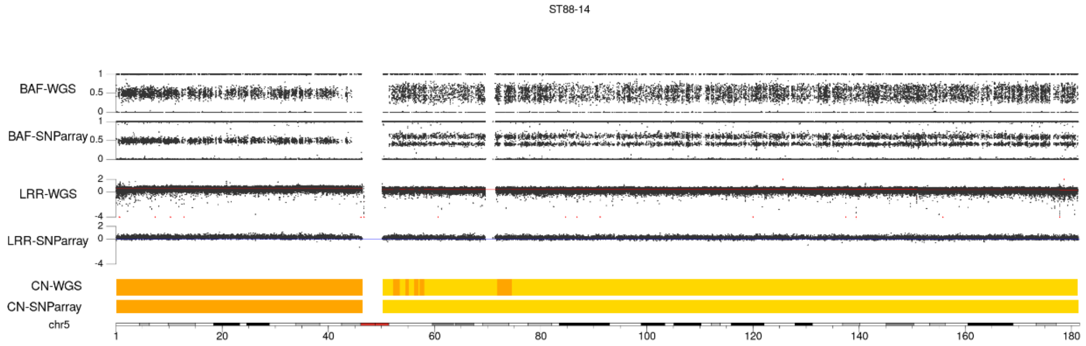
ST88-14
BAF-WGS
BAF-SNParray
LRR-WGS
LRR-SNParray
CN-WGS
CN-SNParray
chr5
1
20
40
60
80
100
120
140
160
180

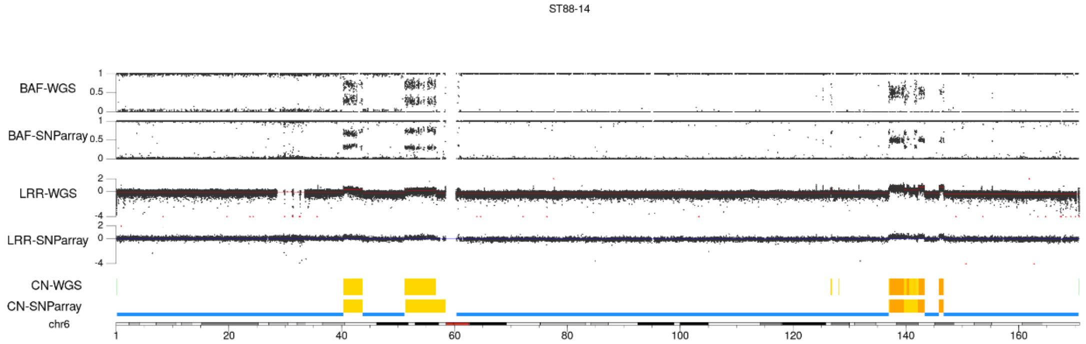
ST88-14
BAF-WGS
BAF-SNParray
LRR-WGS
LRR-SNParray
CN-WGS
CN-SNParray
chr6
1
20
40
60
80
100
120
140
160

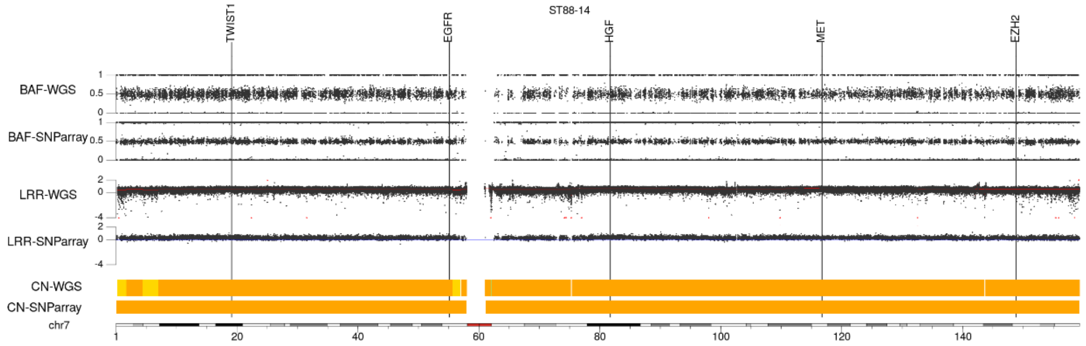
ST88-14
TWIST1
EGFR
HGF
MET
EZH2
BAF-WGS
BAF-SNParray
LRR-WGS
LRR-SNParray
CN-WGS
CN-SNParray
chr7
1
20
40
60
80
100
120
140

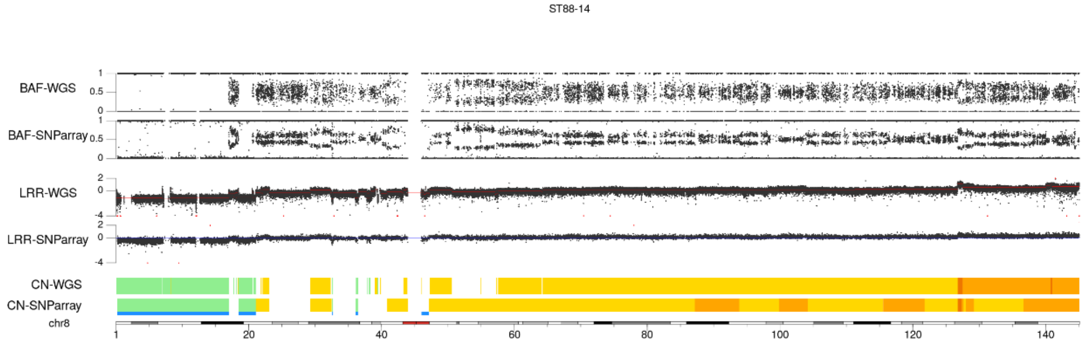
ST88-14
BAF-WGS
BAF-SNParray
LRR-WGS
LRR-SNParray
CN-WGS
CN-SNParray
chr8
1
20
40
60
80
100
120
140

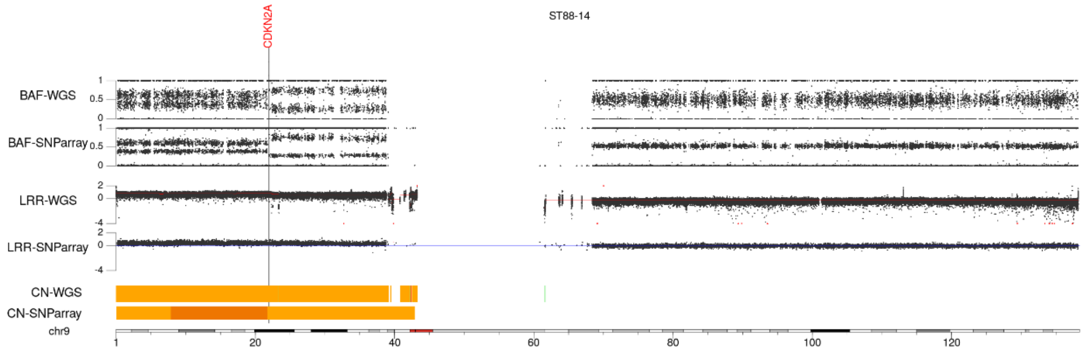
ST88-14
CDKN2A
BAF-WGS
BAF-SNParray
LRR-WGS
LRR-SNParray
CN-WGS
CN-SNParray
chr9
1
20
40
60
80
100
120

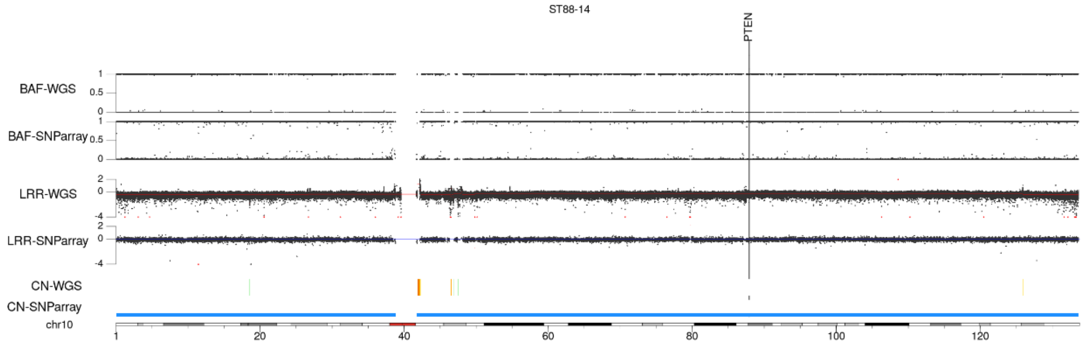
ST88-14
PTEN
BAF-WGS
BAF-SNParray
LRR-WGS
LRR-SNParray
CN-WGS
CN-SNParray
chr10
1 20 40 60 80 100 120

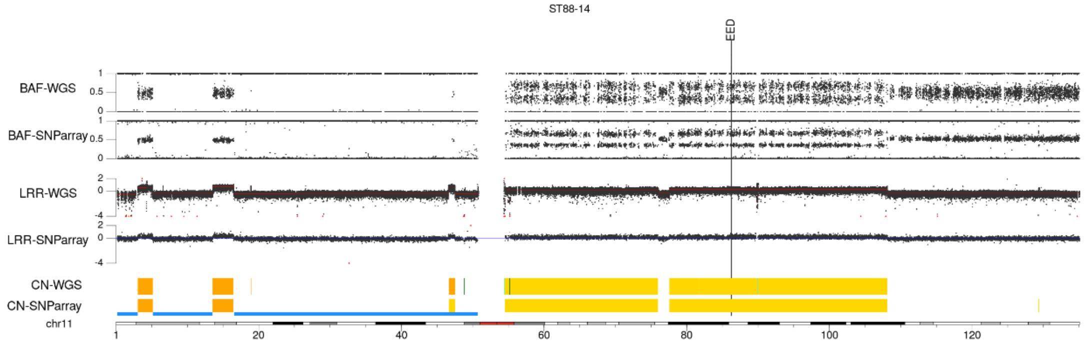
ST88-14
EED
BAF-WGS
BAF-SNParray
LRR-WGS
LRR-SNParray
CN-WGS
CN-SNParray
chr11
1 20 40 60 80 100 120

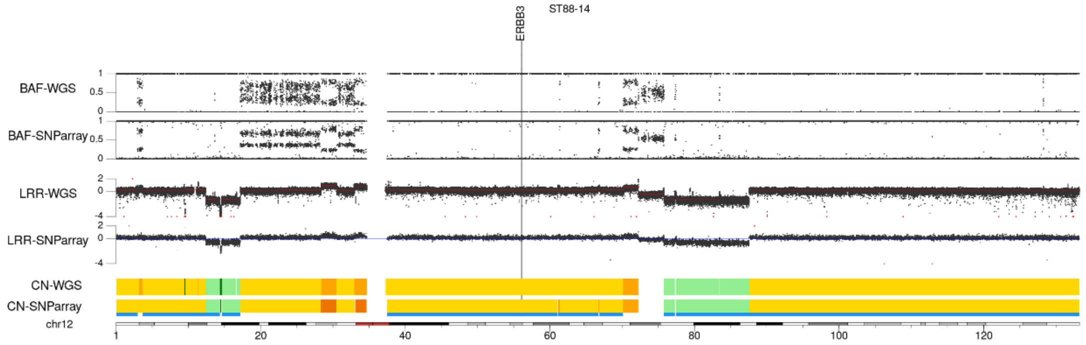
ST88-14
ERBB3
BAF-WGS
BAF-SNParray
LRR-WGS
LRR-SNParray
CN-WGS
CN-SNParray
chr12
1 20 40 60 80 100 120

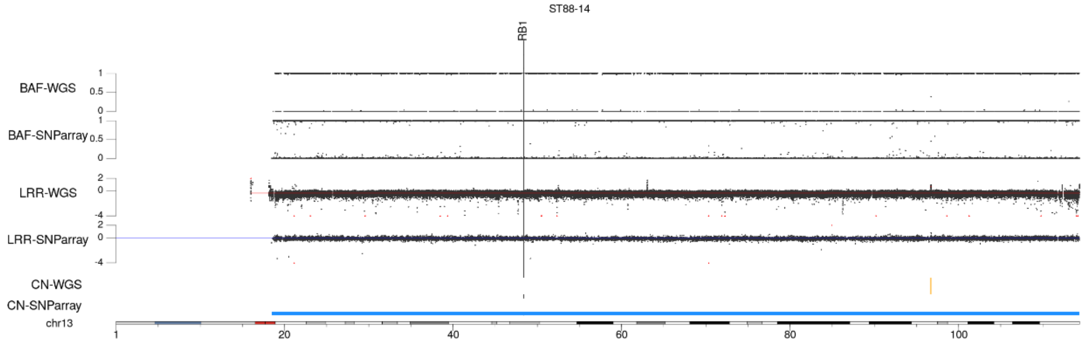
ST88-14
RB1
BAF-WGS
BAF-SNParray
LRR-WGS
LRR-SNParray
CN-WGS
CN-SNParray
chr13
1 20 40 60 80 100

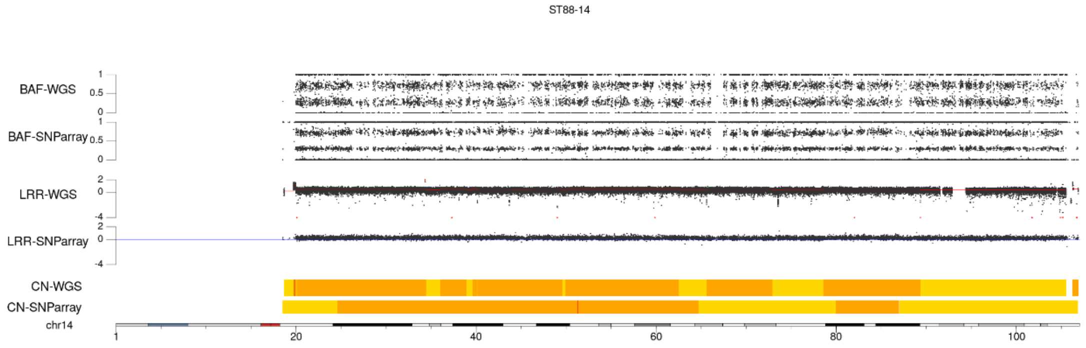
ST88-14
BAF-WGS
BAF-SNParray
LRR-WGS
LRR-SNParray
CN-WGS
CN-SNParray
chr14
1 20 40 60 80 100

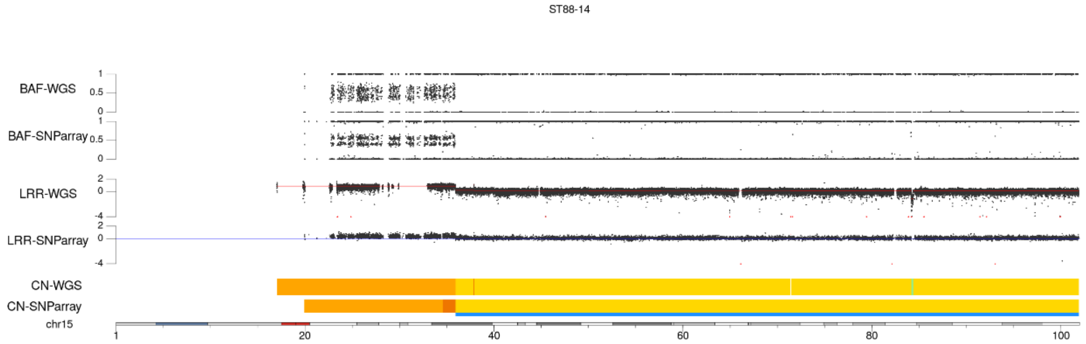
ST88-14
BAF-WGS
BAF-SNParray
LRR-WGS
LRR-SNParray
CN-WGS
CN-SNParray
chr15
1
20
40
60
80
100

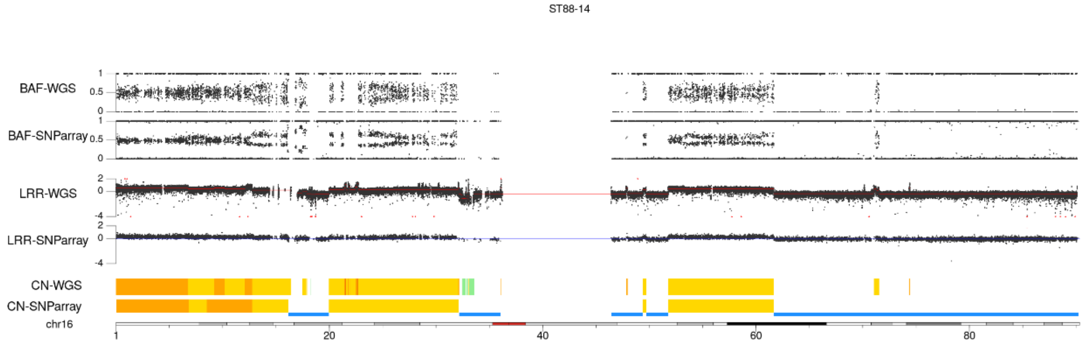
ST88-14
BAF-WGS
BAF-SNParray
LRR-WGS
LRR-SNParray
CN-WGS
CN-SNParray
chr16
1
20
40
60
80

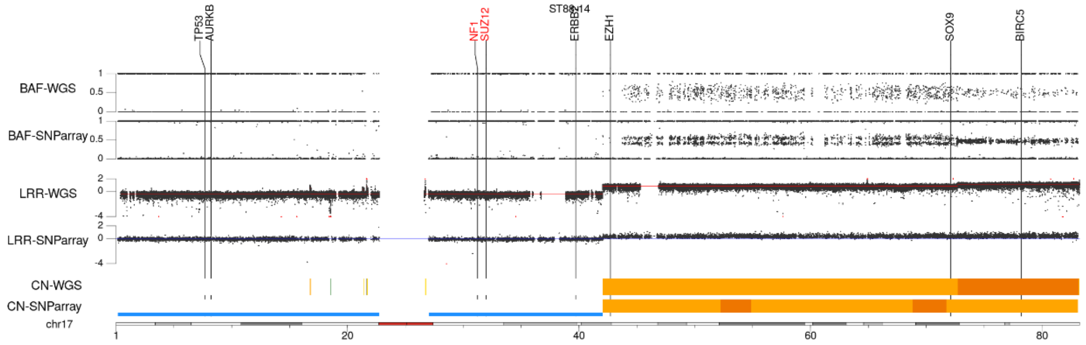
ST88-14
TP53
AURKB
NF1
SUZ12
ERBB2
EZH1
SOX9
BIRC5
BAF-WGS
BAF-SNParray
LRR-WGS
LRR-SNParray
CN-WGS
CN-SNParray
chr17
1
20
40
60
80

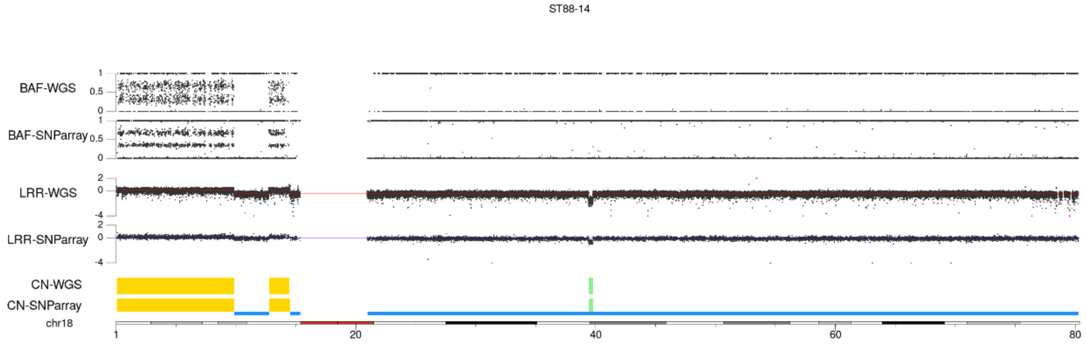
ST88-14
BAF-WGS
BAF-SNParray
LRR-WGS
LRR-SNParray
CN-WGS
CN-SNParray
chr18
1
20
40
60
80

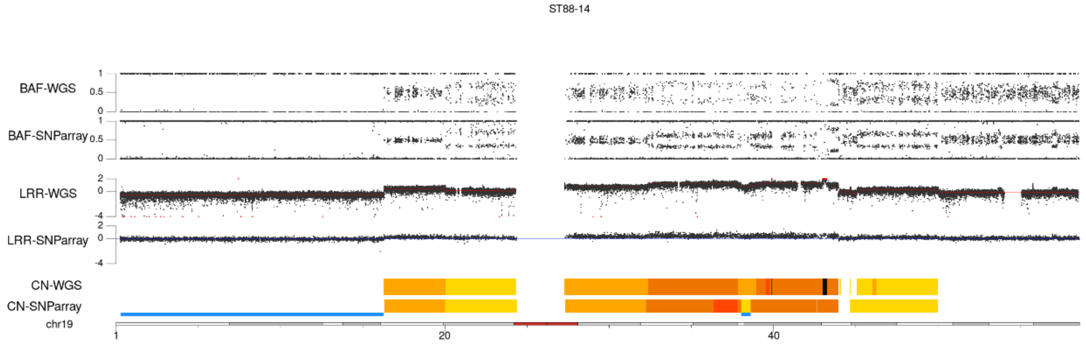
ST88-14
BAF-WGS
BAF-SNParray
LRR-WGS
LRR-SNParray
CN-WGS
CN-SNParray
chr19
1
20
40

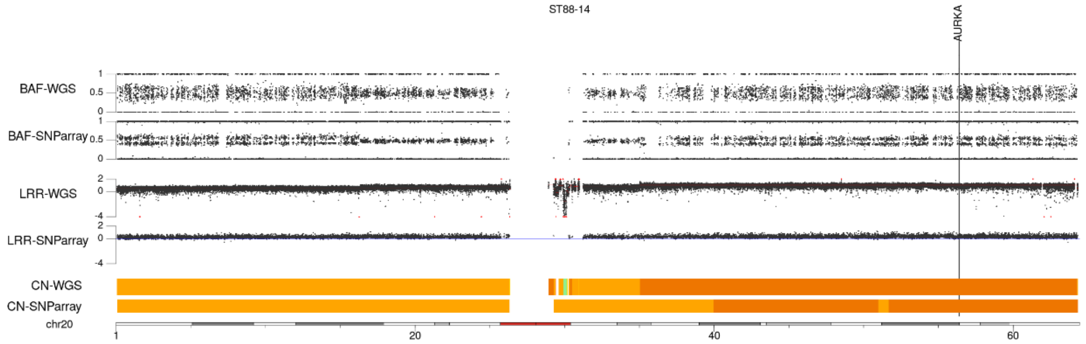
ST88-14
AURKA
BAF-WGS
BAF-SNParray
LRR-WGS
LRR-SNParray
CN-WGS
CN-SNParray
chr20
1
20
40
60

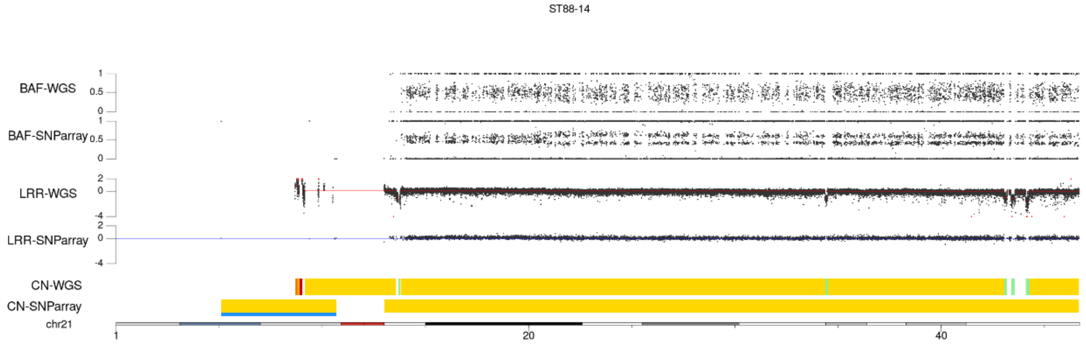
ST88-14
BAF-WGS
BAF-SNParray
LRR-WGS
LRR-SNParray
CN-WGS
CN-SNParray
chr21
1
20
40

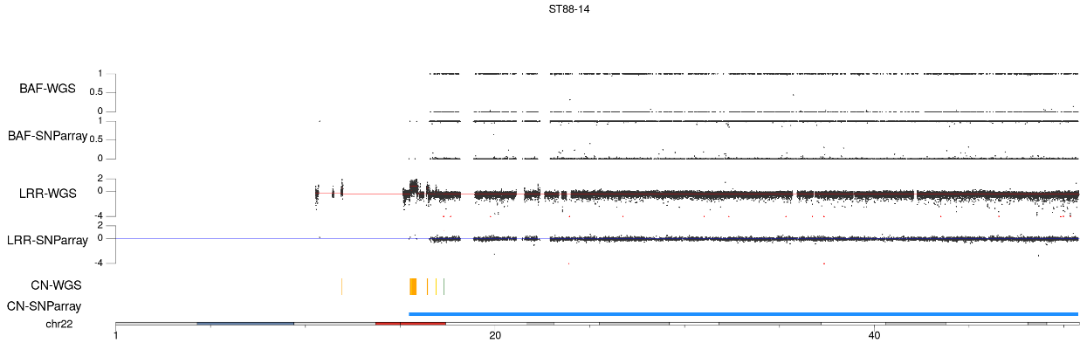
ST88-14
BAF-WGS
BAF-SNParray
LRR-WGS
LRR-SNParray
CN-WGS
CN-SNParray
chr22
1
20
40

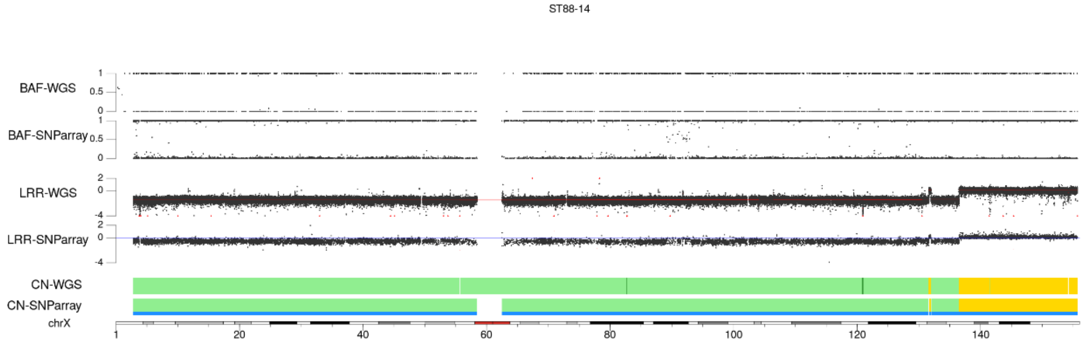
ST88-14
BAF-WGS
BAF-SNParray
LRR-WGS
LRR-SNParray
CN-WGS
CN-SNParray
chrX
1
20
40
60
80
100
120
140

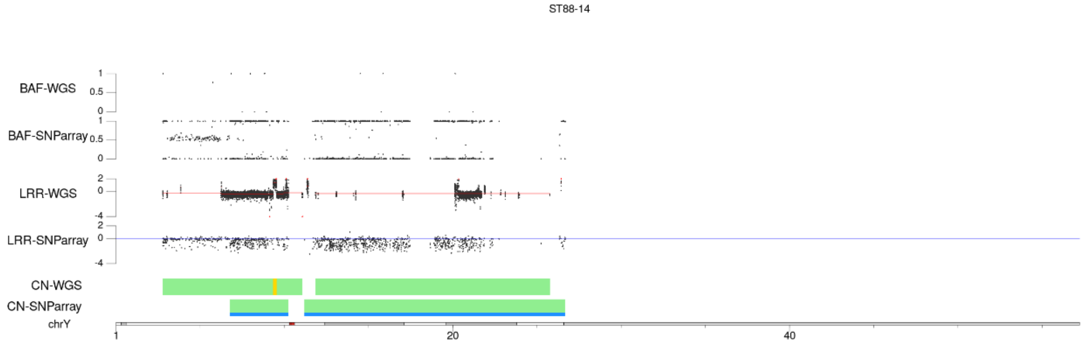
ST88-14
BAF-WGS
BAF-SNParray
LRR-WGS
LRR-SNParray
CN-WGS
CN-SNParray
chrY
1
20
40

NF90-8

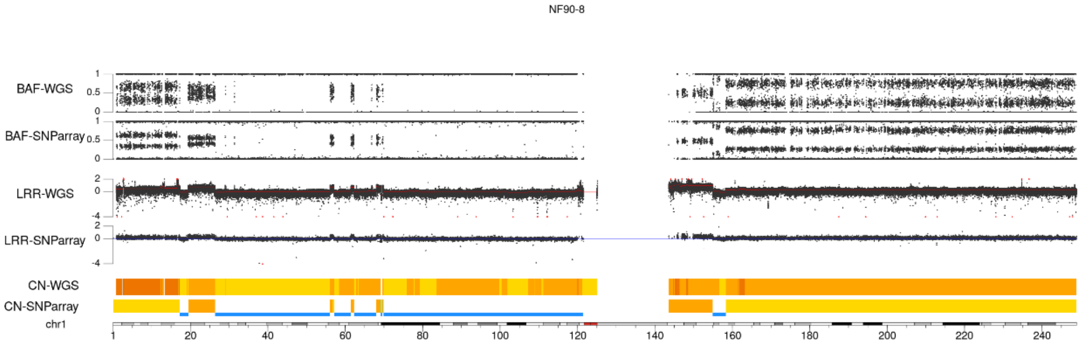
NF90-8
BAF-WGS
BAF-SNParray
LRR-WGS
LRR-SNParray
CN-WGS
CN-SNParray
chr1

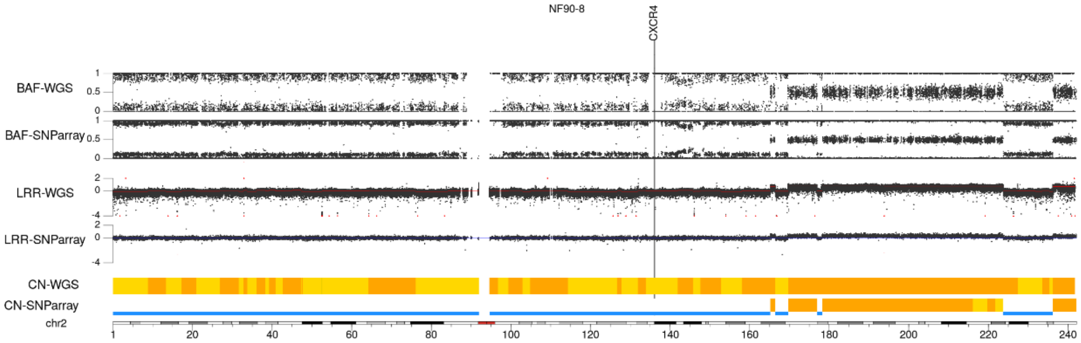
NF90-8
CXCR4
BAF-WGS
BAF-SNParray
LRR-WGS
LRR-SNParray
CN-WGS
CN-SNParray
chr2

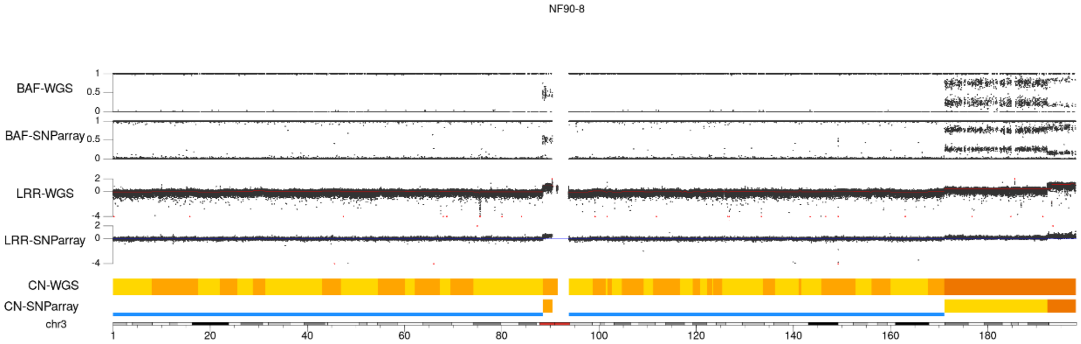
NF90-8
BAF-WGS
BAF-SNParray
LRR-WGS
LRR-SNParray
CN-WGS
CN-SNParray
chr3

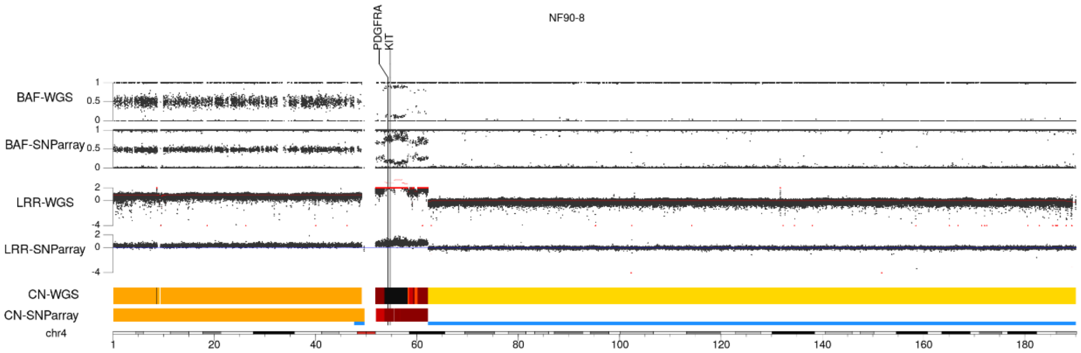
NF90-8
PDGFRA
KIT
BAF-WGS
BAF-SNParray
LRR-WGS
LRR-SNParray
CN-WGS
CN-SNParray
chr4

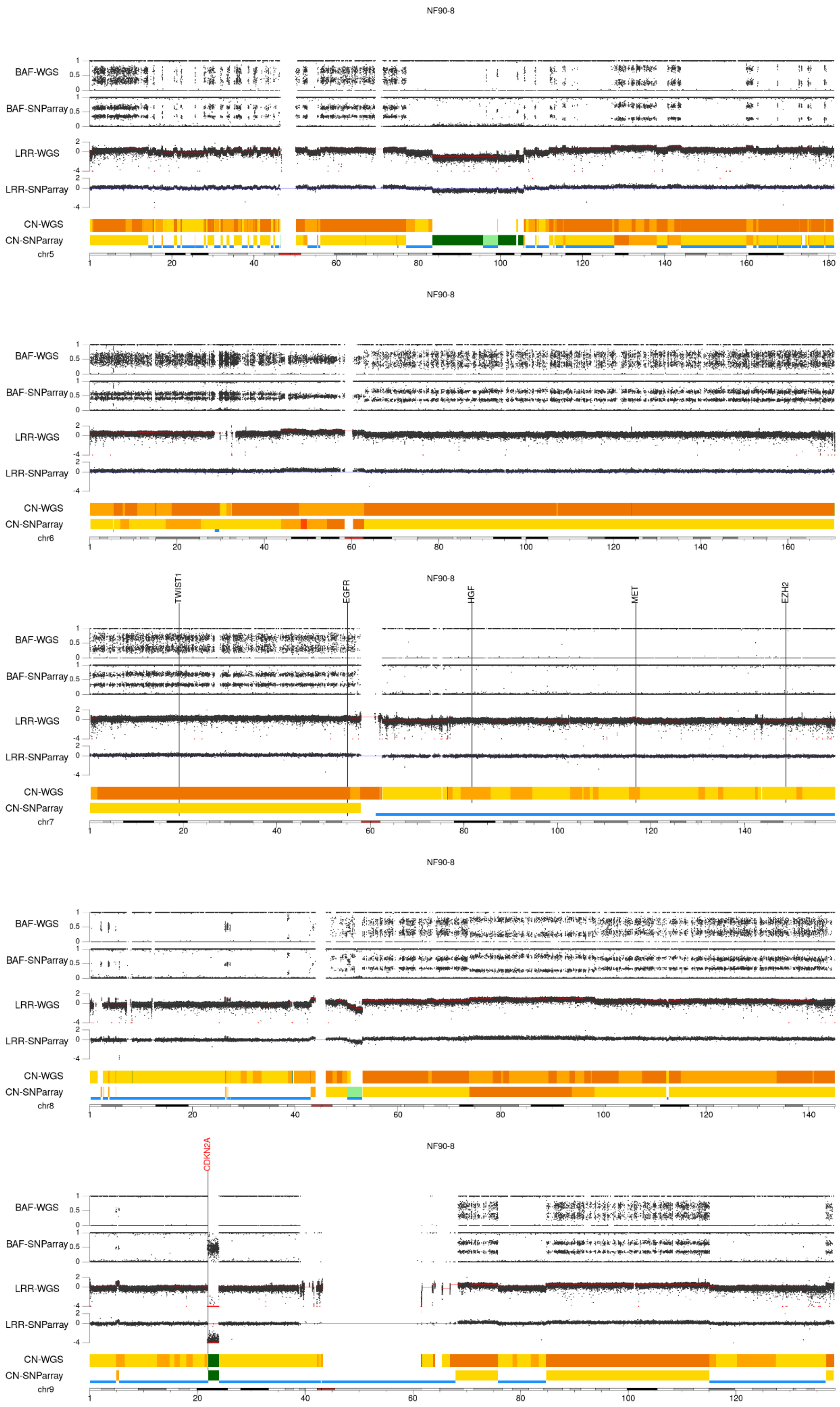
NF90-8
BAF-WGS
BAF-SNParray
LRR-WGS
LRR-SNParray
CN-WGS
CN-SNParray
chr5
NF90-8
chr6
NF90-8
TWIST1
EGFR
HGF
MET
EZH2
chr7
NF90-8
chr8
NF90-8
CDKN2A
chr9

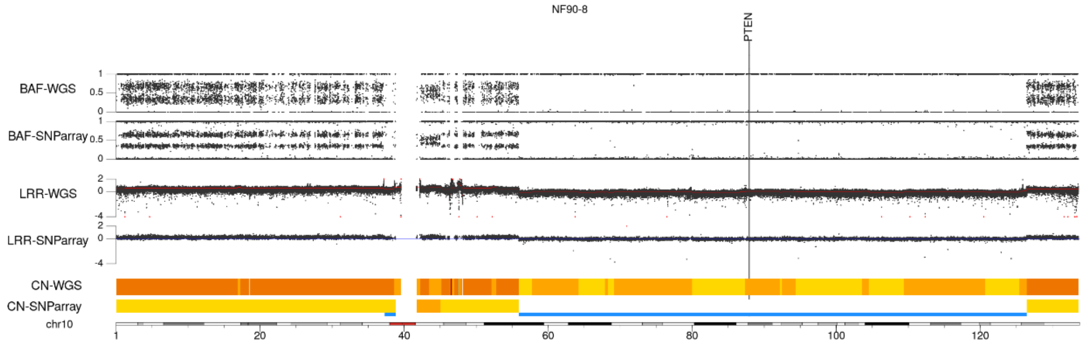
NF90-8
PTEN
BAF-WGS
BAF-SNParray
LRR-WGS
LRR-SNParray
CN-WGS
CN-SNParray
chr10
1 20 40 60 80 100 120

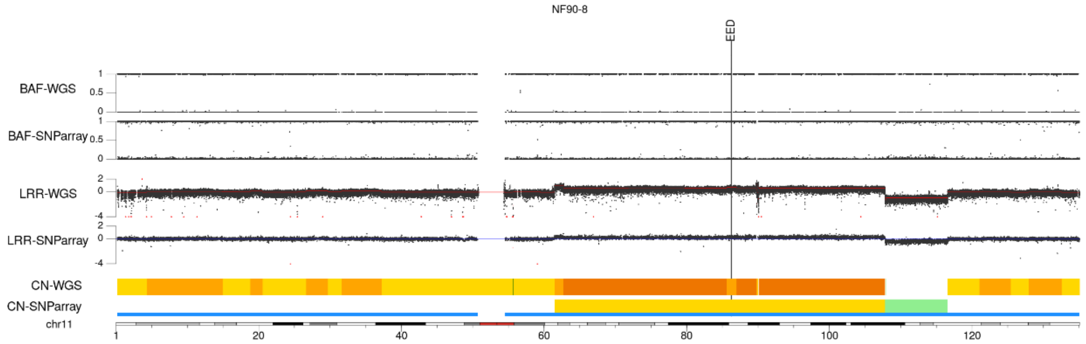
NF90-8
EED
BAF-WGS
BAF-SNParray
LRR-WGS
LRR-SNParray
CN-WGS
CN-SNParray
chr11
1 20 40 60 80 100 120

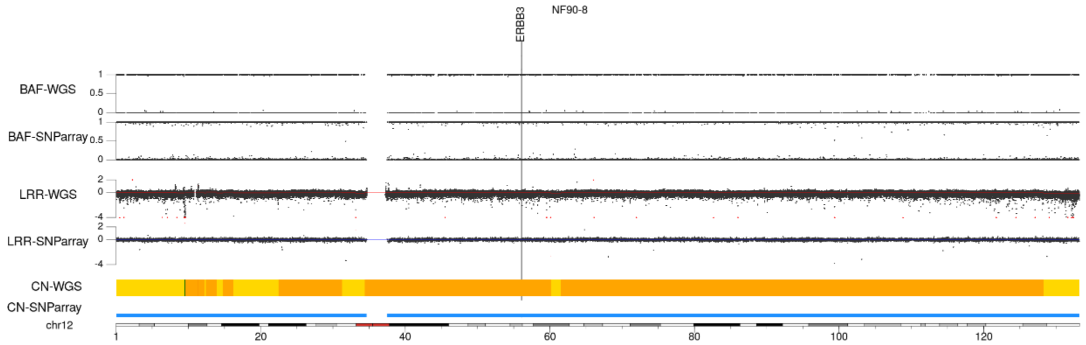
NF90-8
ERBB3
BAF-WGS
BAF-SNParray
LRR-WGS
LRR-SNParray
CN-WGS
CN-SNParray
chr12
1 20 40 60 80 100 120

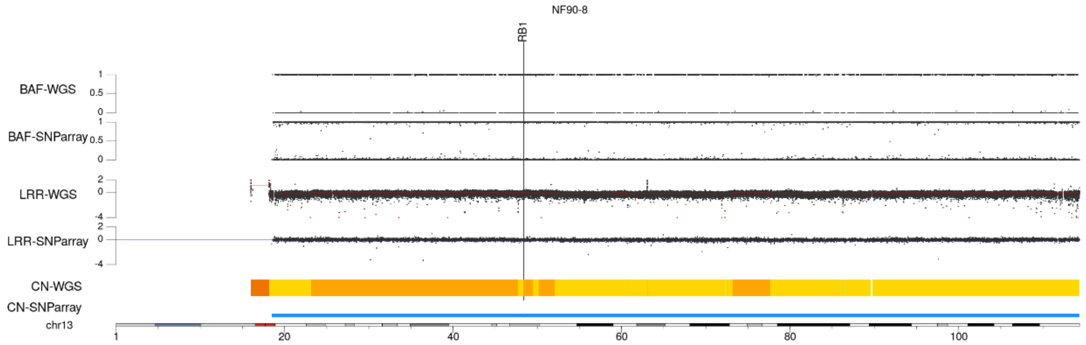
NF90-8
RB1
BAF-WGS
BAF-SNParray
LRR-WGS
LRR-SNParray
CN-WGS
CN-SNParray
chr13
1 20 40 60 80 100

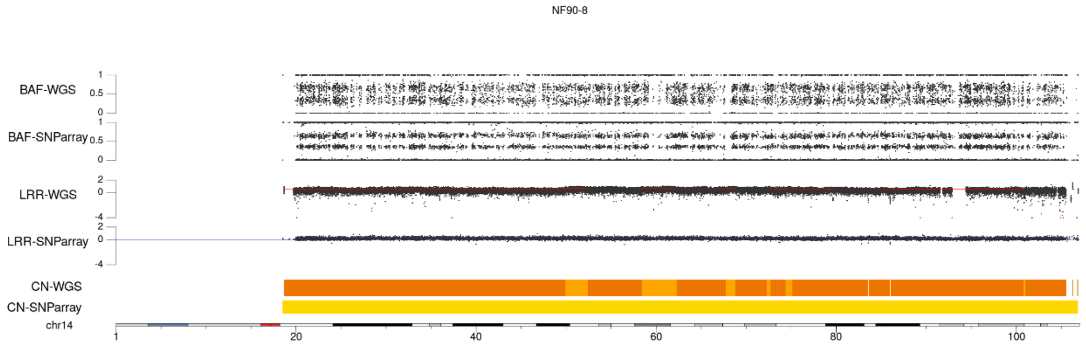
NF90-8
BAF-WGS
BAF-SNParray
LRR-WGS
LRR-SNParray
CN-WGS
CN-SNParray
chr14
1 20 40 60 80 100

NF90-8

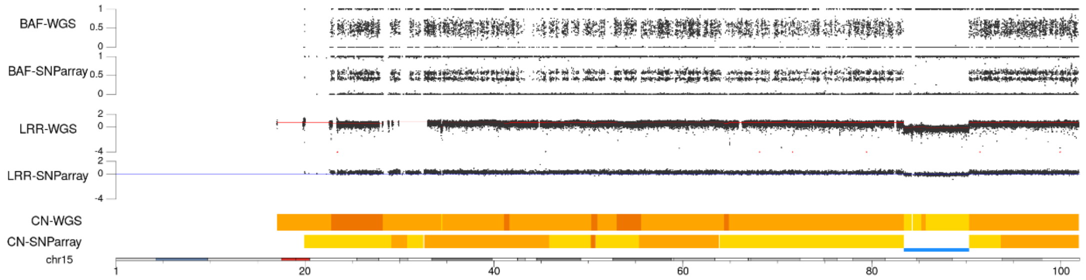

NF90-8

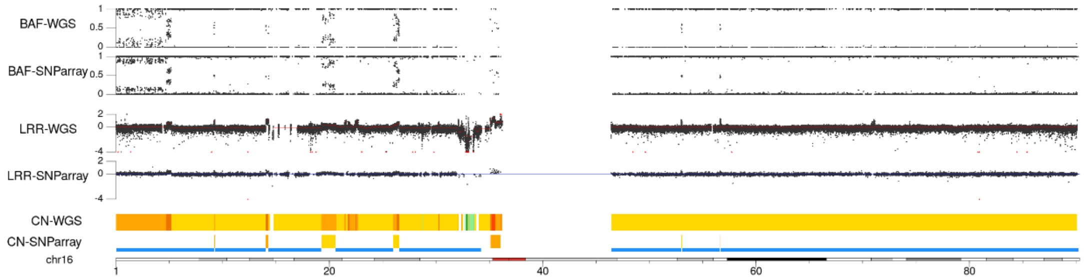

NF90-8

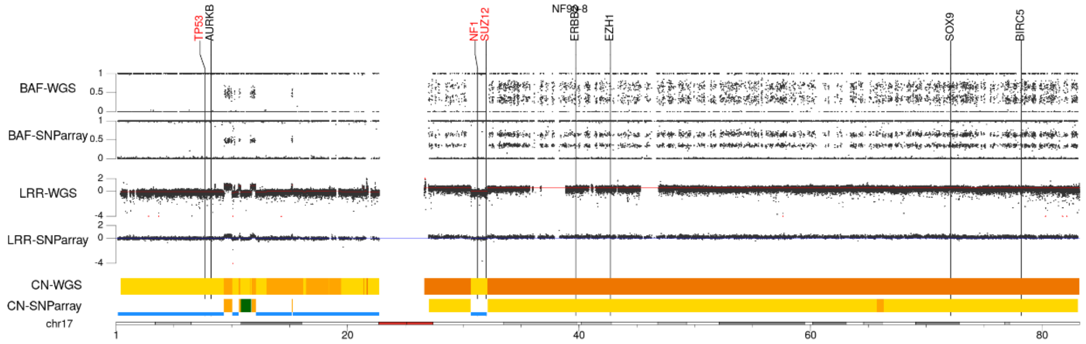

NF90-8

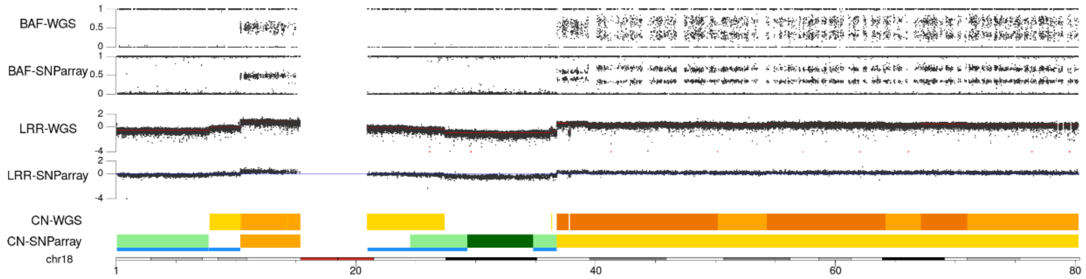

NF90-8

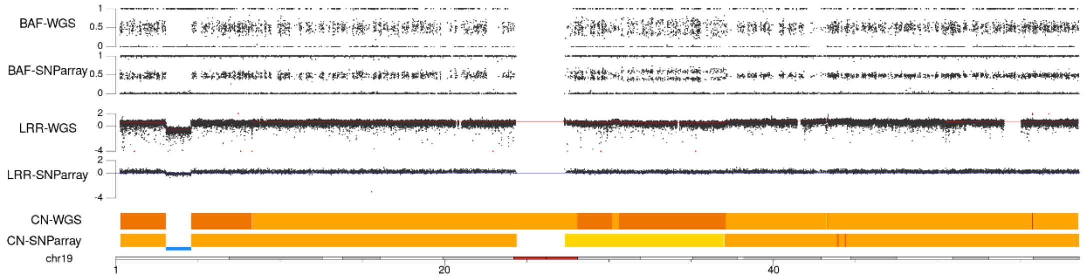

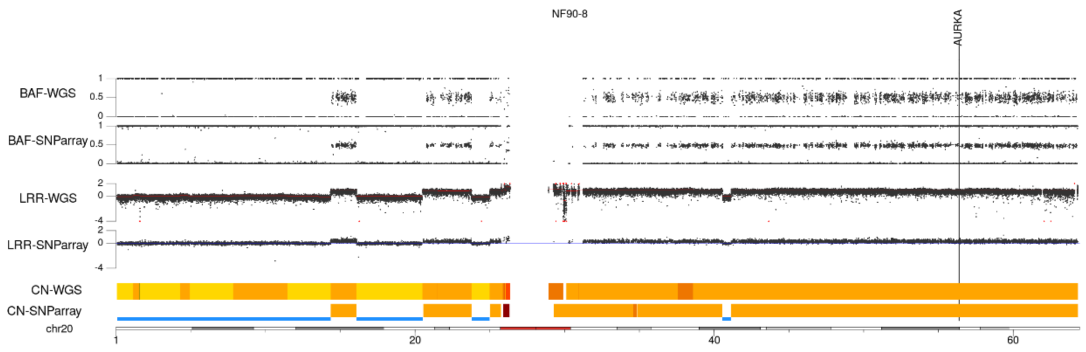
NF90-8
AURKA
BAF-WGS
BAF-SNParray
LRR-WGS
LRR-SNParray
CN-WGS
CN-SNParray
chr20
1
20
40
60

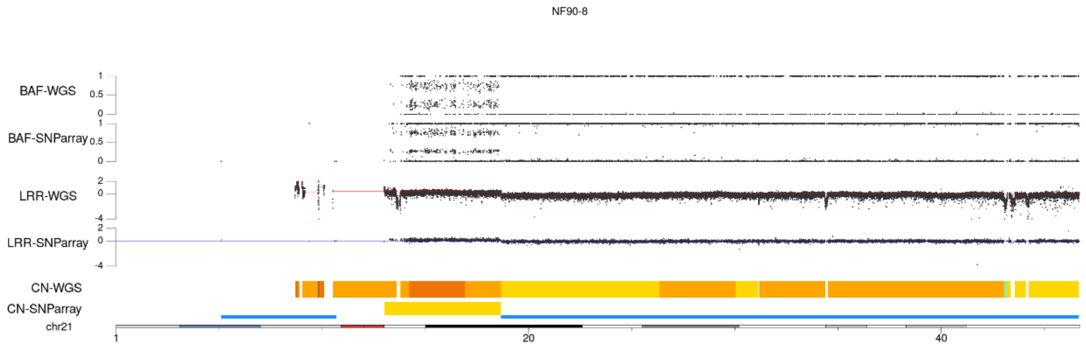
NF90-8
BAF-WGS
BAF-SNParray
LRR-WGS
LRR-SNParray
CN-WGS
CN-SNParray
chr21
1
20
40

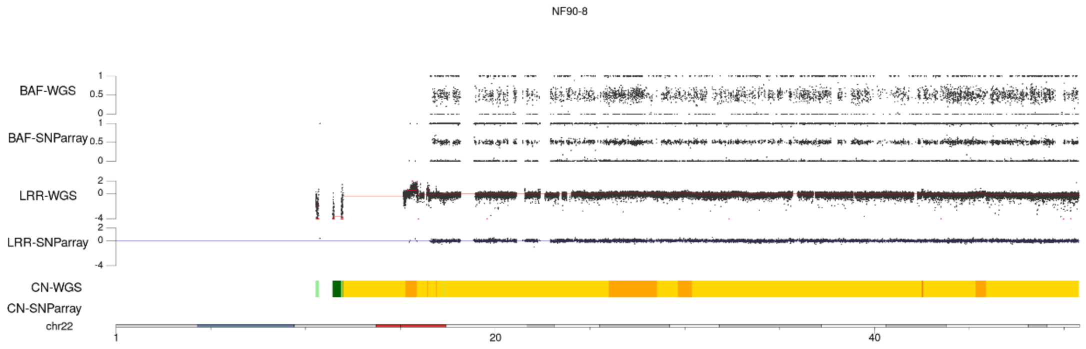
NF90-8
BAF-WGS
BAF-SNParray
LRR-WGS
LRR-SNParray
CN-WGS
CN-SNParray
chr22
1
20
40

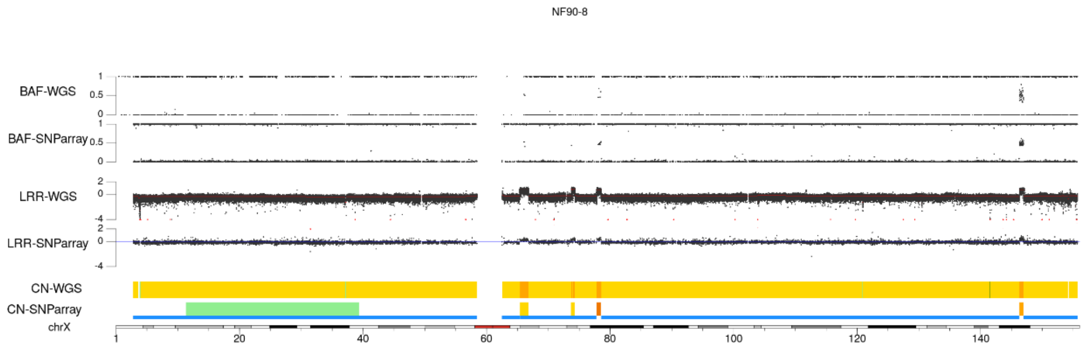
NF90-8
BAF-WGS
BAF-SNParray
LRR-WGS
LRR-SNParray
CN-WGS
CN-SNParray
chrX
1
20
40
60
80
100
120
140

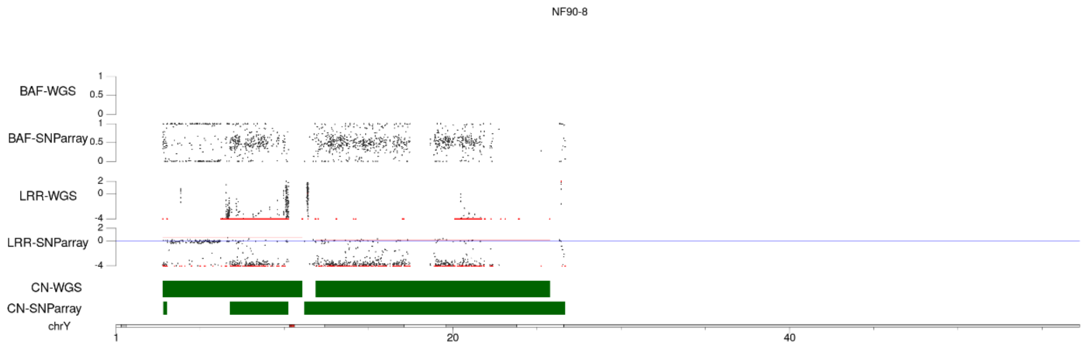
NF90-8
BAF-WGS
BAF-SNParray
LRR-WGS
LRR-SNParray
CN-WGS
CN-SNParray
chrY
1
20
40

SNF96-2

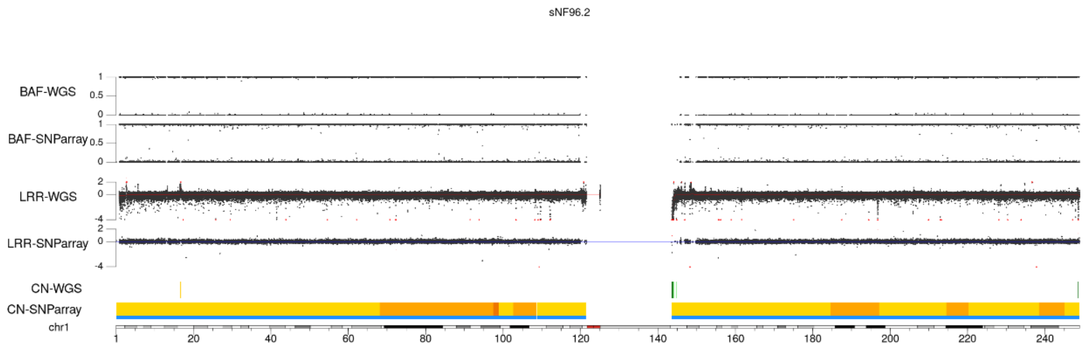
sNF96.2
BAF-WGS
BAF-SNParray
LRR-WGS
LRR-SNParray
CN-WGS
CN-SNParray
chr1
1 20 40 60 80 100 120 140 160 180 200 220 240

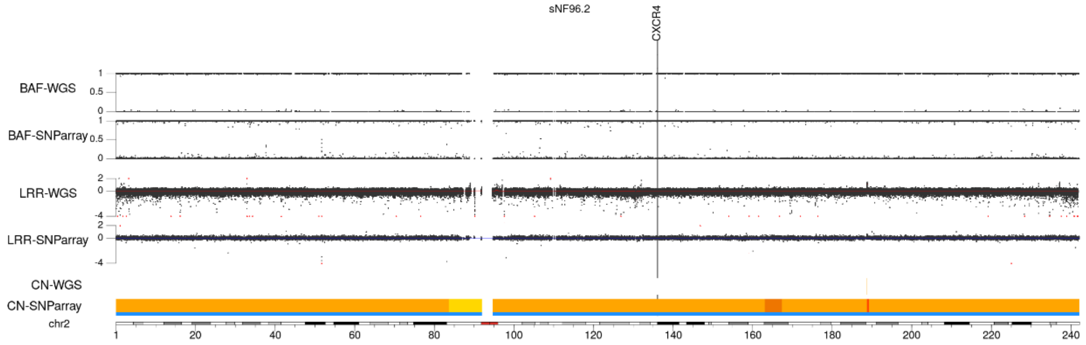
sNF96.2
CXCR4
BAF-WGS
BAF-SNParray
LRR-WGS
LRR-SNParray
CN-WGS
CN-SNParray
chr2
1 20 40 60 80 100 120 140 160 180 200 220 240

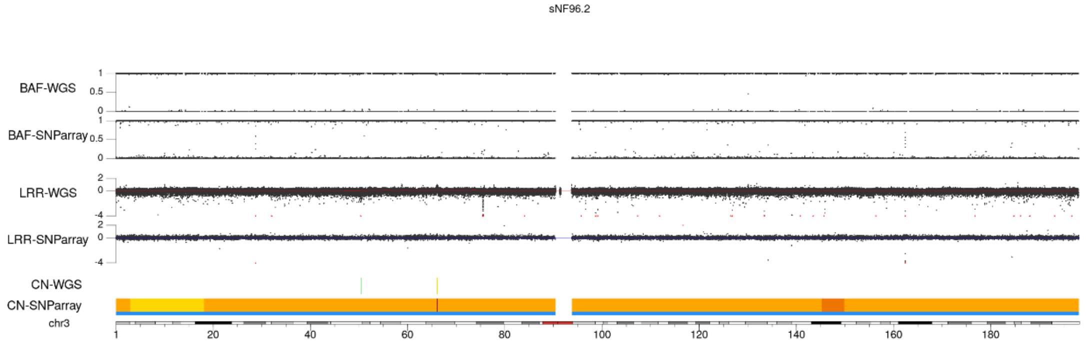
sNF96.2
BAF-WGS
BAF-SNParray
LRR-WGS
LRR-SNParray
CN-WGS
CN-SNParray
chr3
1 20 40 60 80 100 120 140 160 180

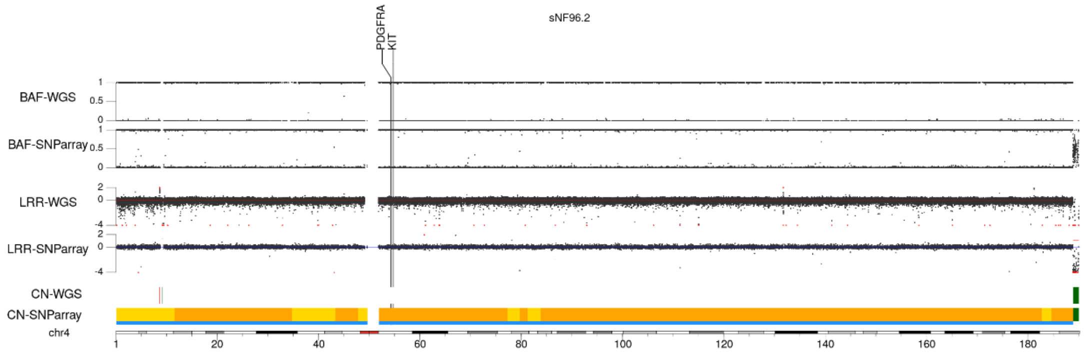
sNF96.2
PDGFRA
KIT
BAF-WGS
BAF-SNParray
LRR-WGS
LRR-SNParray
CN-WGS
CN-SNParray
chr4
1 20 40 60 80 100 120 140 160 180

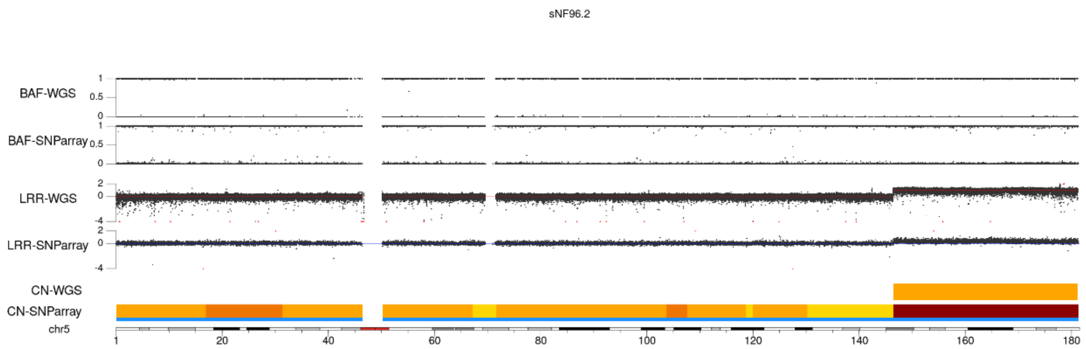
sNF96.2
BAF-WGS
BAF-SNParray
LRR-WGS
LRR-SNParray
CN-WGS
CN-SNParray
chr5

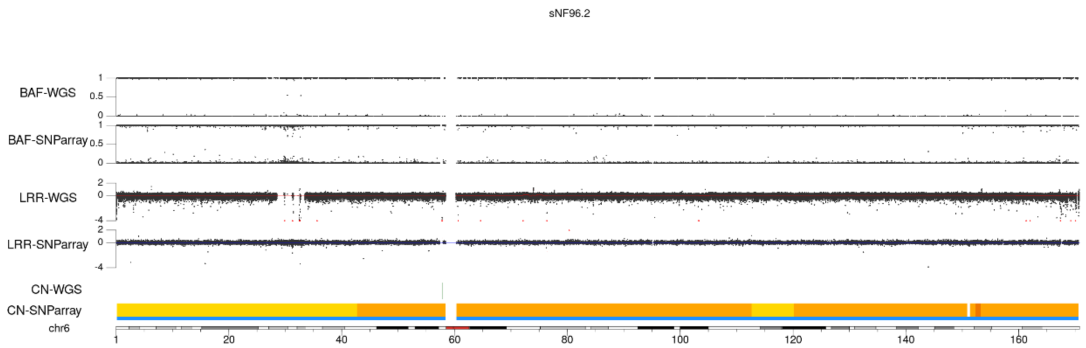
sNF96.2
BAF-WGS
BAF-SNParray
LRR-WGS
LRR-SNParray
CN-WGS
CN-SNParray
chr6

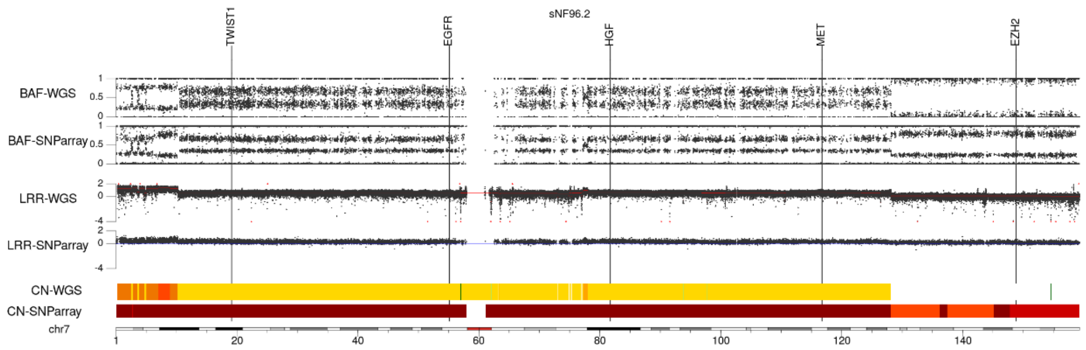
sNF96.2
TWIST1
EGFR
HGF
MET
EZH2
BAF-WGS
BAF-SNParray
LRR-WGS
LRR-SNParray
CN-WGS
CN-SNParray
chr7

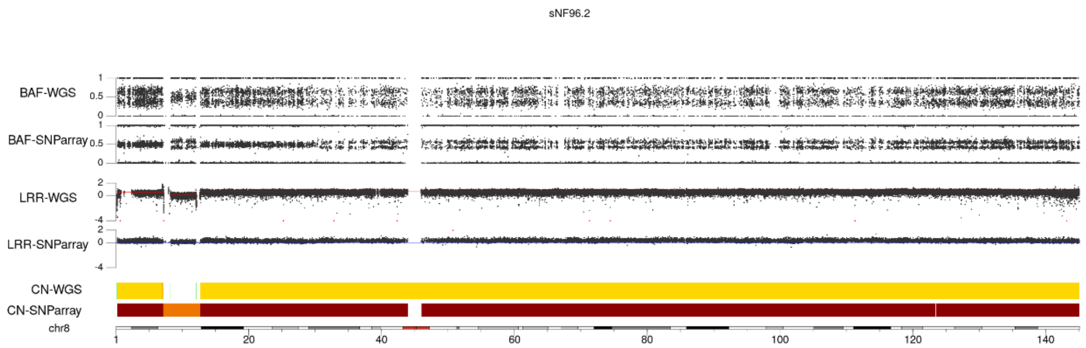
sNF96.2
BAF-WGS
BAF-SNParray
LRR-WGS
LRR-SNParray
CN-WGS
CN-SNParray
chr8

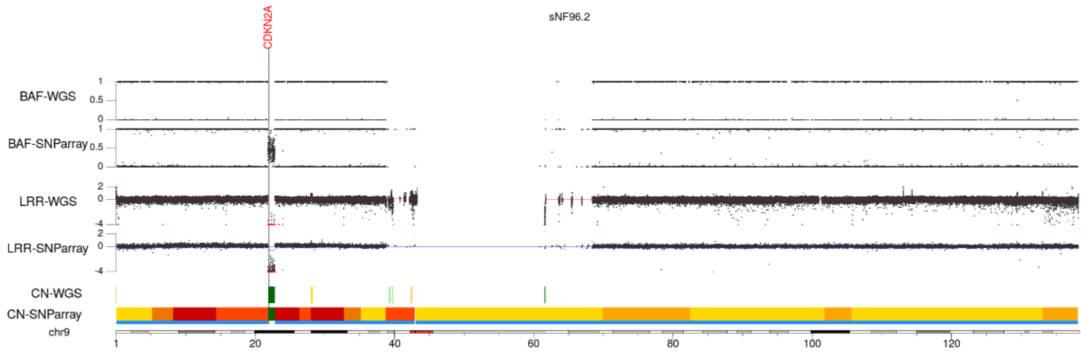
sNF96.2
CDKN2A
BAF-WGS
BAF-SNParray
LRR-WGS
LRR-SNParray
CN-WGS
CN-SNParray
chr9

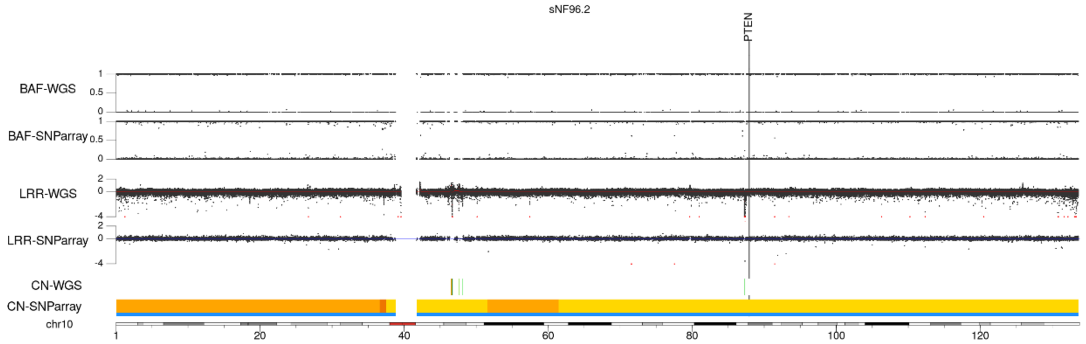
sNF96.2
PTEN
BAF-WGS
BAF-SNParray
LRR-WGS
LRR-SNParray
CN-WGS
CN-SNParray
chr10

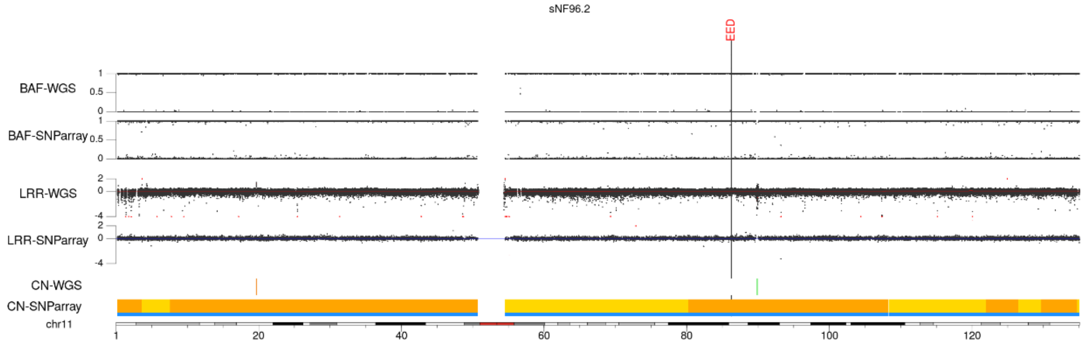
sNF96.2
EED
BAF-WGS
BAF-SNParray
LRR-WGS
LRR-SNParray
CN-WGS
CN-SNParray
chr11

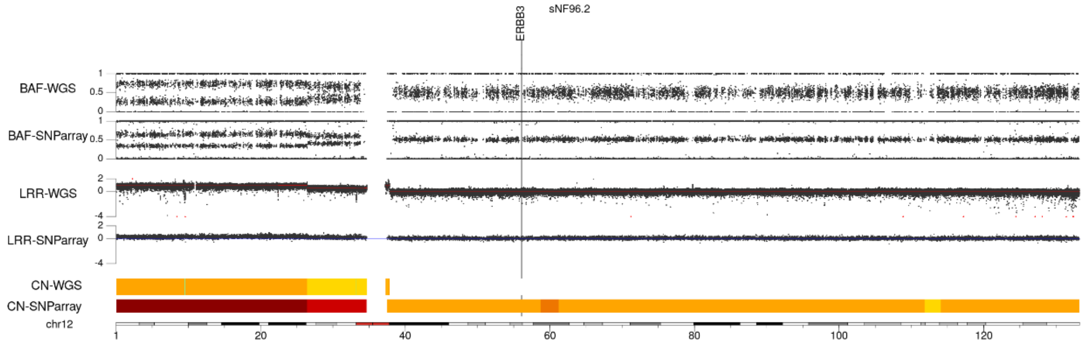
sNF96.2
ERBB3
BAF-WGS
BAF-SNParray
LRR-WGS
LRR-SNParray
CN-WGS
CN-SNParray
chr12

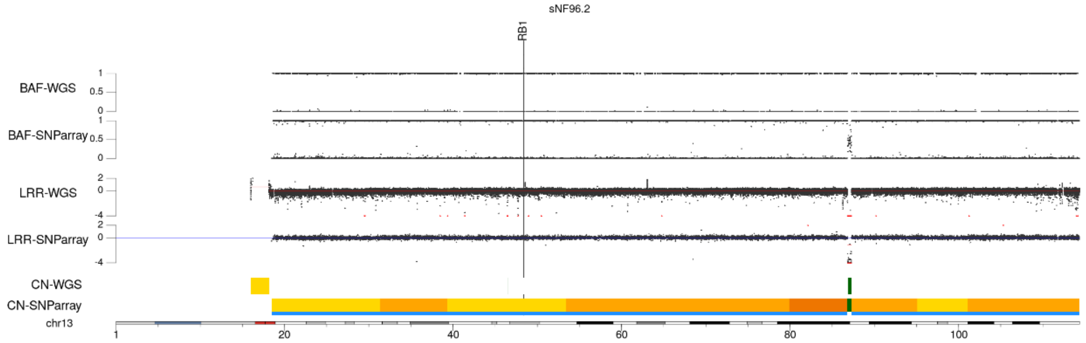
sNF96.2
RB1
BAF-WGS
BAF-SNParray
LRR-WGS
LRR-SNParray
CN-WGS
CN-SNParray
chr13

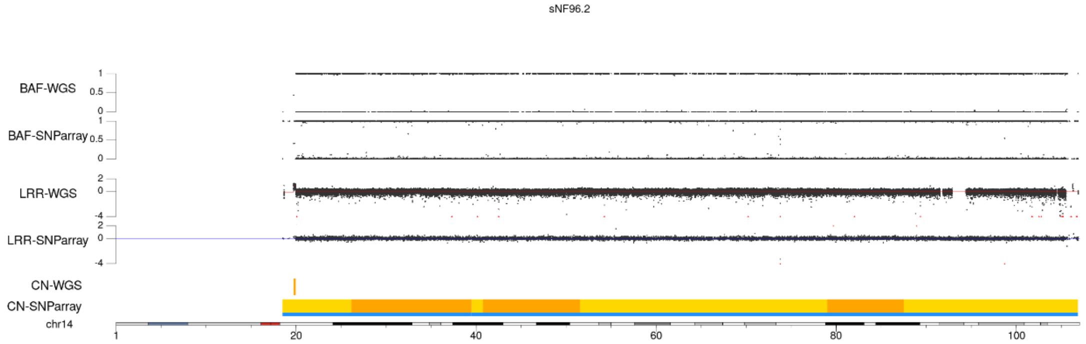
sNF96.2
BAF-WGS
BAF-SNParray
LRR-WGS
LRR-SNParray
CN-WGS
CN-SNParray
chr14

sNF96.2

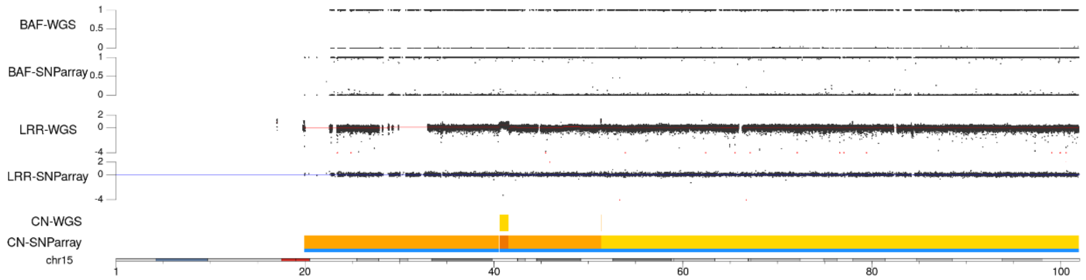

sNF96.2

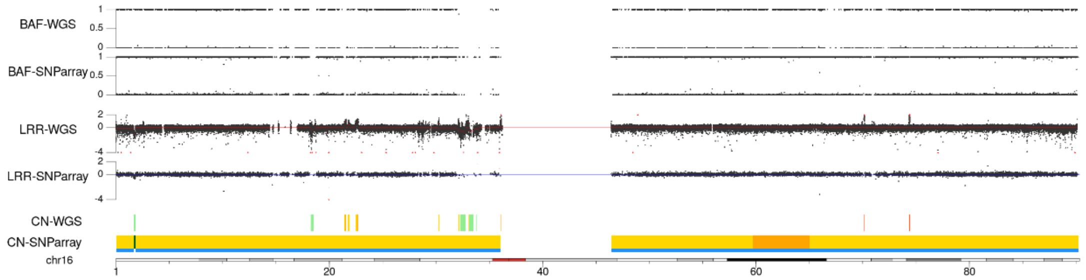

sNF96.2

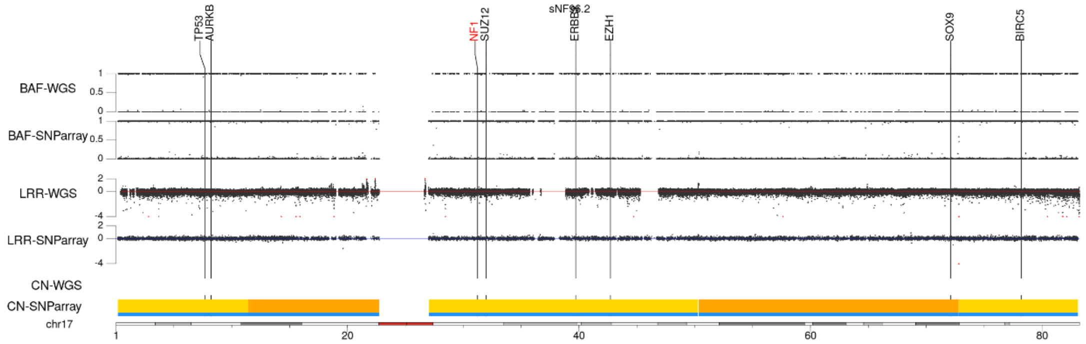

sNF96.2

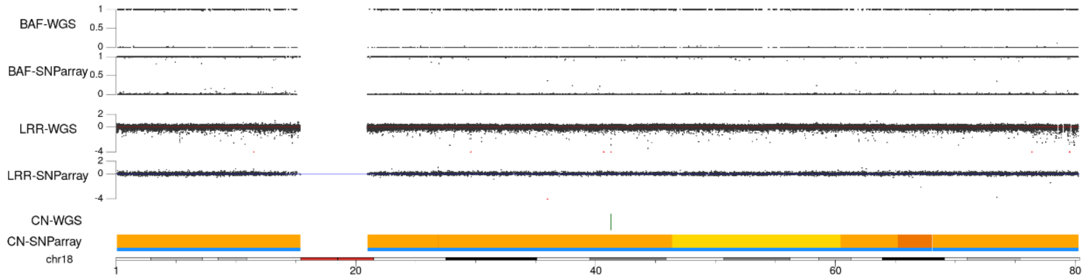

sNF96.2

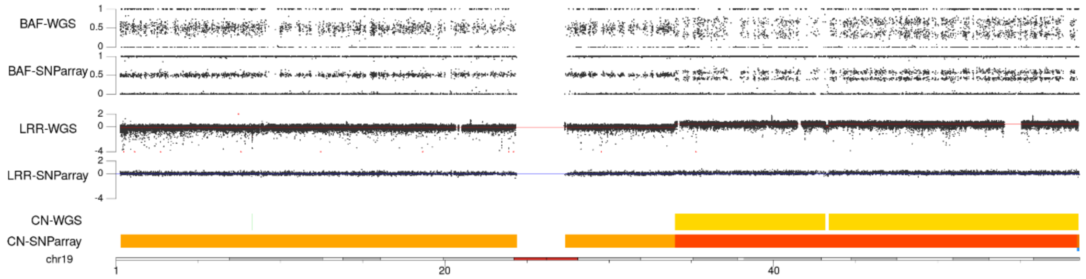

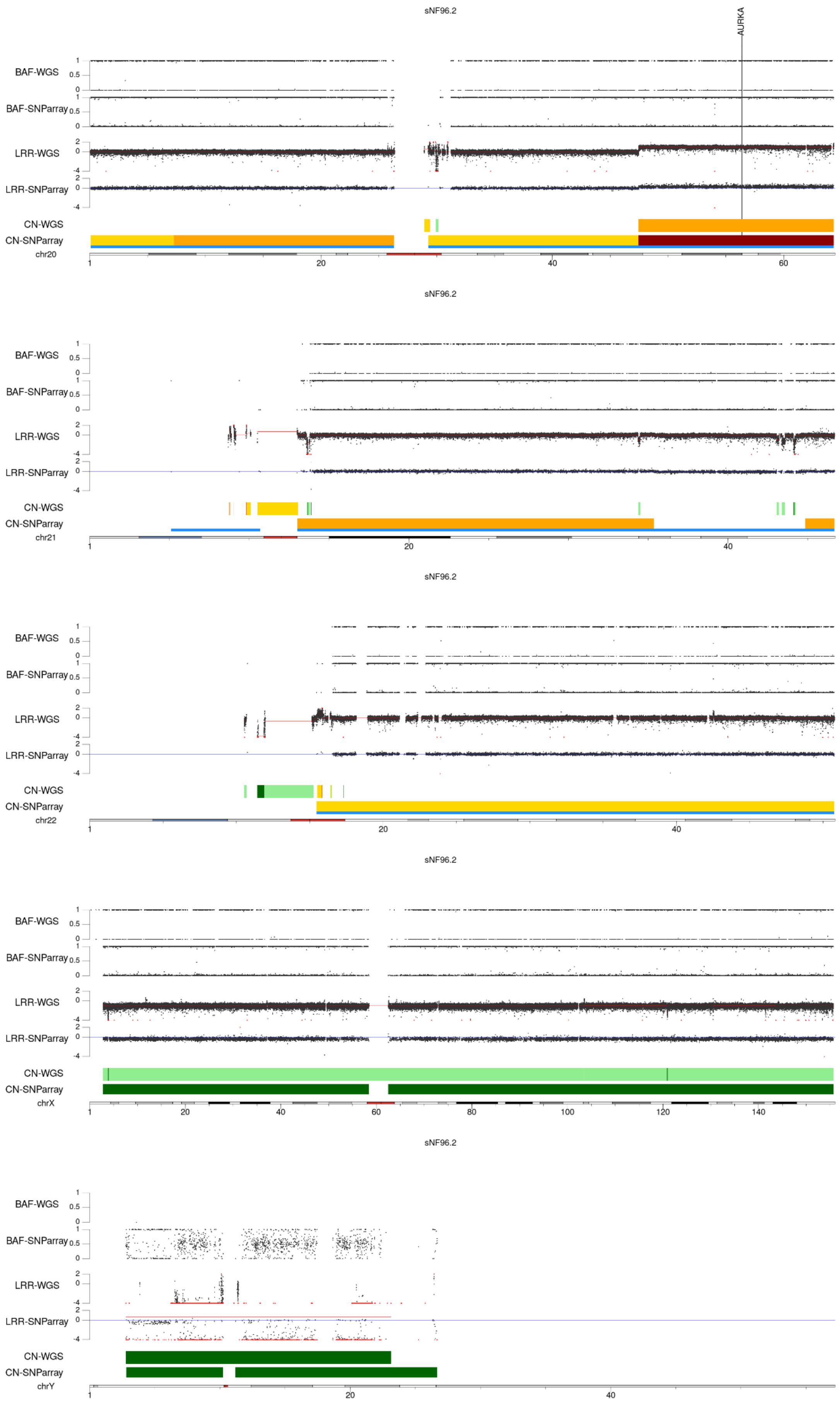
sNF96.2
AURKA
BAF-WGS
BAF-SNParray
LRR-WGS
LRR-SNParray
CN-WGS
CN-SNParray
chr20
sNF96.2
chr21
sNF96.2
chr22
sNF96.2
chrX
sNF96.2
chrY

NMS-2

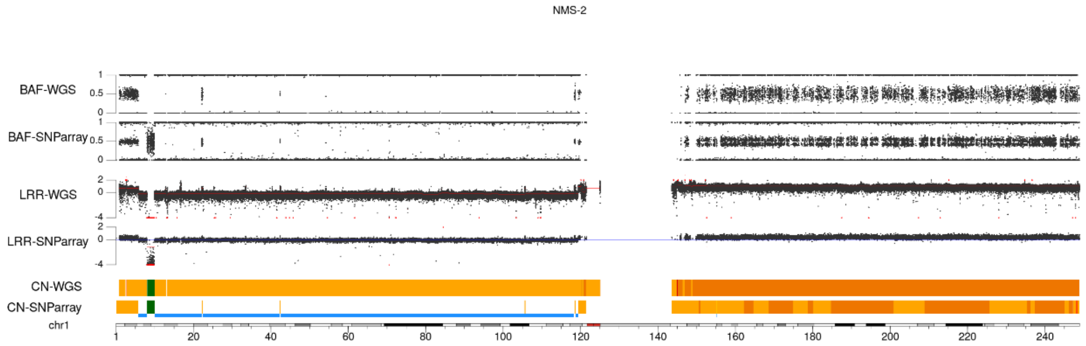
NMS-2
BAF-WGS
BAF-SNParray
LRR-WGS
LRR-SNParray
CN-WGS
CN-SNParray
chr1
1 20 40 60 80 100 120 140 160 180 200 220 240

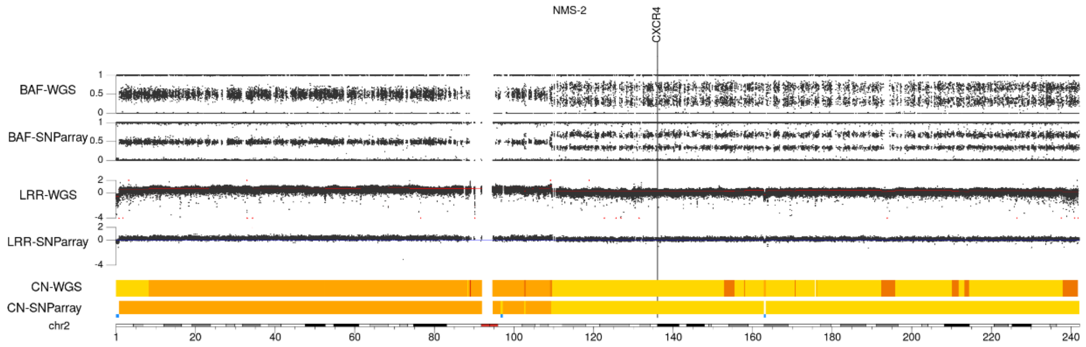
NMS-2
CXCR4
BAF-WGS
BAF-SNParray
LRR-WGS
LRR-SNParray
CN-WGS
CN-SNParray
chr2
1 20 40 60 80 100 120 140 160 180 200 220 240

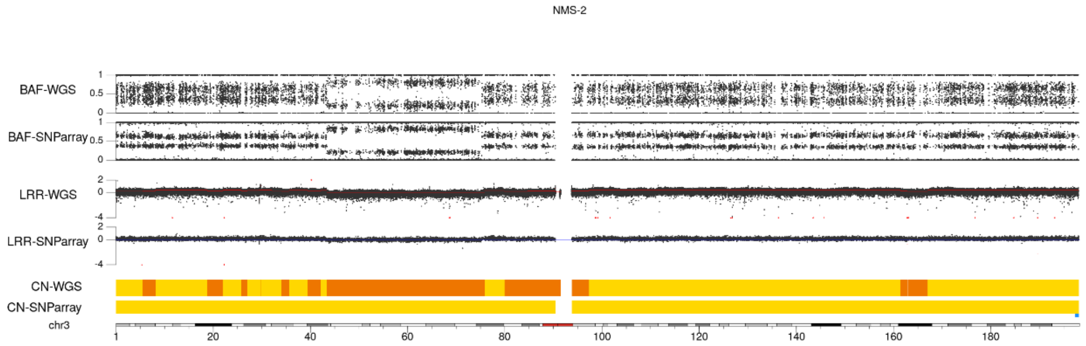
NMS-2
BAF-WGS
BAF-SNParray
LRR-WGS
LRR-SNParray
CN-WGS
CN-SNParray
chr3
1 20 40 60 80 100 120 140 160 180

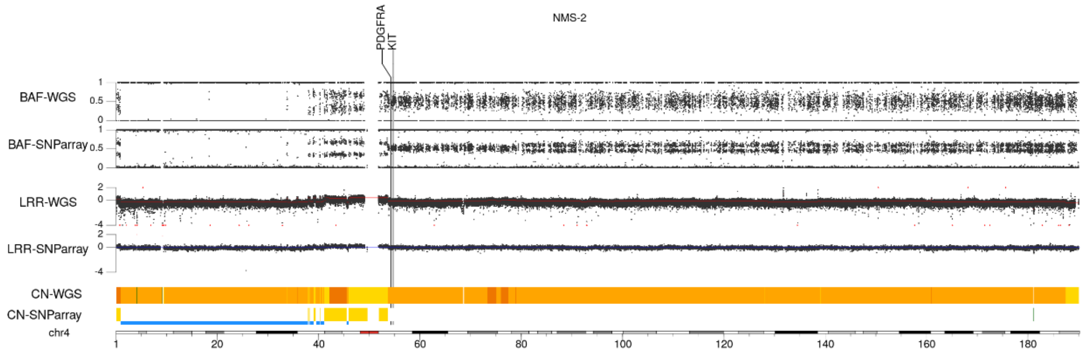
NMS-2
PDGFRA
KIT
BAF-WGS
BAF-SNParray
LRR-WGS
LRR-SNParray
CN-WGS
CN-SNParray
chr4
1 20 40 60 80 100 120 140 160 180

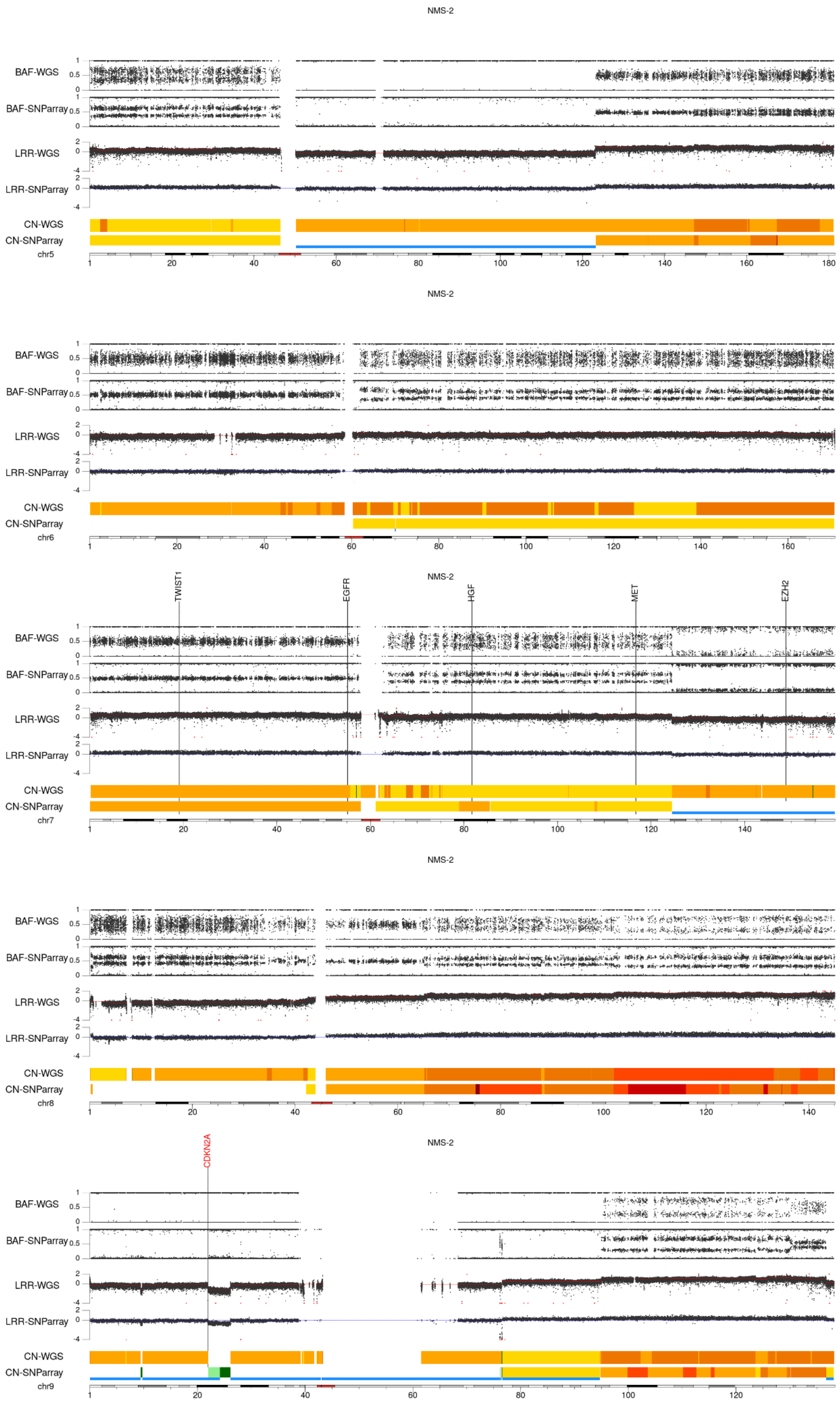
NMS-2
BAF-WGS
BAF-SNParray
LRR-WGS
LRR-SNParray
CN-WGS
CN-SNParray
chr5
NMS-2
chr6
NMS-2
TWIST1
EGFR
HGF
MET
EZH2
chr7
NMS-2
chr8
NMS-2
CDKN2A
chr9

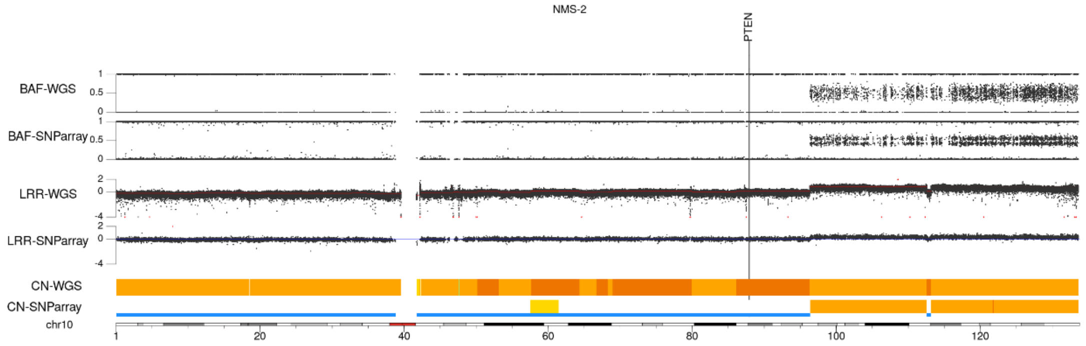
NMS-2
PTEN
BAF-WGS
BAF-SNParray
LRR-WGS
LRR-SNParray
CN-WGS
CN-SNParray
chr10
1 20 40 60 80 100 120

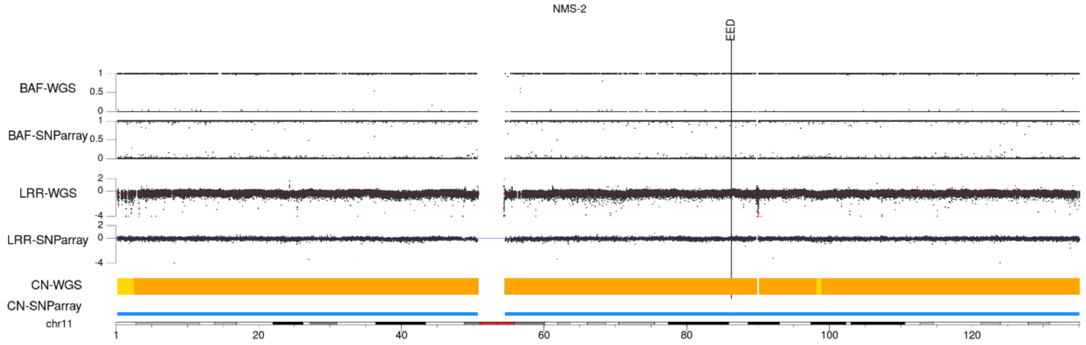
NMS-2
EED
BAF-WGS
BAF-SNParray
LRR-WGS
LRR-SNParray
CN-WGS
CN-SNParray
chr11
1 20 40 60 80 100 120

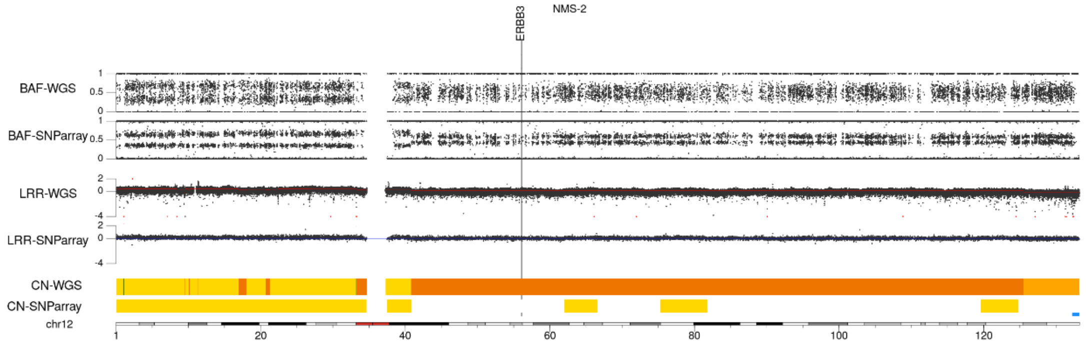
NMS-2
ERBB3
BAF-WGS
BAF-SNParray
LRR-WGS
LRR-SNParray
CN-WGS
CN-SNParray
chr12
1 20 40 60 80 100 120

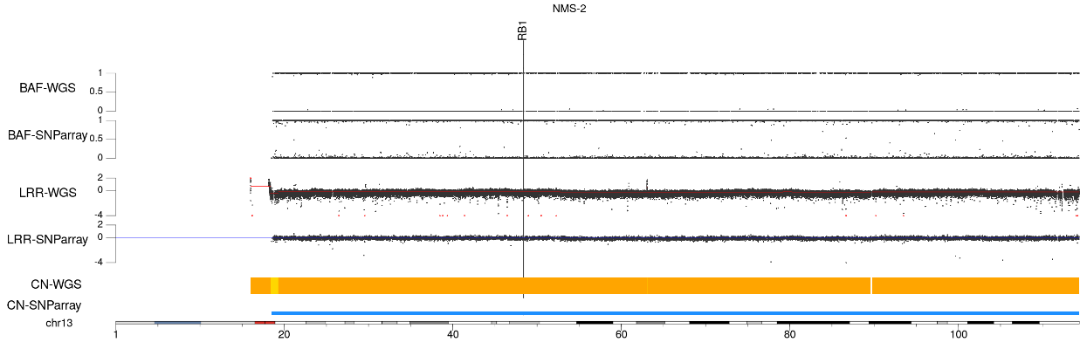
NMS-2
RB1
BAF-WGS
BAF-SNParray
LRR-WGS
LRR-SNParray
CN-WGS
CN-SNParray
chr13
1 20 40 60 80 100

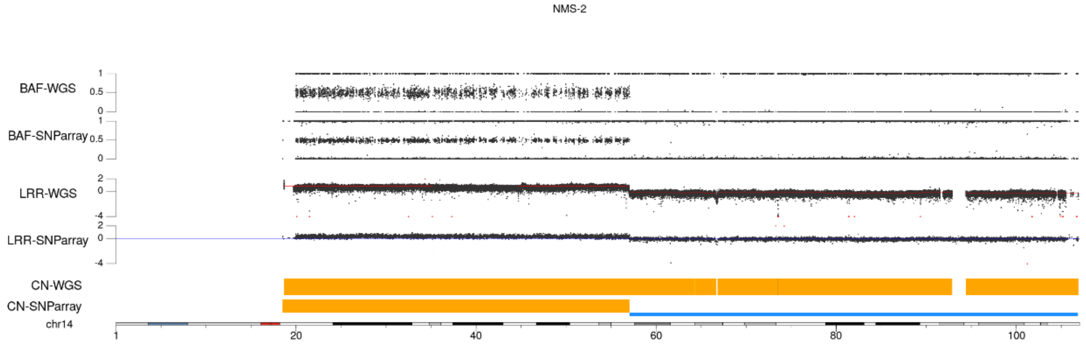
NMS-2
BAF-WGS
BAF-SNParray
LRR-WGS
LRR-SNParray
CN-WGS
CN-SNParray
chr14
1 20 40 60 80 100

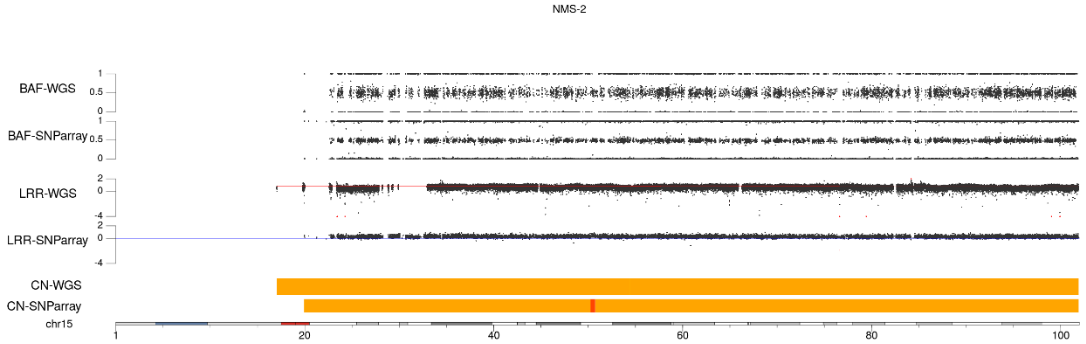
NMS-2
BAF-WGS
BAF-SNParray
LRR-WGS
LRR-SNParray
CN-WGS
CN-SNParray
chr15
1
20
40
60
80
100

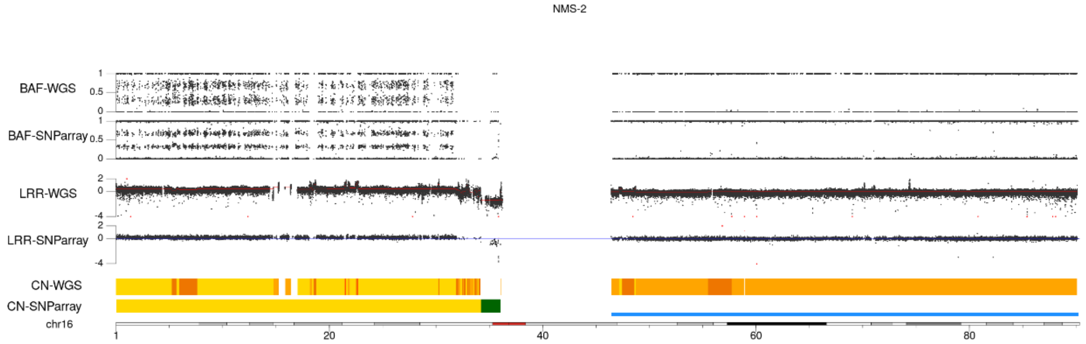
NMS-2
BAF-WGS
BAF-SNParray
LRR-WGS
LRR-SNParray
CN-WGS
CN-SNParray
chr16
1
20
40
60
80

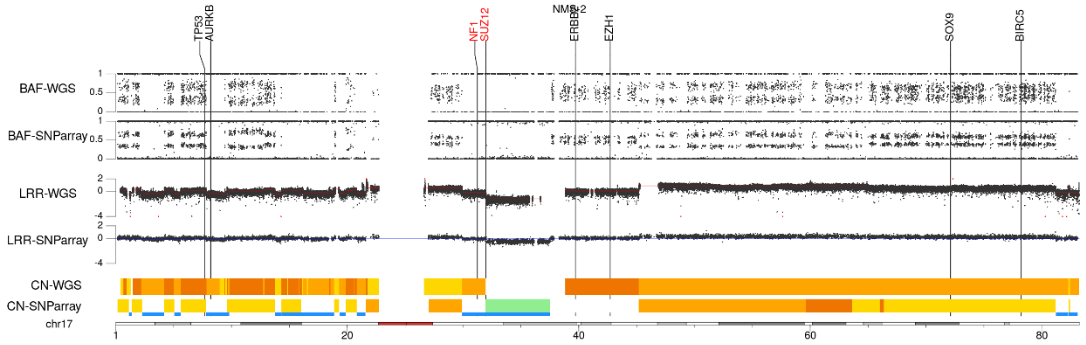
NMS-2
TP53
AURKB
NF1
SUZ12
ERBB2
EZH1
SOX9
BIRC5
BAF-WGS
BAF-SNParray
LRR-WGS
LRR-SNParray
CN-WGS
CN-SNParray
chr17
1
20
40
60
80

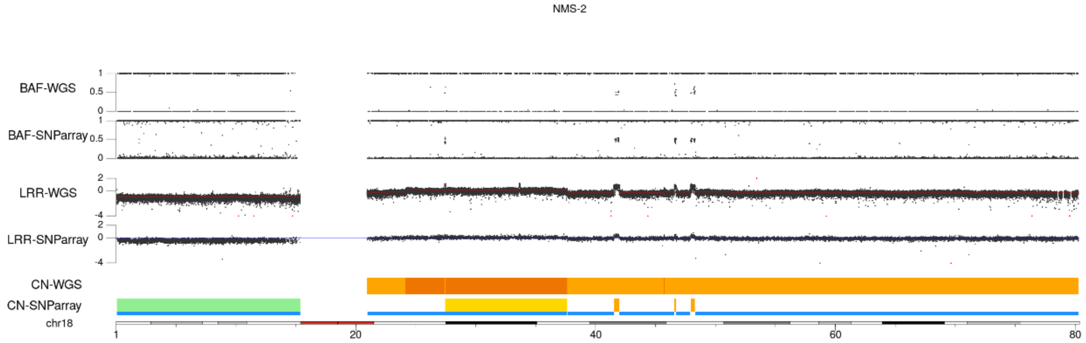
NMS-2
BAF-WGS
BAF-SNParray
LRR-WGS
LRR-SNParray
CN-WGS
CN-SNParray
chr18
1
20
40
60
80

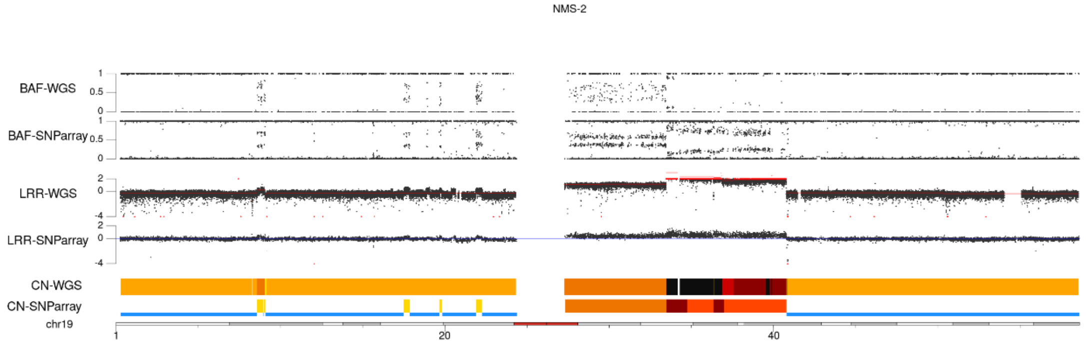
NMS-2
BAF-WGS
BAF-SNParray
LRR-WGS
LRR-SNParray
CN-WGS
CN-SNParray
chr19
1
20
40

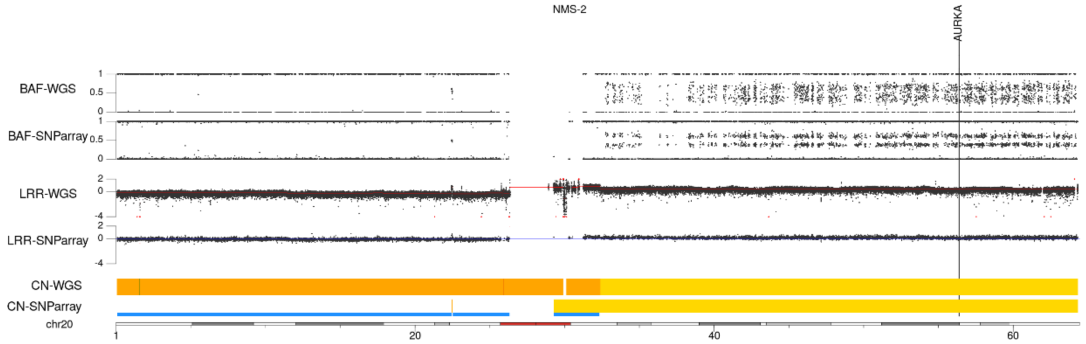
NMS-2
AURKA
BAF-WGS
BAF-SNParray
LRR-WGS
LRR-SNParray
CN-WGS
CN-SNParray
chr20
1
20
40
60

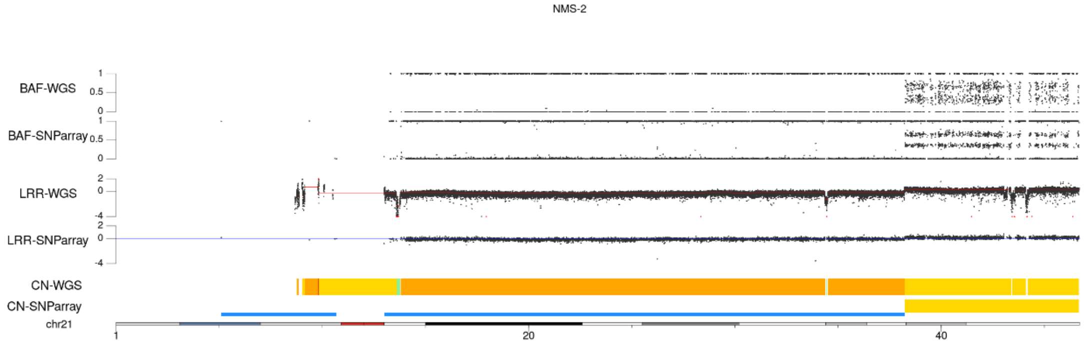
NMS-2
BAF-WGS
BAF-SNParray
LRR-WGS
LRR-SNParray
CN-WGS
CN-SNParray
chr21
1
20
40

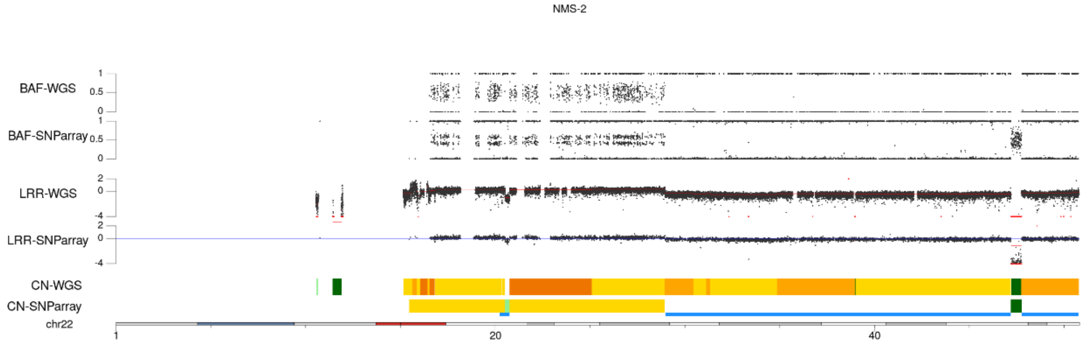
NMS-2
BAF-WGS
BAF-SNParray
LRR-WGS
LRR-SNParray
CN-WGS
CN-SNParray
chr22
1
20
40

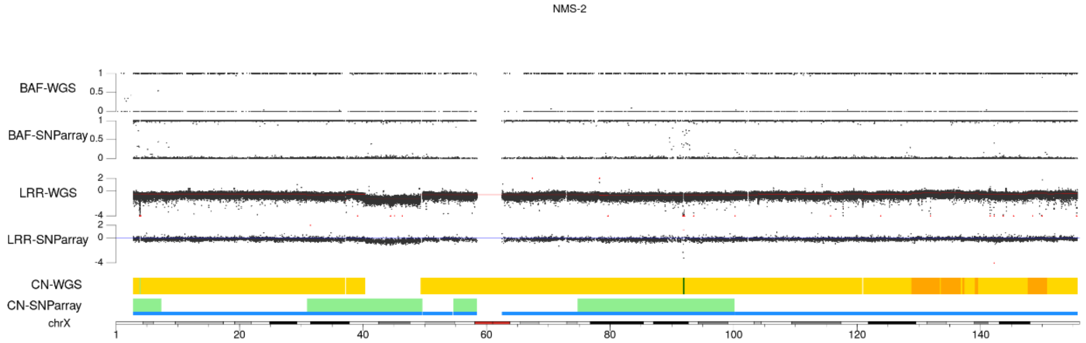
NMS-2
BAF-WGS
BAF-SNParray
LRR-WGS
LRR-SNParray
CN-WGS
CN-SNParray
chrX
1
20
40
60
80
100
120
140

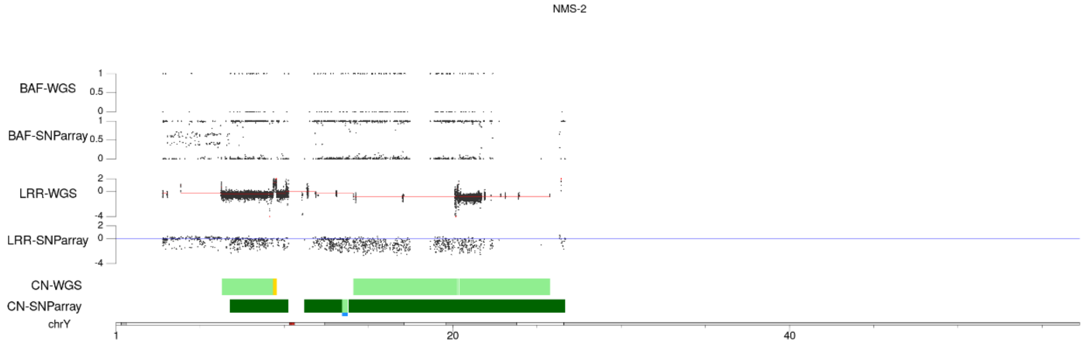
NMS-2
BAF-WGS
BAF-SNParray
LRR-WGS
LRR-SNParray
CN-WGS
CN-SNParray
chrY
1
20
40

STS-26T

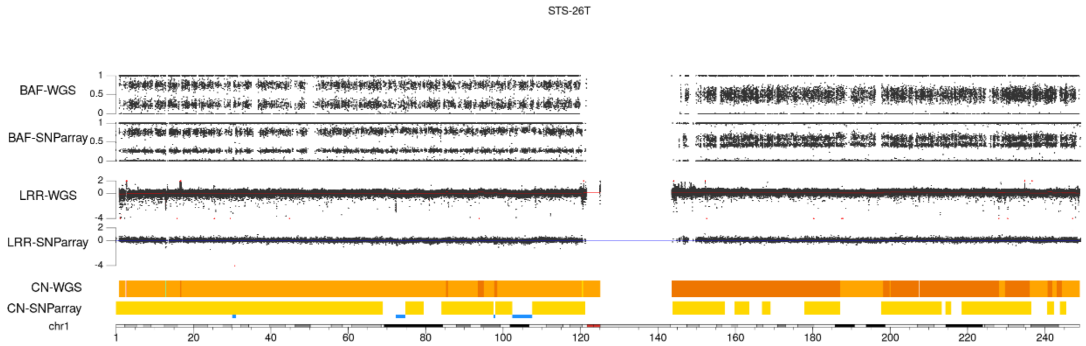
STS-26T
BAF-WGS
BAF-SNParray
LRR-WGS
LRR-SNParray
CN-WGS
CN-SNParray
chr1

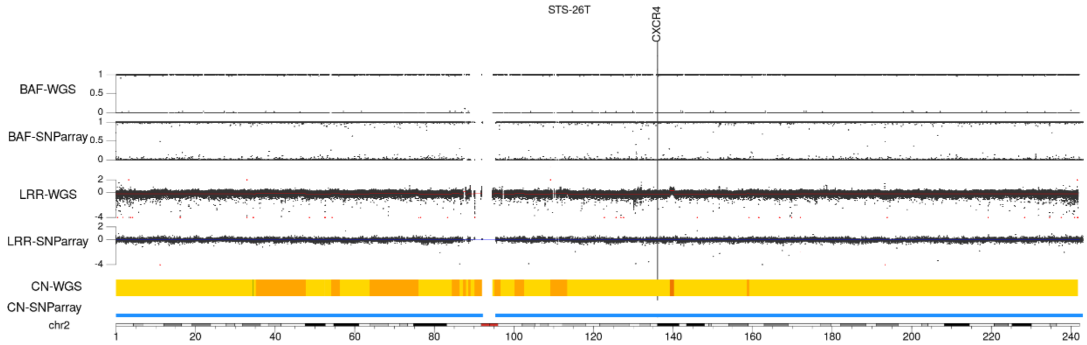
STS-26T
CXCR4
BAF-WGS
BAF-SNParray
LRR-WGS
LRR-SNParray
CN-WGS
CN-SNParray
chr2

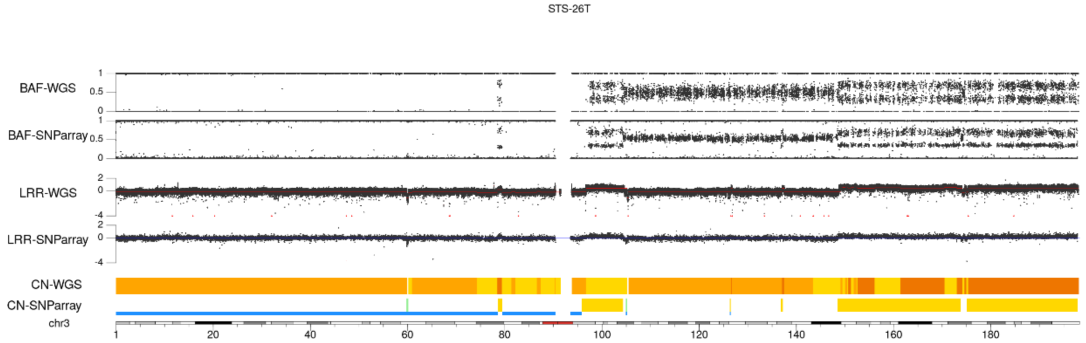
STS-26T
BAF-WGS
BAF-SNParray
LRR-WGS
LRR-SNParray
CN-WGS
CN-SNParray
chr3

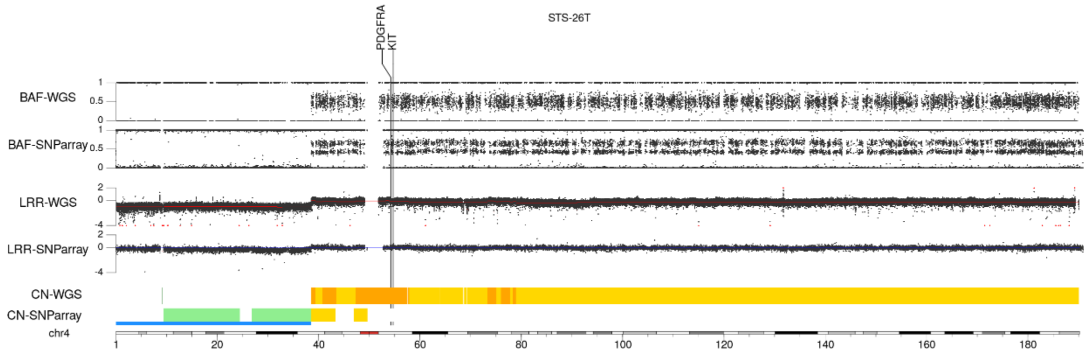
STS-26T
PDGFRA
KIT
BAF-WGS
BAF-SNParray
LRR-WGS
LRR-SNParray
CN-WGS
CN-SNParray
chr4

STS-26T

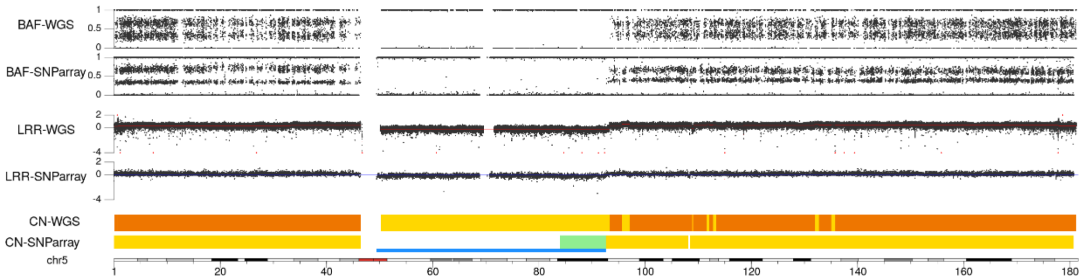

STS-26T

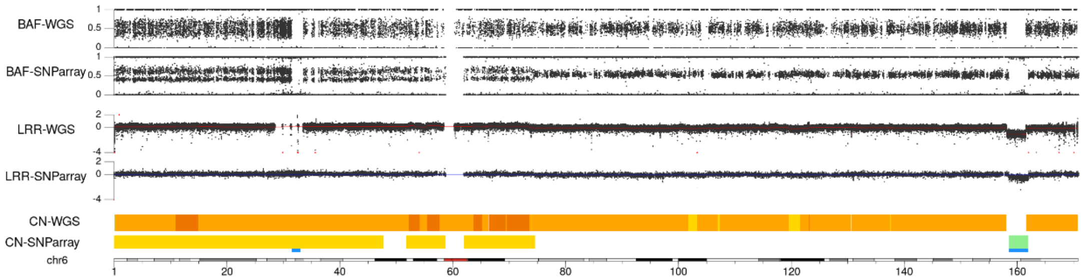

STS-26T

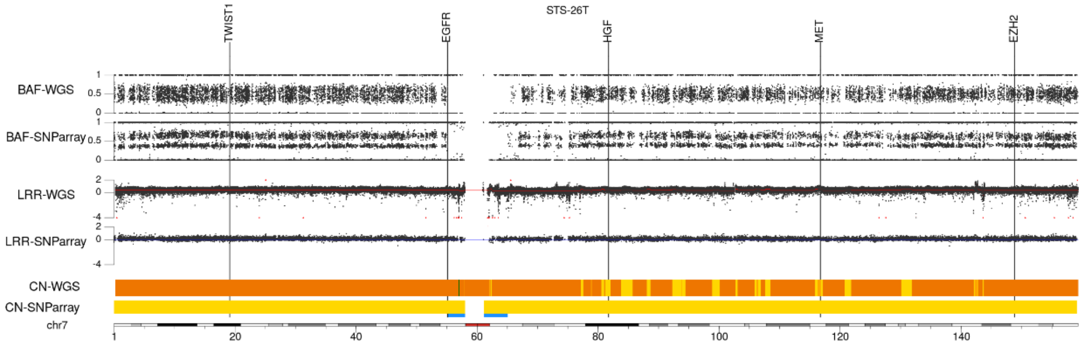

STS-26T

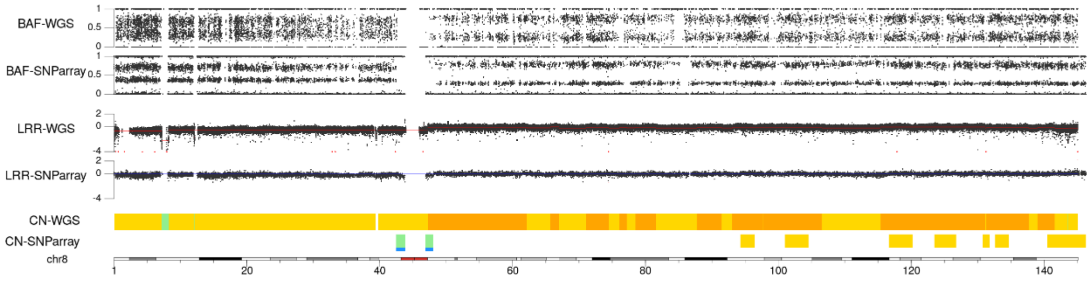

STS-26T

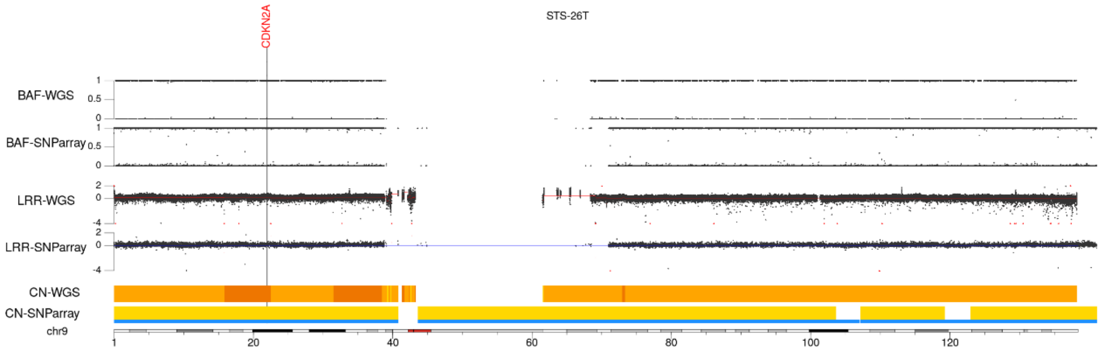

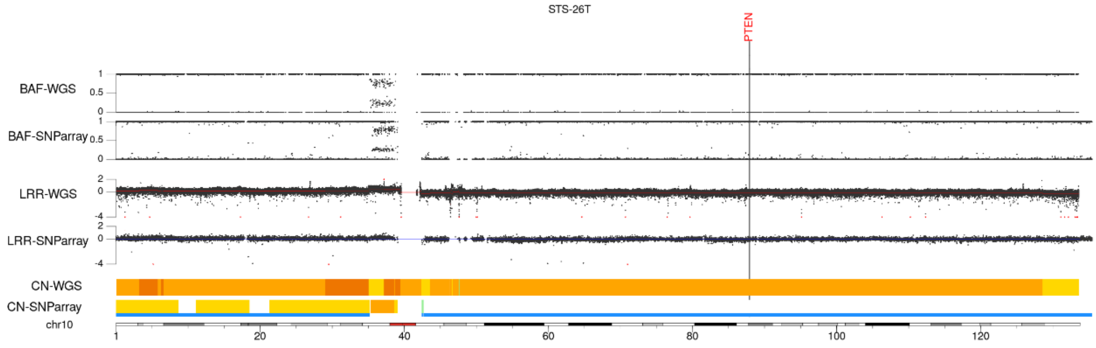
STS-26T
PTEN
BAF-WGS
BAF-SNParray
LRR-WGS
LRR-SNParray
CN-WGS
CN-SNParray
chr10
1
20
40
60
80
100
120

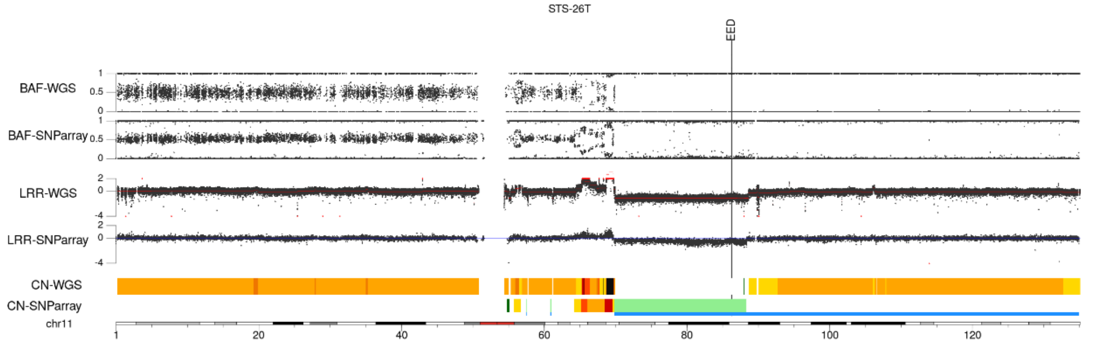
STS-26T
EED
BAF-WGS
BAF-SNParray
LRR-WGS
LRR-SNParray
CN-WGS
CN-SNParray
chr11
1
20
40
60
80
100
120

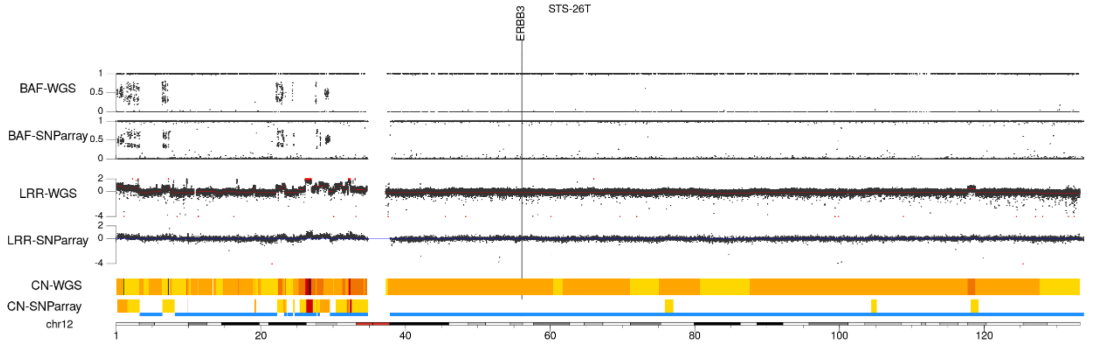
STS-26T
ERBB3
BAF-WGS
BAF-SNParray
LRR-WGS
LRR-SNParray
CN-WGS
CN-SNParray
chr12
1
20
40
60
80
100
120

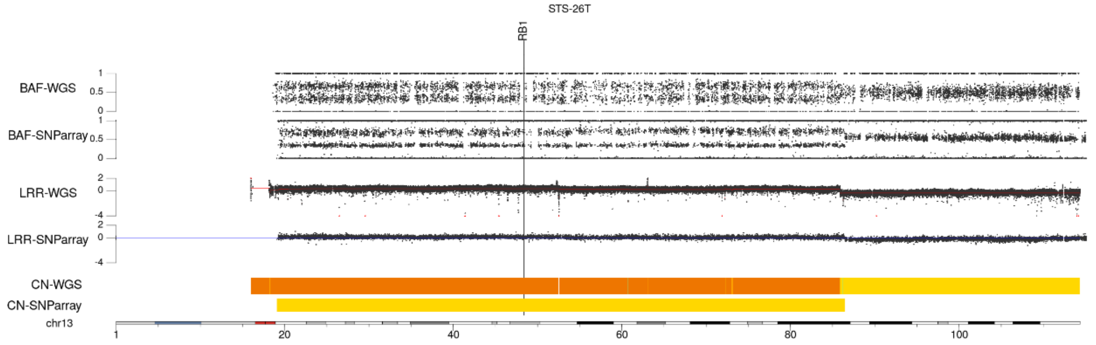
STS-26T
RB1
BAF-WGS
BAF-SNParray
LRR-WGS
LRR-SNParray
CN-WGS
CN-SNParray
chr13
1
20
40
60
80
100

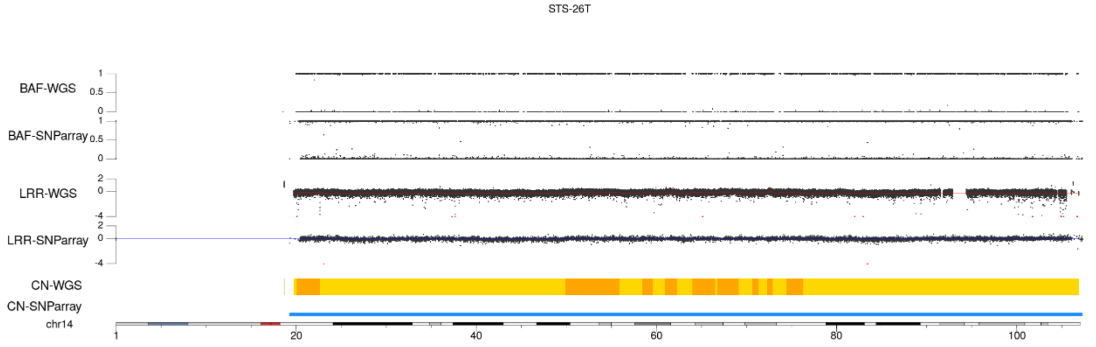
STS-26T
BAF-WGS
BAF-SNParray
LRR-WGS
LRR-SNParray
CN-WGS
CN-SNParray
chr14
1
20
40
60
80
100

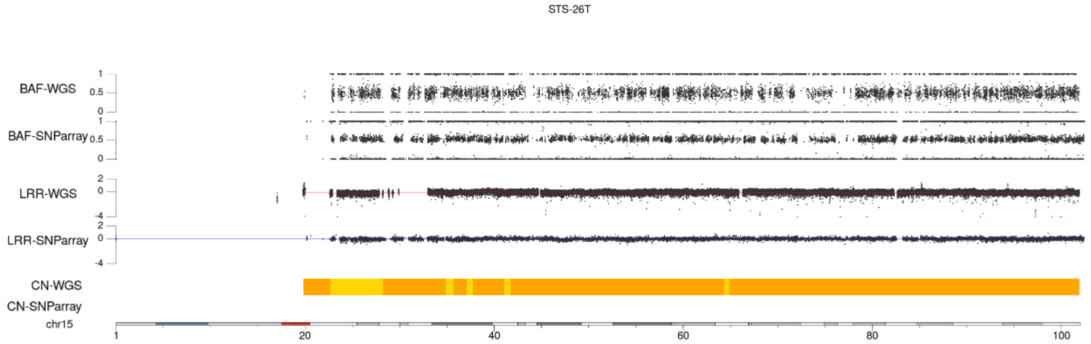
STS-26T
BAF-WGS
BAF-SNParray
LRR-WGS
LRR-SNParray
CN-WGS
CN-SNParray
chr15

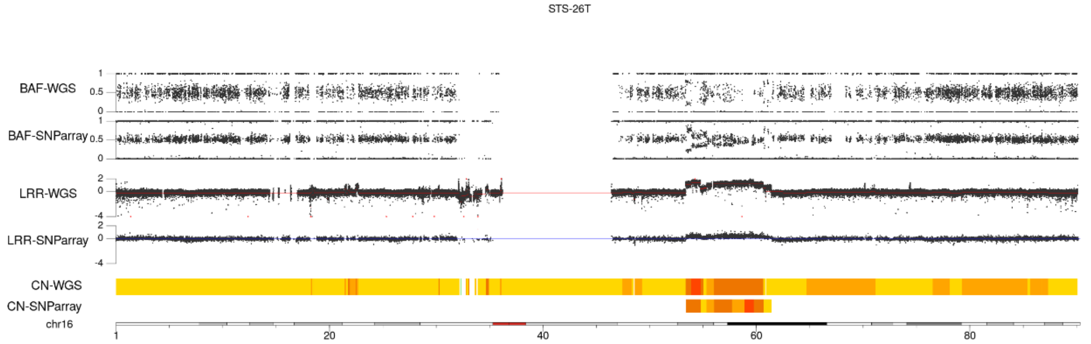
STS-26T
BAF-WGS
BAF-SNParray
LRR-WGS
LRR-SNParray
CN-WGS
CN-SNParray
chr16

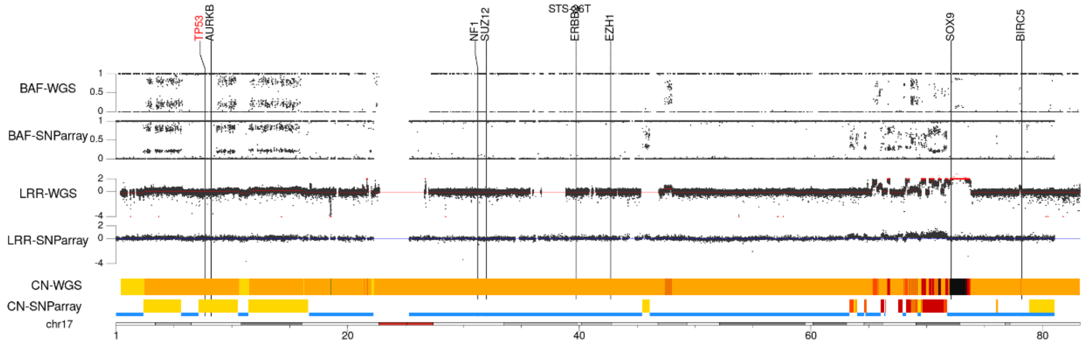
STS-26T
TP53
AURKB
NF1
SUZ12
ERBB2
EZH1
SOX9
BIRC5
BAF-WGS
BAF-SNParray
LRR-WGS
LRR-SNParray
CN-WGS
CN-SNParray
chr17

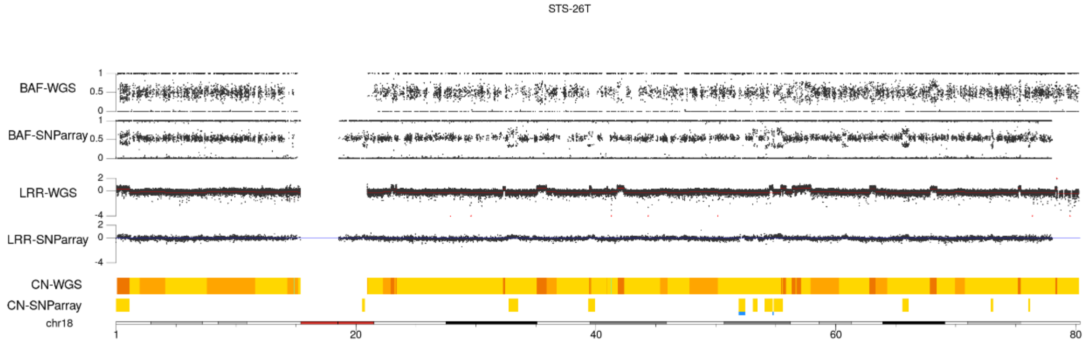
STS-26T
BAF-WGS
BAF-SNParray
LRR-WGS
LRR-SNParray
CN-WGS
CN-SNParray
chr18

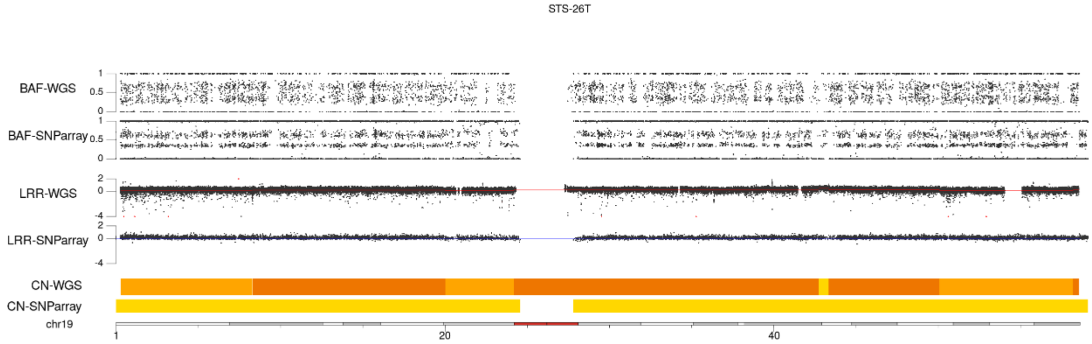
STS-26T
BAF-WGS
BAF-SNParray
LRR-WGS
LRR-SNParray
CN-WGS
CN-SNParray
chr19

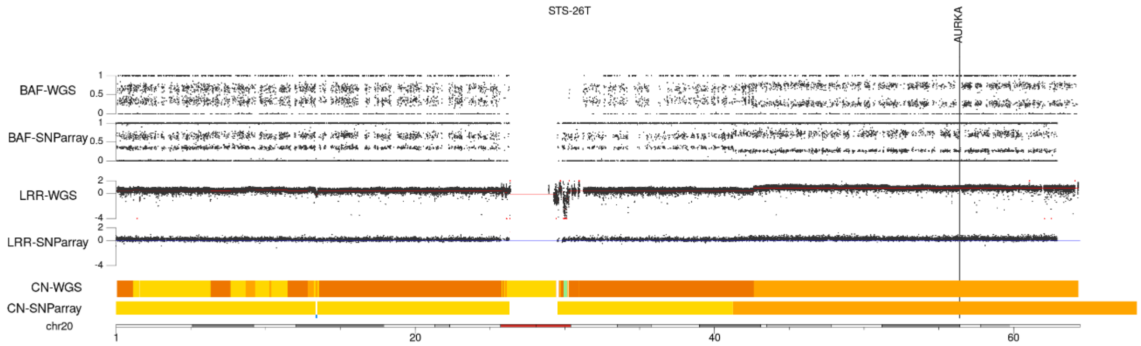
STS-26T
AURKA
BAF-WGS
BAF-SNParray
LRR-WGS
LRR-SNParray
CN-WGS
CN-SNParray
chr20
1
20
40
60

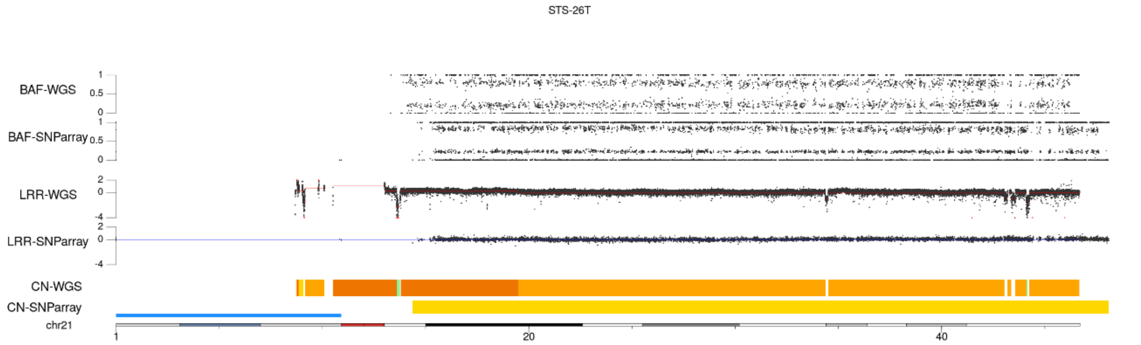
STS-26T
BAF-WGS
BAF-SNParray
LRR-WGS
LRR-SNParray
CN-WGS
CN-SNParray
chr21
1
20
40

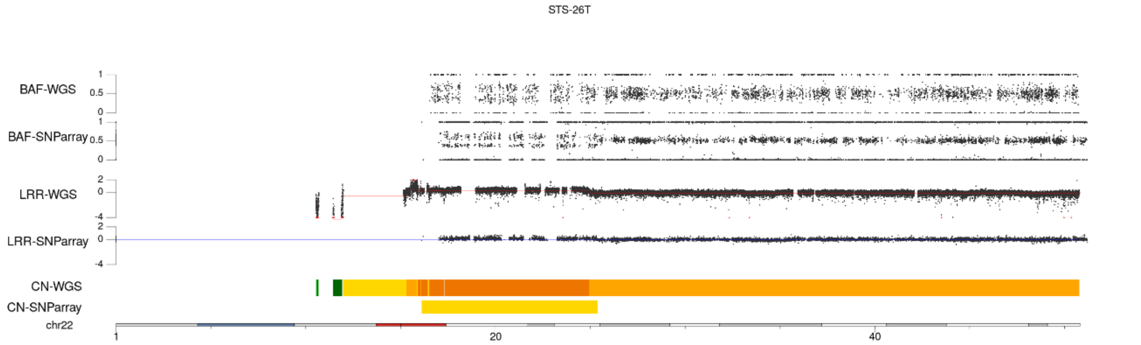
STS-26T
BAF-WGS
BAF-SNParray
LRR-WGS
LRR-SNParray
CN-WGS
CN-SNParray
chr22
1
20
40

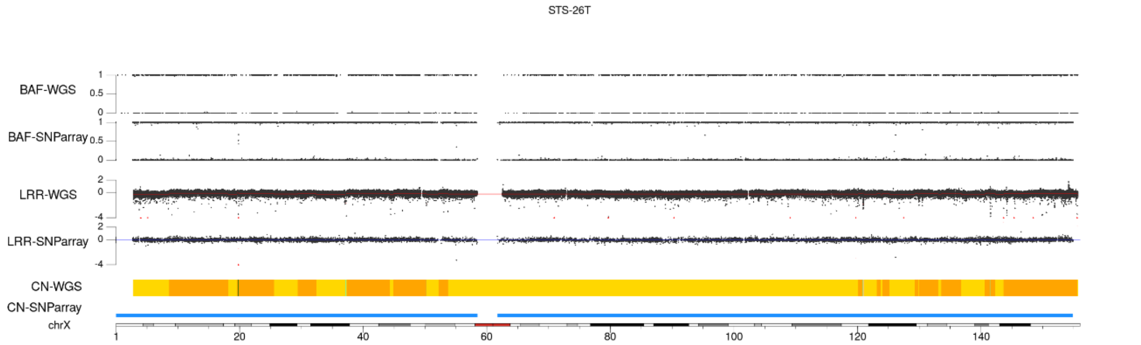
STS-26T
BAF-WGS
BAF-SNParray
LRR-WGS
LRR-SNParray
CN-WGS
CN-SNParray
chrX
1
20
40
60
80
100
120
140

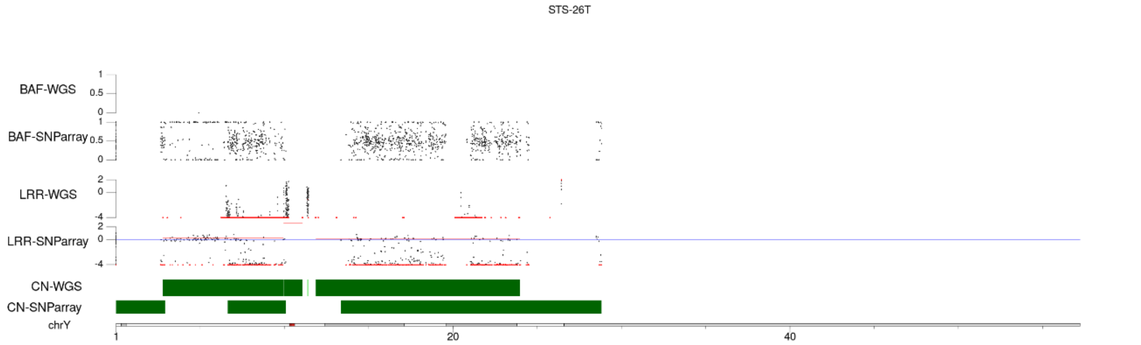
STS-26T
BAF-WGS
BAF-SNParray
LRR-WGS
LRR-SNParray
CN-WGS
CN-SNParray
chrY
1
20
40

HS-Sch-2

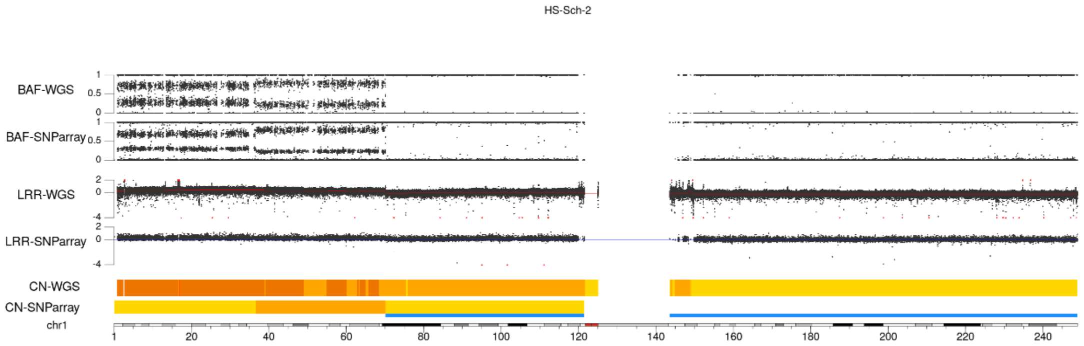
HS-Sch-2
BAF-WGS
BAF-SNParray
LRR-WGS
LRR-SNParray
CN-WGS
CN-SNParray
chr1
1
20
40
60
80
100
120
140
160
180
200
220
240

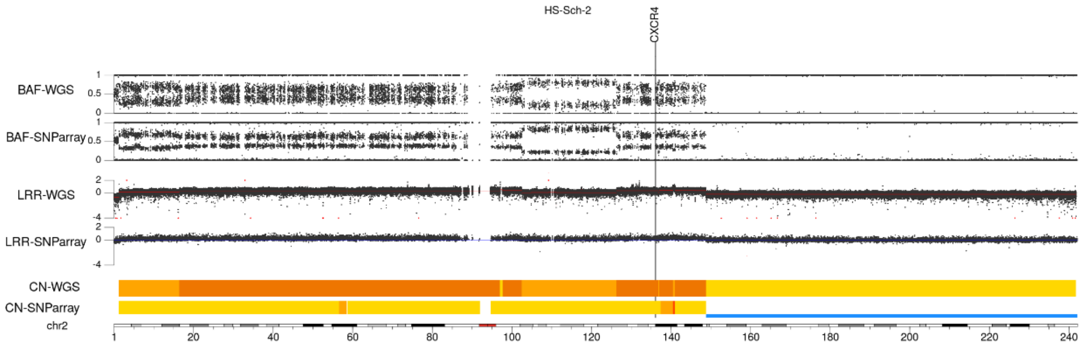
HS-Sch-2
CXCR4
BAF-WGS
BAF-SNParray
LRR-WGS
LRR-SNParray
CN-WGS
CN-SNParray
chr2
1
20
40
60
80
100
120
140
160
180
200
220
240

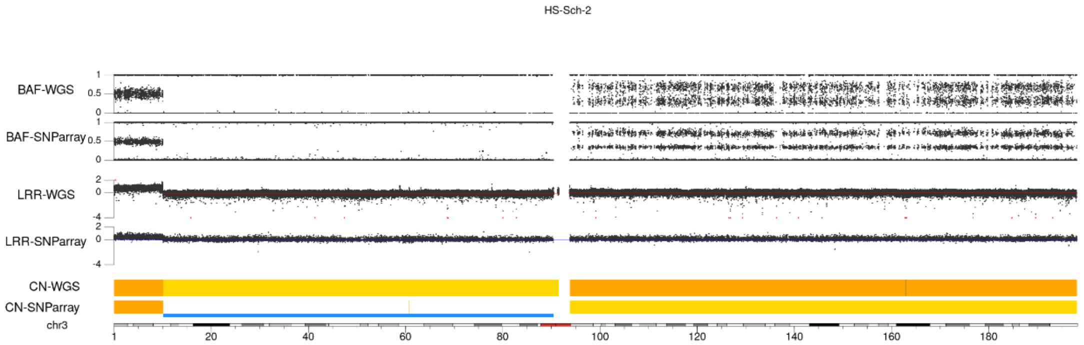
HS-Sch-2
BAF-WGS
BAF-SNParray
LRR-WGS
LRR-SNParray
CN-WGS
CN-SNParray
chr3
1
20
40
60
80
100
120
140
160
180

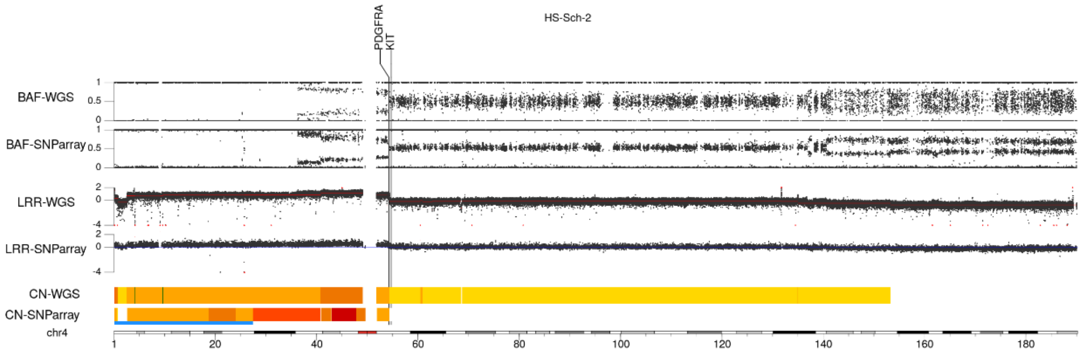
HS-Sch-2
PDGFRA
KIT
BAF-WGS
BAF-SNParray
LRR-WGS
LRR-SNParray
CN-WGS
CN-SNParray
chr4
1
20
40
60
80
100
120
140
160
180

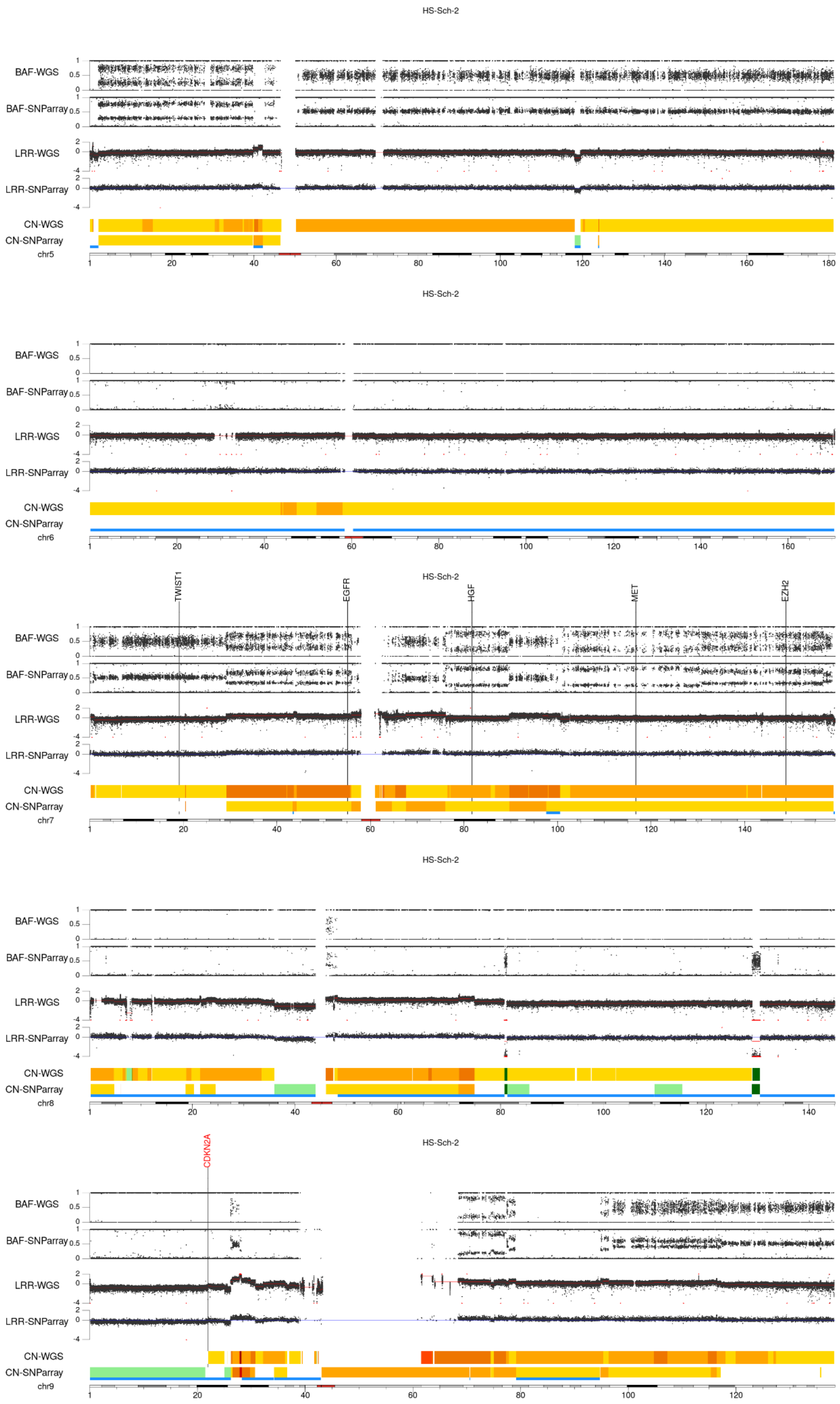
HS-Sch-2
BAF-WGS
BAF-SNParray
LRR-WGS
LRR-SNParray
CN-WGS
CN-SNParray
chr5
1 20 40 60 80 100 120 140 160 180
HS-Sch-2
BAF-WGS
BAF-SNParray
LRR-WGS
LRR-SNParray
CN-WGS
CN-SNParray
chr6
1 20 40 60 80 100 120 140 160
HS-Sch-2
TWIST1
EGFR
HGF
MET
EZH2
BAF-WGS
BAF-SNParray
LRR-WGS
LRR-SNParray
CN-WGS
CN-SNParray
chr7
1 20 40 60 80 100 120 140
HS-Sch-2
BAF-WGS
BAF-SNParray
LRR-WGS
LRR-SNParray
CN-WGS
CN-SNParray
chr8
1 20 40 60 80 100 120 140
HS-Sch-2
CDKN2A
BAF-WGS
BAF-SNParray
LRR-WGS
LRR-SNParray
CN-WGS
CN-SNParray
chr9
1 20 40 60 80 100 120

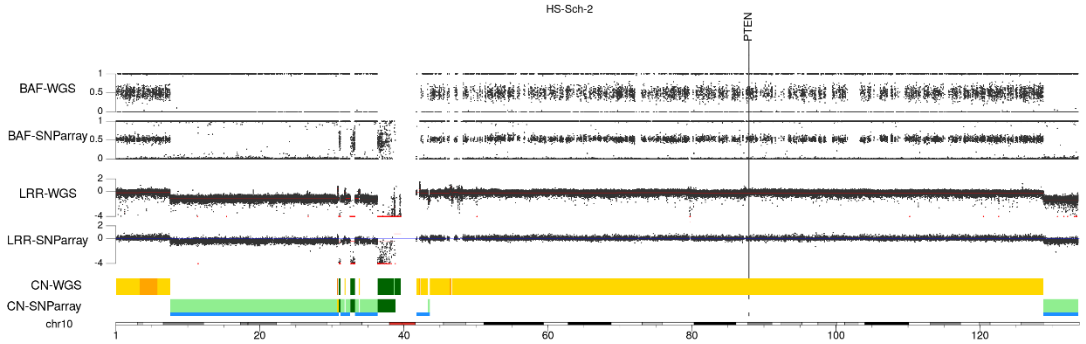
HS-Sch-2
PTEN
BAF-WGS
BAF-SNParray
LRR-WGS
LRR-SNParray
CN-WGS
CN-SNParray
chr10
1 20 40 60 80 100 120

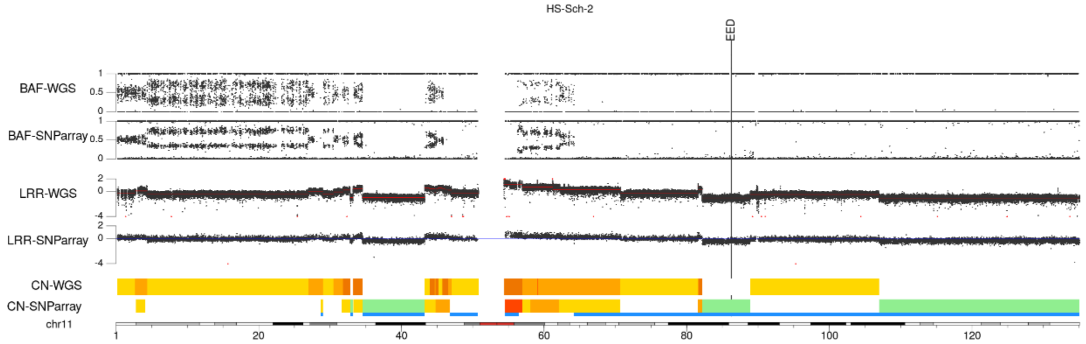
HS-Sch-2
EED
BAF-WGS
BAF-SNParray
LRR-WGS
LRR-SNParray
CN-WGS
CN-SNParray
chr11
1 20 40 60 80 100 120

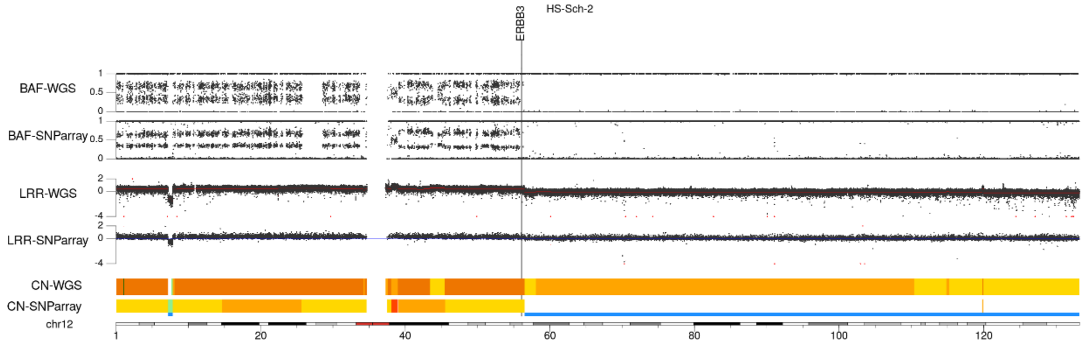
HS-Sch-2
ERBB3
BAF-WGS
BAF-SNParray
LRR-WGS
LRR-SNParray
CN-WGS
CN-SNParray
chr12
1 20 40 60 80 100 120

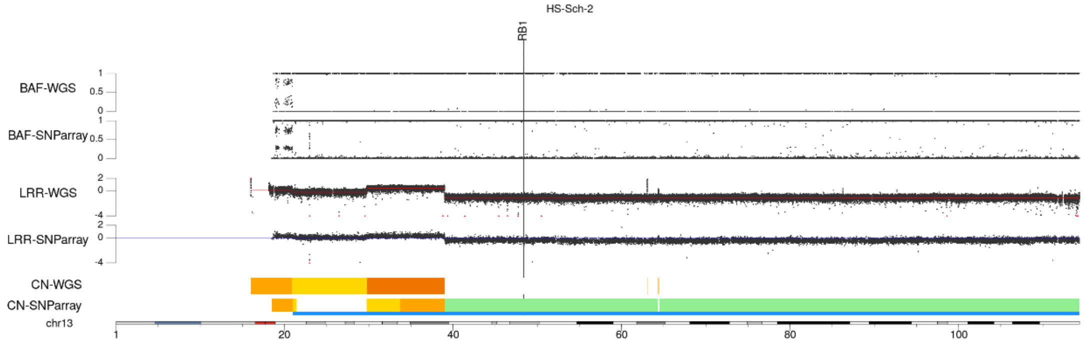
HS-Sch-2
RB1
BAF-WGS
BAF-SNParray
LRR-WGS
LRR-SNParray
CN-WGS
CN-SNParray
chr13
1 20 40 60 80 100

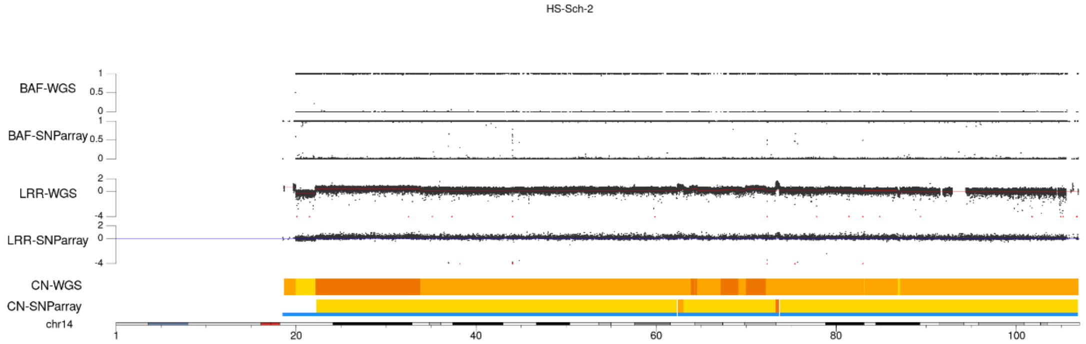
HS-Sch-2
BAF-WGS
BAF-SNParray
LRR-WGS
LRR-SNParray
CN-WGS
CN-SNParray
chr14
1 20 40 60 80 100

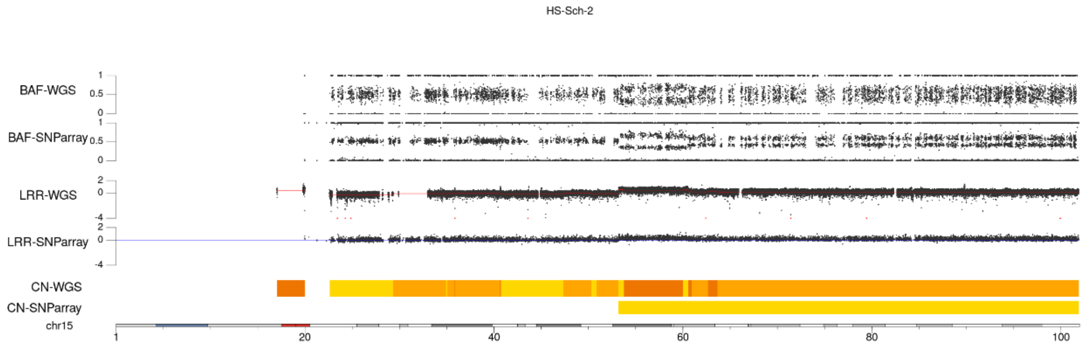
HS-Sch-2
BAF-WGS
BAF-SNParray
LRR-WGS
LRR-SNParray
CN-WGS
CN-SNParray
chr15
1
20
40
60
80
100

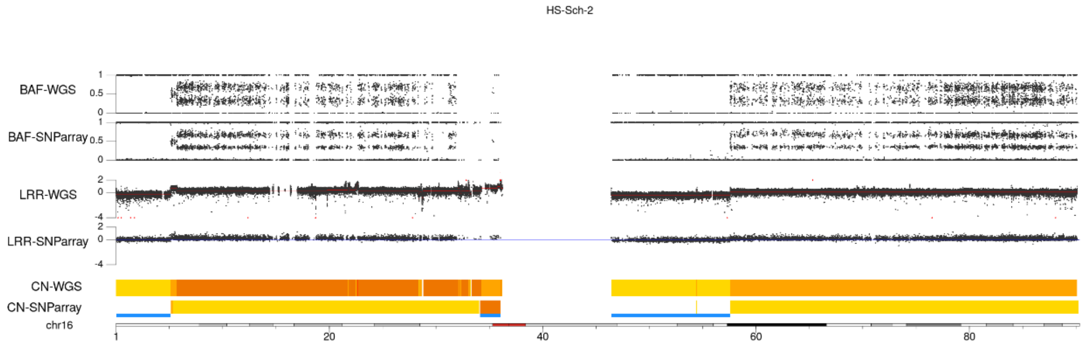
HS-Sch-2
BAF-WGS
BAF-SNParray
LRR-WGS
LRR-SNParray
CN-WGS
CN-SNParray
chr16
1
20
40
60
80

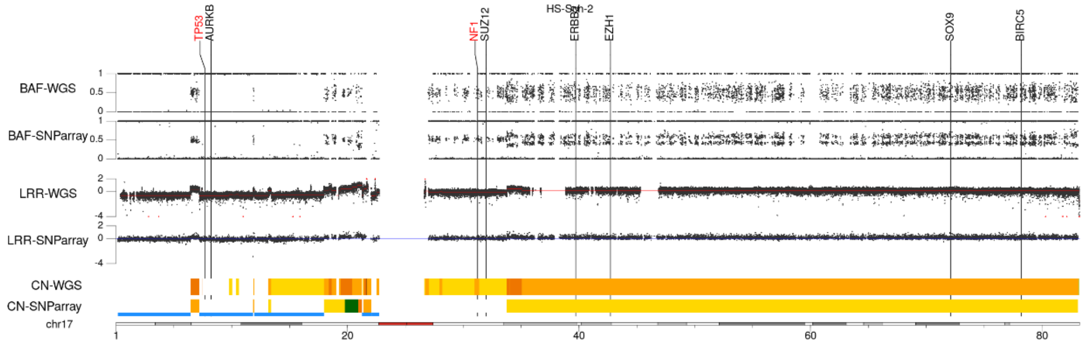
HS-Sch-2
TP53
AURKB
NF1
SUZ12
ERBB2
EZH1
SOX9
BIRC5
BAF-WGS
BAF-SNParray
LRR-WGS
LRR-SNParray
CN-WGS
CN-SNParray
chr17
1
20
40
60
80

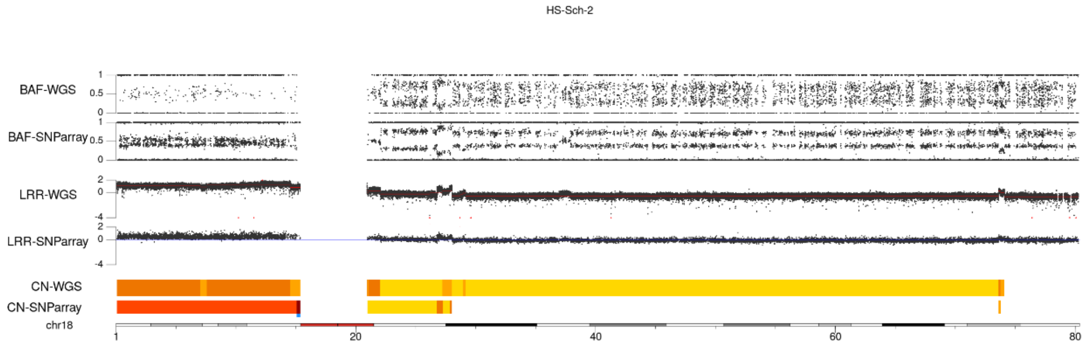
HS-Sch-2
BAF-WGS
BAF-SNParray
LRR-WGS
LRR-SNParray
CN-WGS
CN-SNParray
chr18
1
20
40
60
80

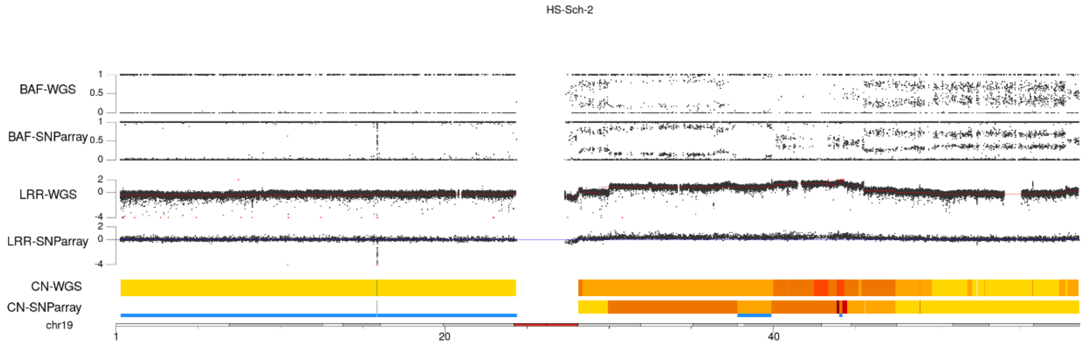
HS-Sch-2
BAF-WGS
BAF-SNParray
LRR-WGS
LRR-SNParray
CN-WGS
CN-SNParray
chr19
1
20
40

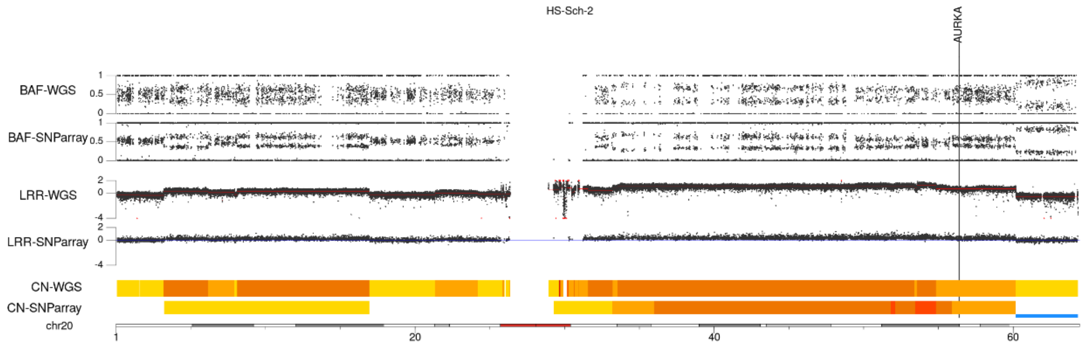
HS-Sch-2
AURKA
BAF-WGS
BAF-SNParray
LRR-WGS
LRR-SNParray
CN-WGS
CN-SNParray
chr20
1
20
40
60

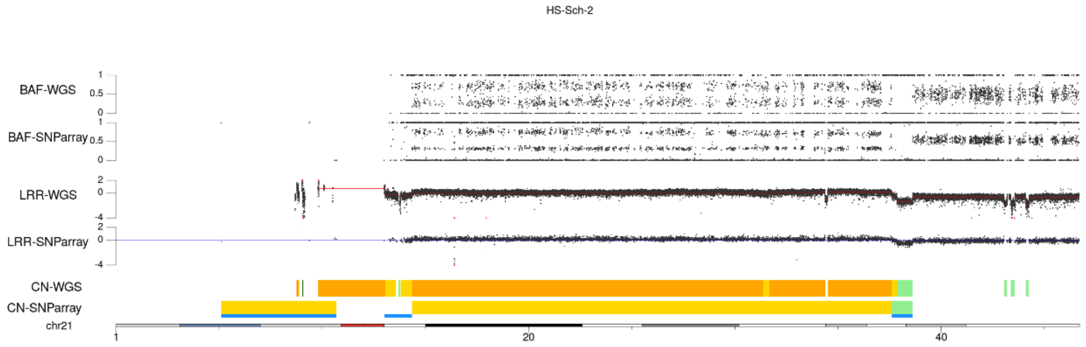
HS-Sch-2
BAF-WGS
BAF-SNParray
LRR-WGS
LRR-SNParray
CN-WGS
CN-SNParray
chr21
1
20
40

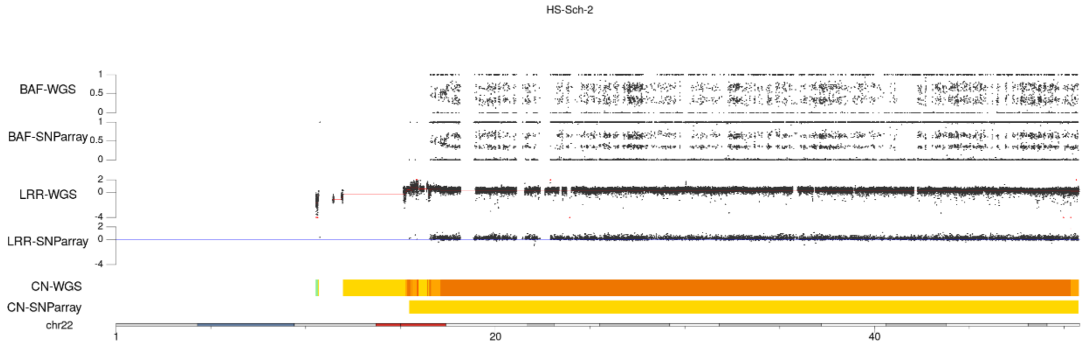
HS-Sch-2
BAF-WGS
BAF-SNParray
LRR-WGS
LRR-SNParray
CN-WGS
CN-SNParray
chr22
1
20
40

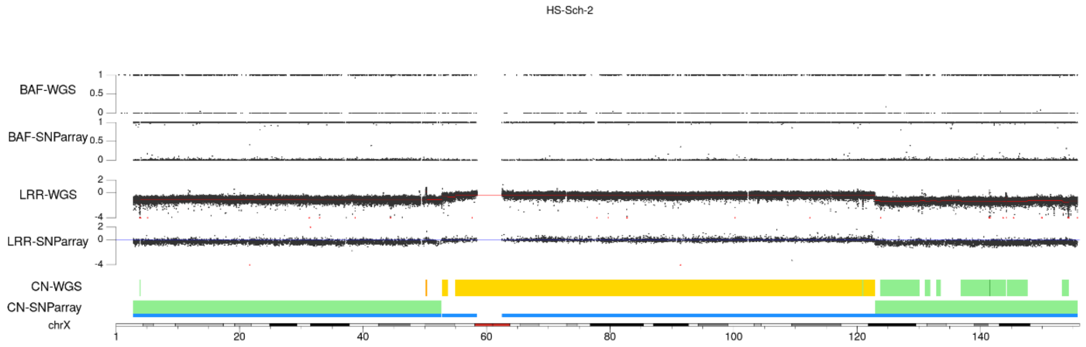
HS-Sch-2
BAF-WGS
BAF-SNParray
LRR-WGS
LRR-SNParray
CN-WGS
CN-SNParray
chrX
1
20
40
60
80
100
120
140

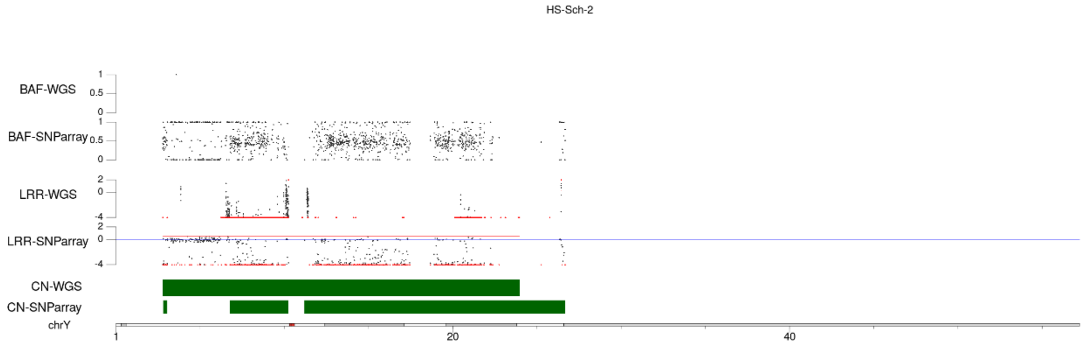
HS-Sch-2
BAF-WGS
BAF-SNParray
LRR-WGS
LRR-SNParray
CN-WGS
CN-SNParray
chrY
1
20
40

HS-PSS

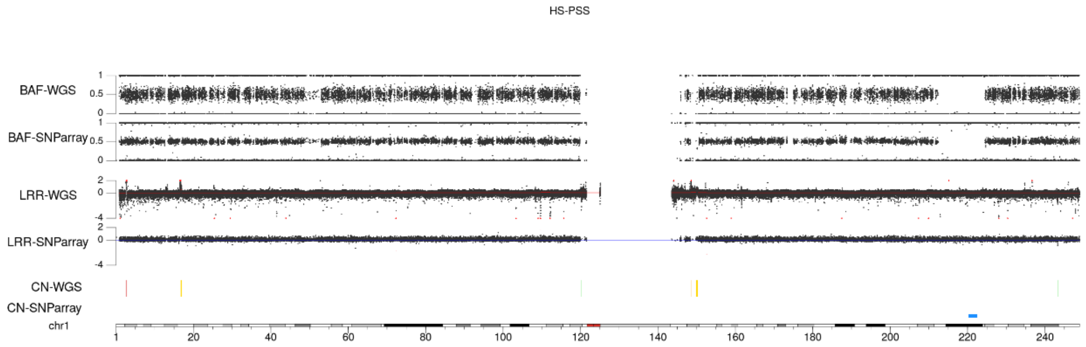
HS-PSS
BAF-WGS
BAF-SNParray
LRR-WGS
LRR-SNParray
CN-WGS
CN-SNParray
chr1
1 20 40 60 80 100 120 140 160 180 200 220 240

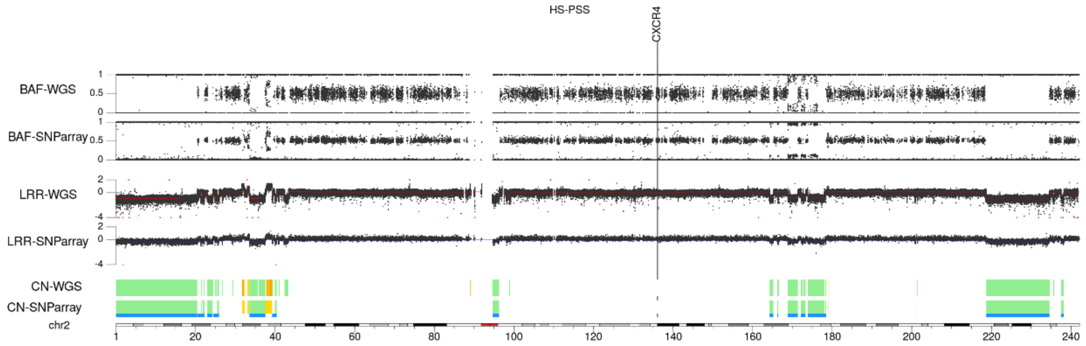
HS-PSS
CXCR4
BAF-WGS
BAF-SNParray
LRR-WGS
LRR-SNParray
CN-WGS
CN-SNParray
chr2
1 20 40 60 80 100 120 140 160 180 200 220 240

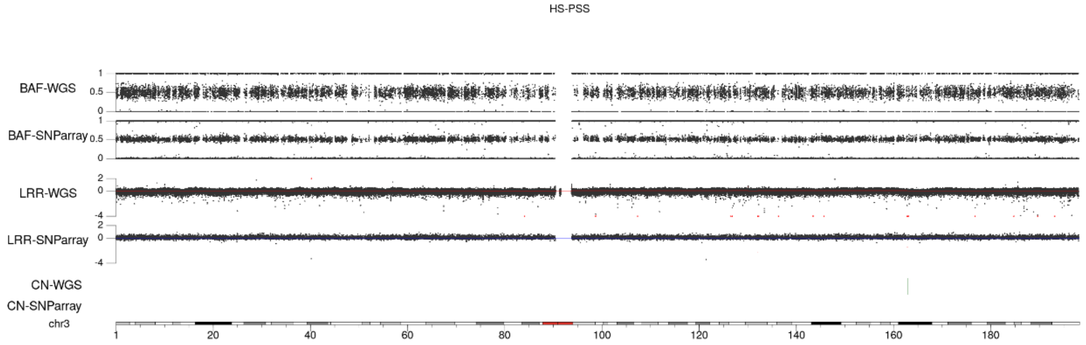
HS-PSS
BAF-WGS
BAF-SNParray
LRR-WGS
LRR-SNParray
CN-WGS
CN-SNParray
chr3
1 20 40 60 80 100 120 140 160 180

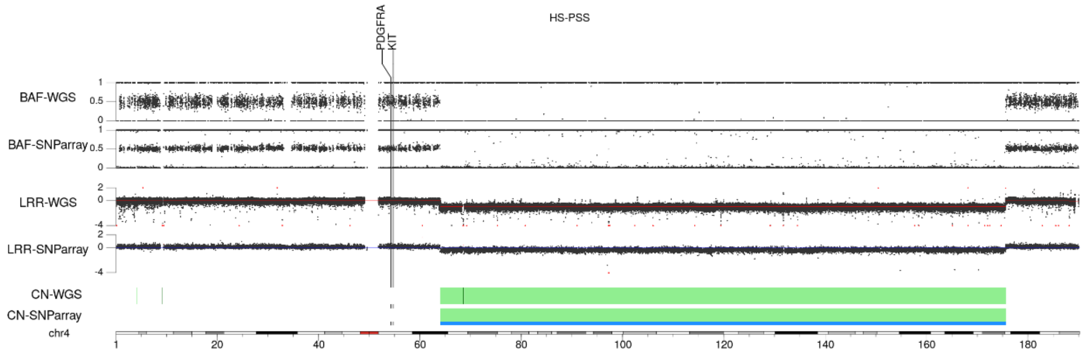
HS-PSS
PDGFRA
KIT
BAF-WGS
BAF-SNParray
LRR-WGS
LRR-SNParray
CN-WGS
CN-SNParray
chr4
1 20 40 60 80 100 120 140 160 180

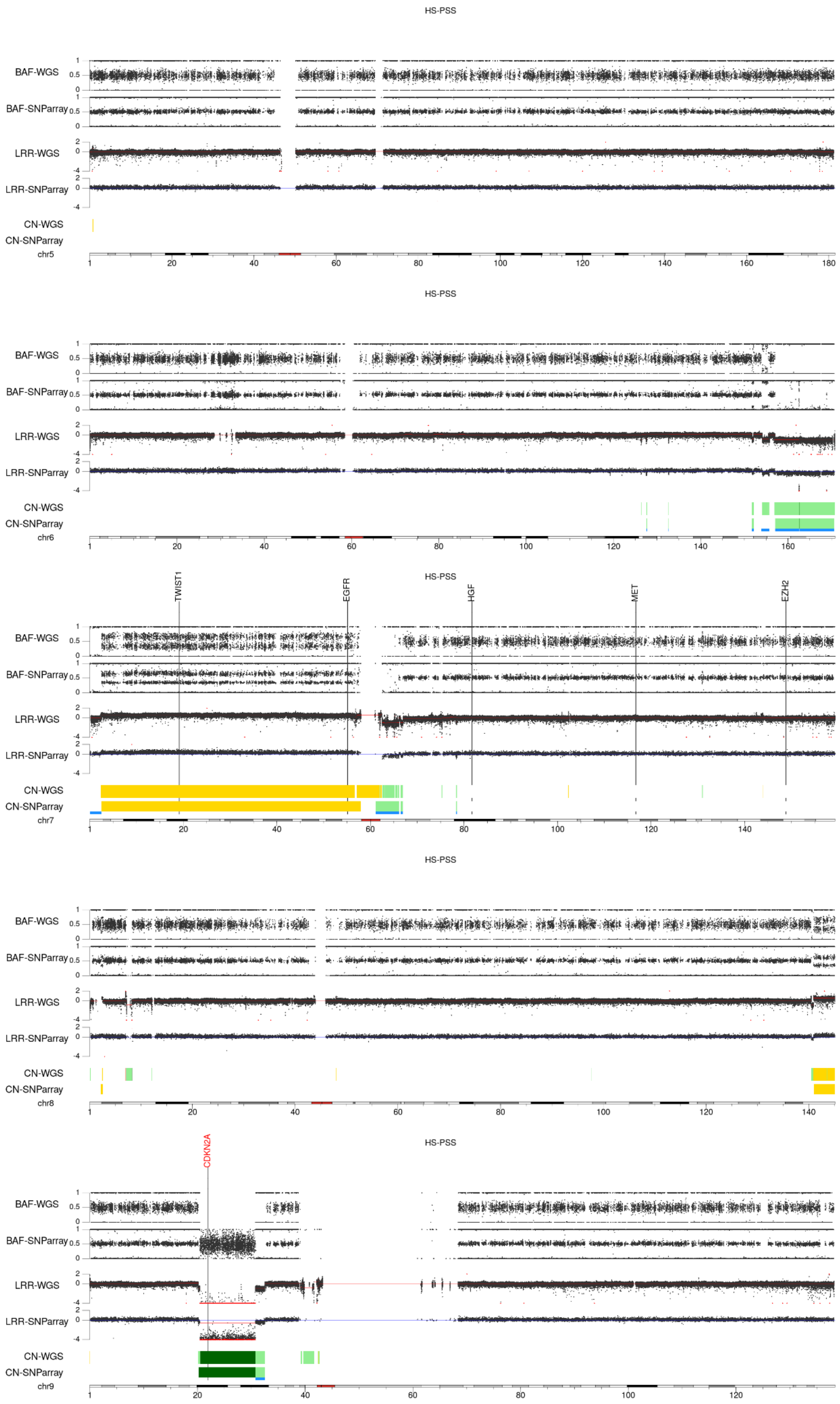
HS-PSS
BAF-WGS
BAF-SNParray
LRR-WGS
LRR-SNParray
CN-WGS
CN-SNParray
chr5
HS-PSS
chr6
HS-PSS
TWIST1
EGFR
HGF
MET
EZH2
chr7
HS-PSS
chr8
HS-PSS
CDKN2A
chr9

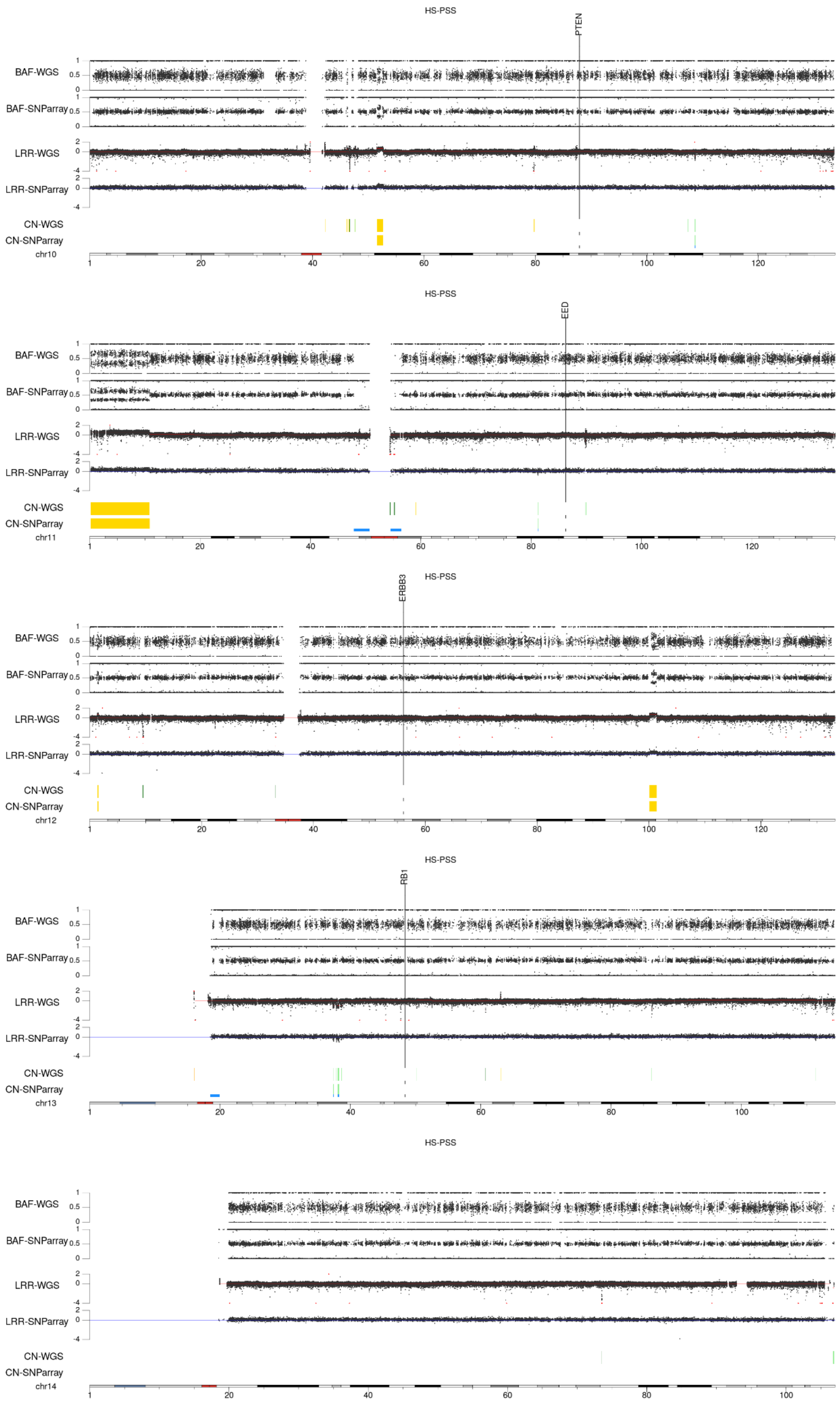
HS-PSS
PTEN
BAF-WGS
BAF-SNParray
LRR-WGS
LRR-SNParray
CN-WGS
CN-SNParray
chr10
HS-PSS
EED
BAF-WGS
BAF-SNParray
LRR-WGS
LRR-SNParray
CN-WGS
CN-SNParray
chr11
HS-PSS
ERBB3
BAF-WGS
BAF-SNParray
LRR-WGS
LRR-SNParray
CN-WGS
CN-SNParray
chr12
HS-PSS
RB1
BAF-WGS
BAF-SNParray
LRR-WGS
LRR-SNParray
CN-WGS
CN-SNParray
chr13
HS-PSS
BAF-WGS
BAF-SNParray
LRR-WGS
LRR-SNParray
CN-WGS
CN-SNParray
chr14

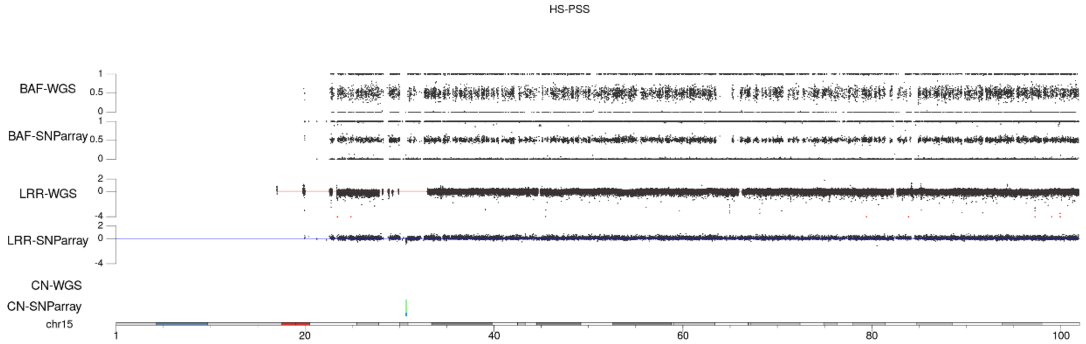
HS-PSS
BAF-WGS
BAF-SNParray
LRR-WGS
LRR-SNParray
CN-WGS
CN-SNParray
chr15
1
20
40
60
80
100

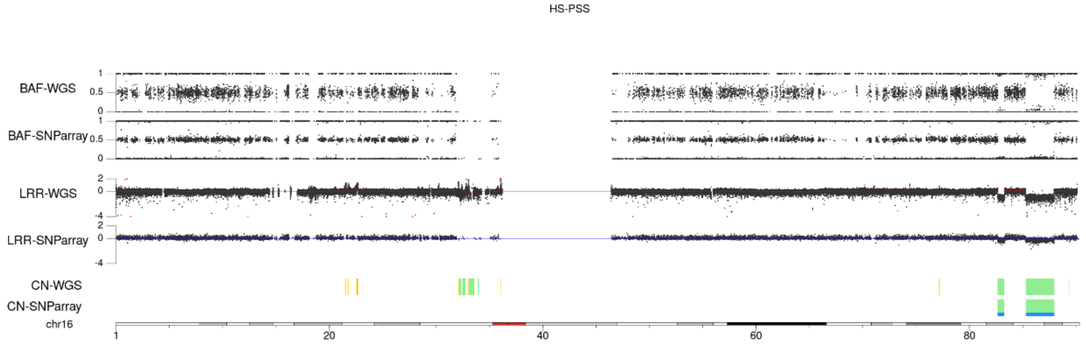
HS-PSS
BAF-WGS
BAF-SNParray
LRR-WGS
LRR-SNParray
CN-WGS
CN-SNParray
chr16
1
20
40
60
80

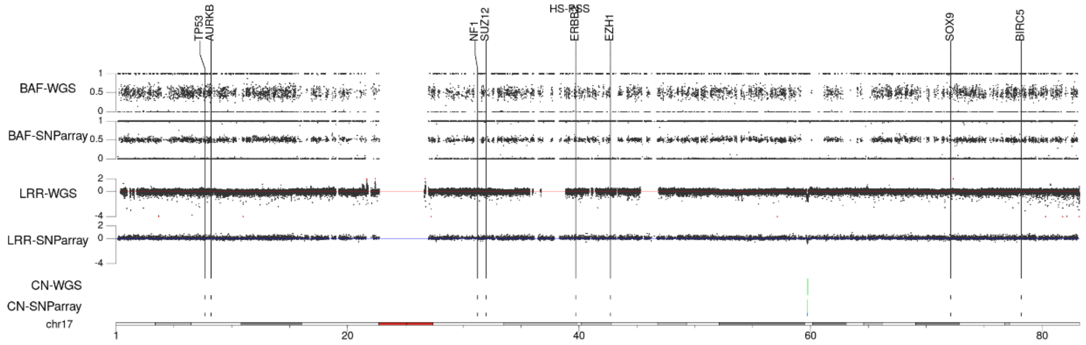
HS-PSS
TP53
AURKB
NF1
SUZ12
ERBB2
EZH1
SOX9
BIRC5
BAF-WGS
BAF-SNParray
LRR-WGS
LRR-SNParray
CN-WGS
CN-SNParray
chr17
1
20
40
60
80

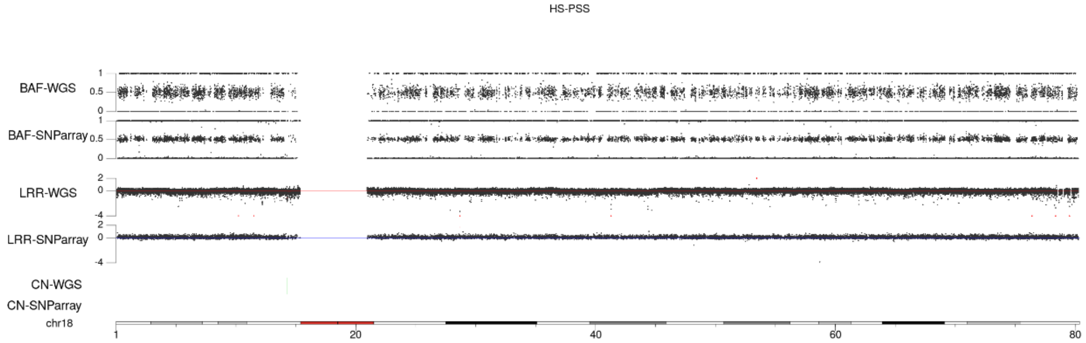
HS-PSS
BAF-WGS
BAF-SNParray
LRR-WGS
LRR-SNParray
CN-WGS
CN-SNParray
chr18
1
20
40
60
80

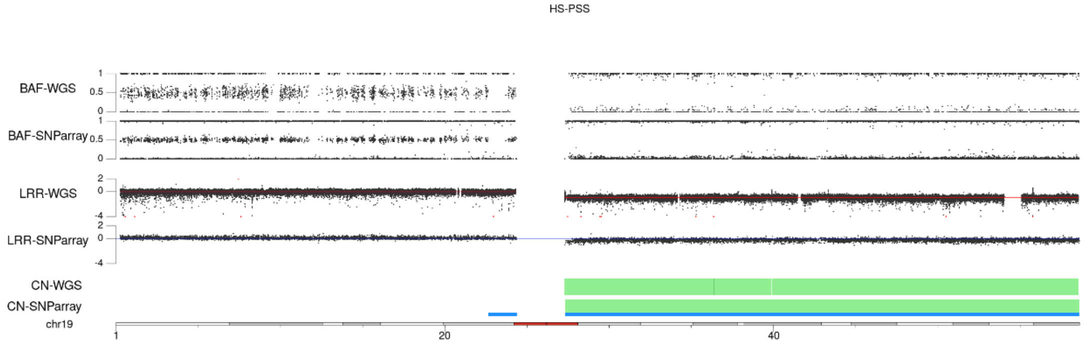
HS-PSS
BAF-WGS
BAF-SNParray
LRR-WGS
LRR-SNParray
CN-WGS
CN-SNParray
chr19
1
20
40

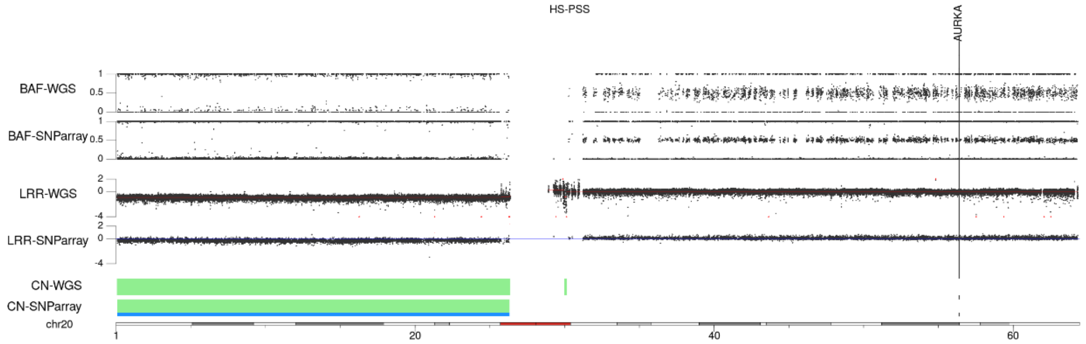
HS-PSS
AURKA
BAF-WGS
BAF-SNParray
LRR-WGS
LRR-SNParray
CN-WGS
CN-SNParray
chr20
1
20
40
60

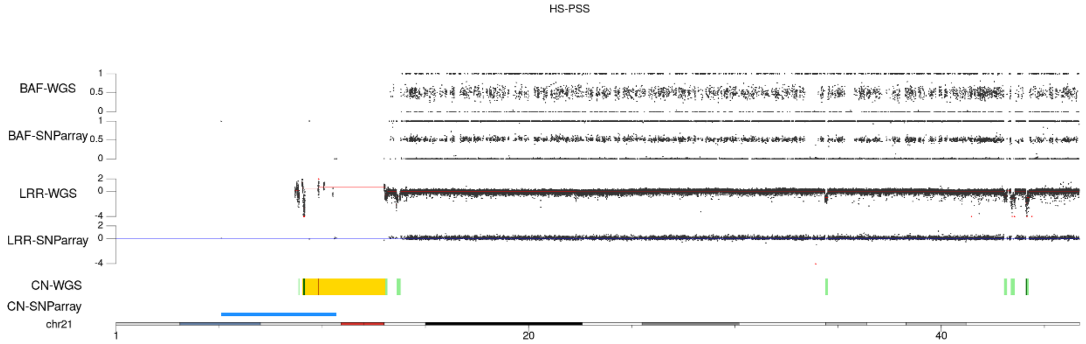
HS-PSS
BAF-WGS
BAF-SNParray
LRR-WGS
LRR-SNParray
CN-WGS
CN-SNParray
chr21
1
20
40

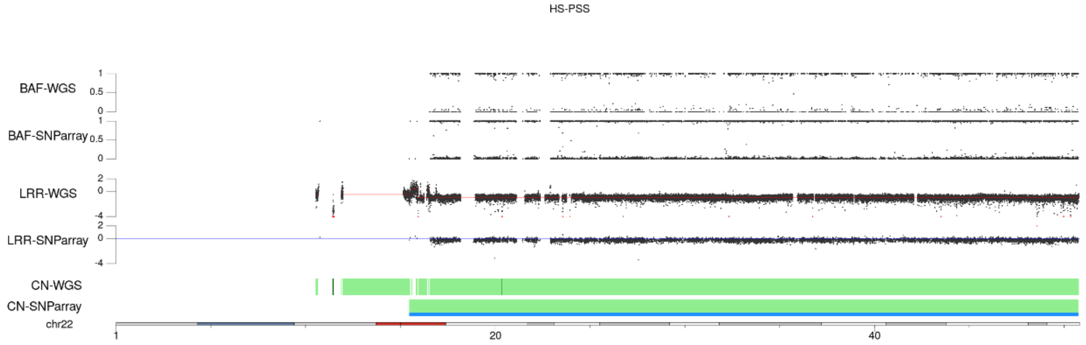
HS-PSS
BAF-WGS
BAF-SNParray
LRR-WGS
LRR-SNParray
CN-WGS
CN-SNParray
chr22
1
20
40

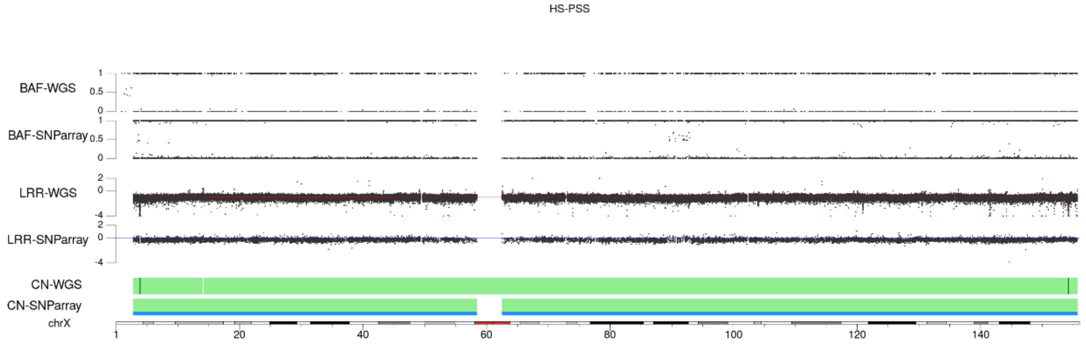
HS-PSS
BAF-WGS
BAF-SNParray
LRR-WGS
LRR-SNParray
CN-WGS
CN-SNParray
chrX
1
20
40
60
80
100
120
140

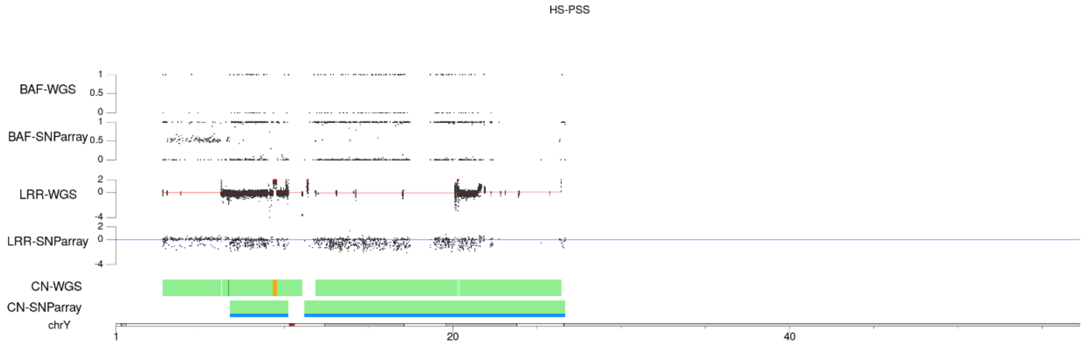
HS-PSS
BAF-WGS
BAF-SNParray
LRR-WGS
LRR-SNParray
CN-WGS
CN-SNParray
chrY
1
20
40

Data S5:
Co-evolution examples by SNP-array profile analysis.

Related to Table1

By comparing the genomic profiles of different batches of the same cell lines obtained from distinct labs we show that, despite being generally stable, the genomes of MPNST cell lines accumulate changes over time.

Figure 1 shows the samples analyzed to illustrate two cases of co-evolution of the same cell lines that have been expanded and preserved in independent labs.

The genomic profiles of these two cell lines, ST88-14 and S462, can be seen in **Figure 2** and **Figure 3** respectively. In both figures, a purple shadow highlights the regions with a different genomic profile among the different samples cell batches analyzed.

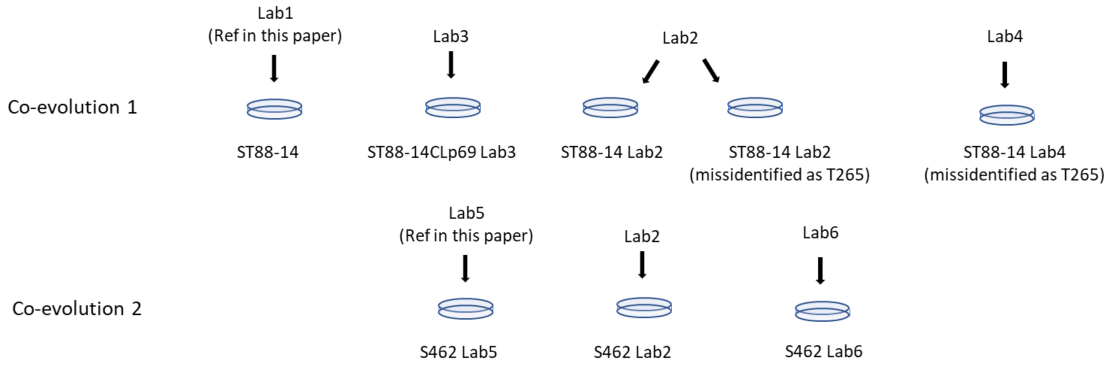

***Figure 1**. Scheme explaining the different samples analyzed by SNP-array to illustrate co-evolution of cell lines expanded and preserved in independent labs.*

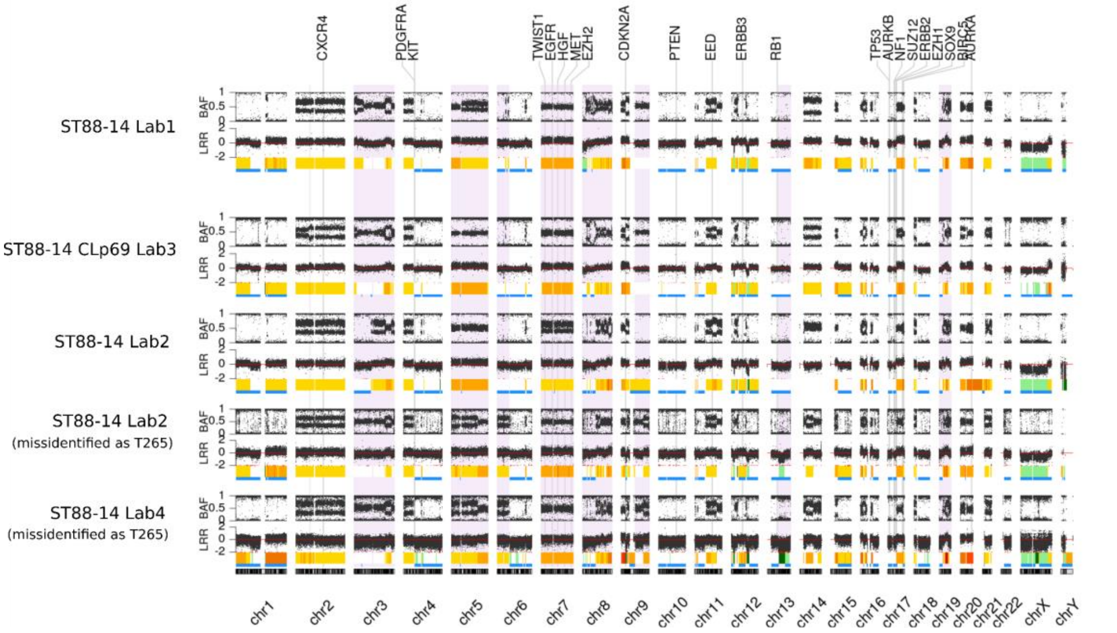

***Figure 2**. SNP-array profiles show the co-evolution of the ST88-14 cell line in samples from different laboratories. A purple shadow highlights the differences between them*

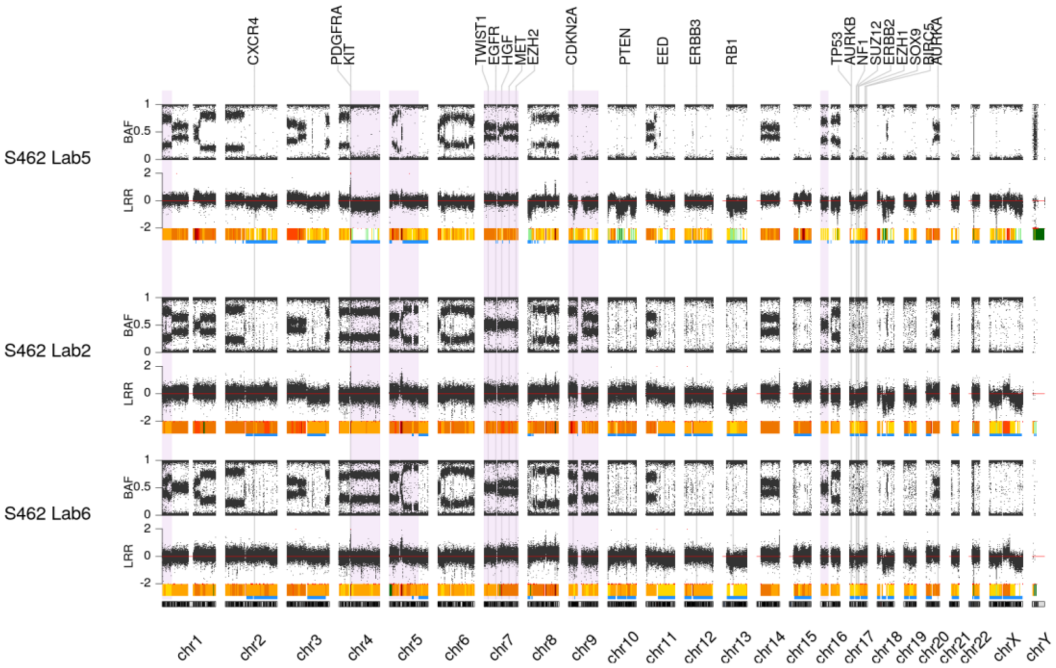

***Figure 3**. SNP-array profiles show the co-evolution of the S462 cell line in samples from different laboratories. A purple shadow highlights the differences between them*

Data S6:

Examples of rearrangements affecting MPNST-related genes

Related to Figure 3 and Figure 4

Breakpoint detection using Integrative Genomic Viewer (IGV)

Integrative Genomic Viewer [1] (IGV) allows us to visualize NGS data mapped against a reference genome. In IGV, we can see the different alteration patterns created by different structural variants (SV). Genomic breakpoints cause a drastic drop in coverage and the presence of soft-clipped reads showing that part of the reads do not match the reference genome at that position. Rearrangements also produce a discordant mapping of reads in a read pair, where each read maps on different chromosomes or unexpected locations in the same chromosome. These discordant read pairs are represented by different colors, so the presence of colored reads close to the breakpoints reinforce the breakpoint identification and provide information about the translocation partner (**Figure 1**).

In the following sections, we show some breakpoints originated from inter-chromosomal rearrangements affecting different genes on different MPNSTs cell lines.

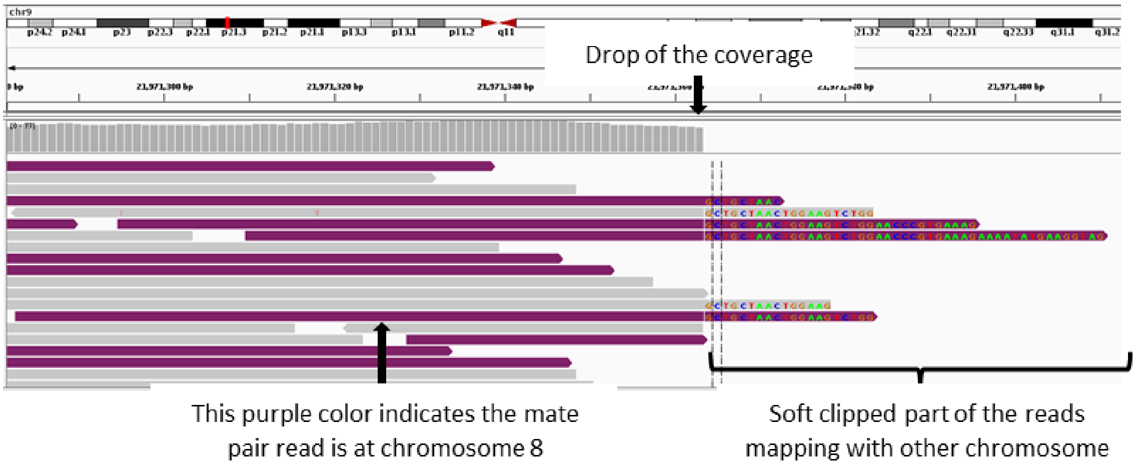

Figure 1. *Screenshot from IGV showing a genomic breakpoint. Annotations point to the effects of breakpoints: coverage drop, soft-clipped reads and colored reads.*

NMS-2

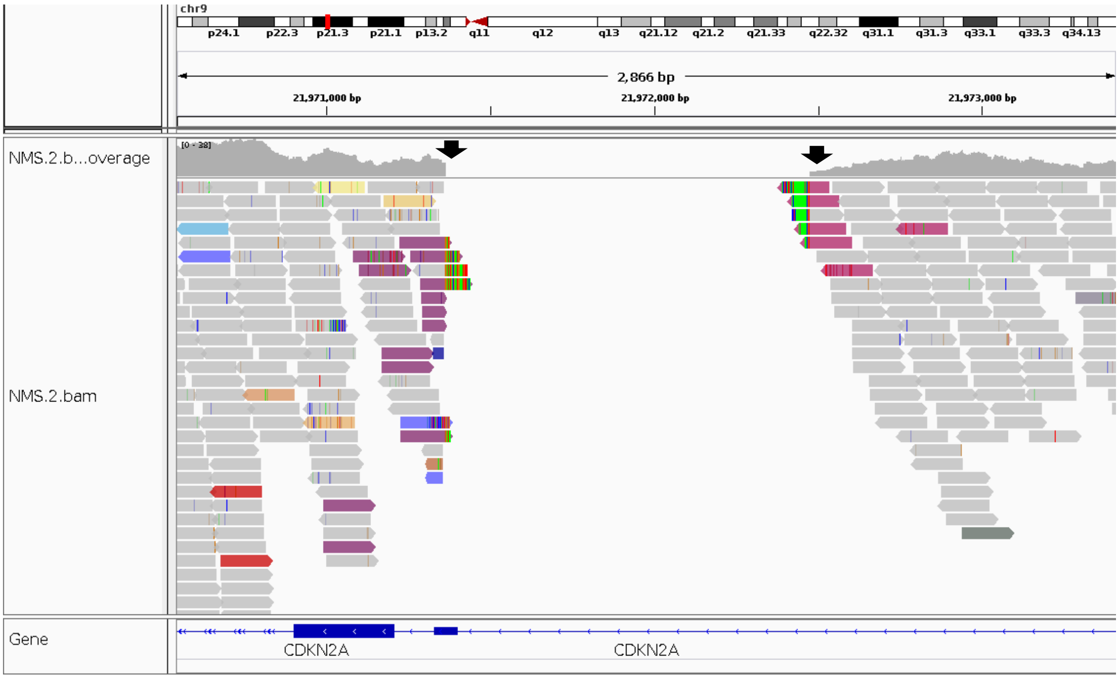

***Figure 2**. Screenshot from IGV showing the breakpoints affecting CDKN2A in NMS-2. Black arrows indicate the positions of two translocation breakpoints, one with chromosome 8 and one with chromosome 20.*

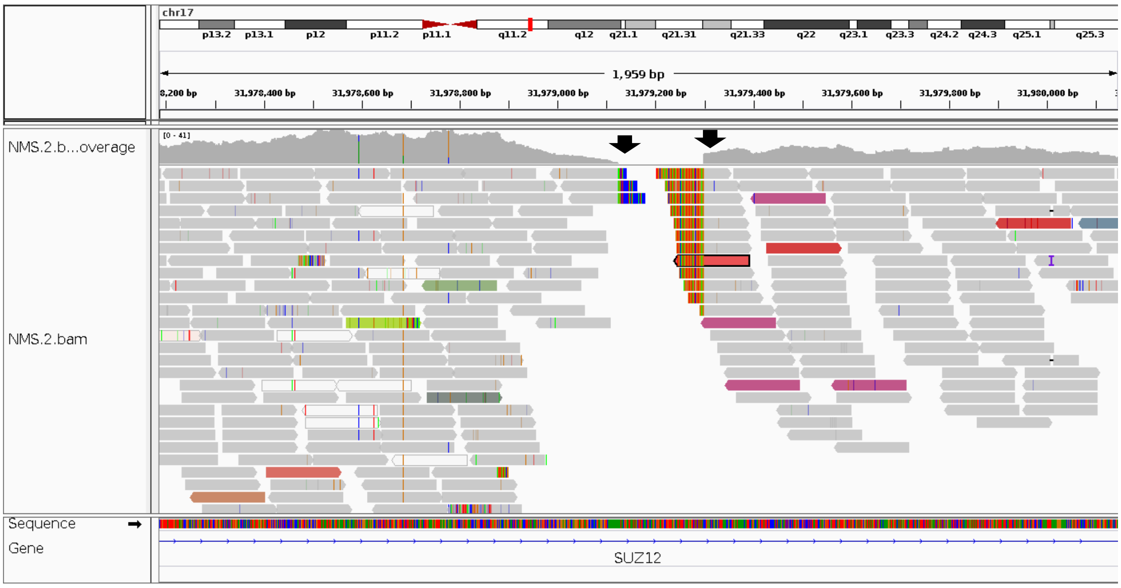

***Figure 3**. Screenshot from IGV showing a translocation of SUZ12 with a telomeric region in NMS-2. Black arrows indicate breakpoint positions*

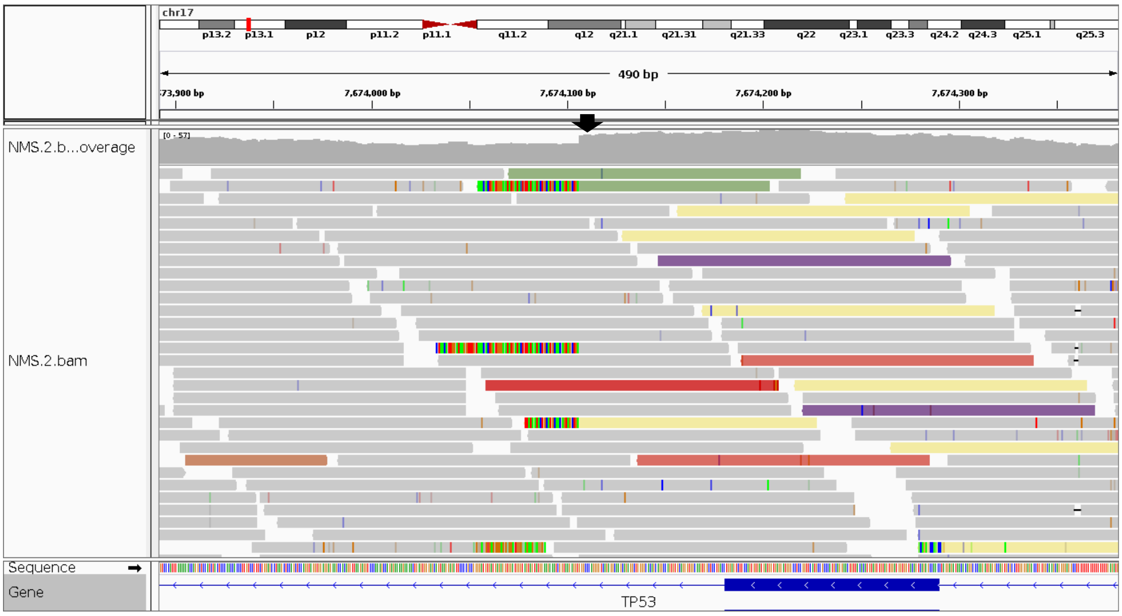

***Figure 4**. Screenshot from IGV showing the breakpoints of a translocation between* TP53 *and chromosome 4 in NMS-2. Black arrow indicates the breakpoint position.*

ST88-14

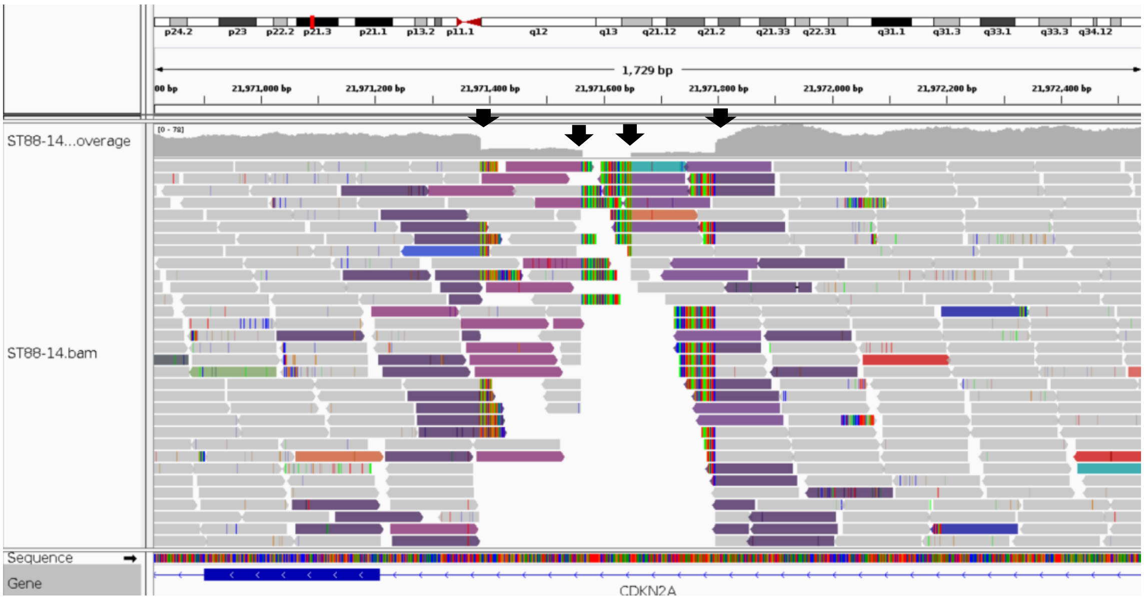

***Figure 5**. Screenshot from IGV showing multiple breakpoints close to exon 2 of CDKN2A in ST88-14. Black arrows indicate breakpoint positions. This is a complex rearrangement involving chromosomes 9, 17 and 19.*

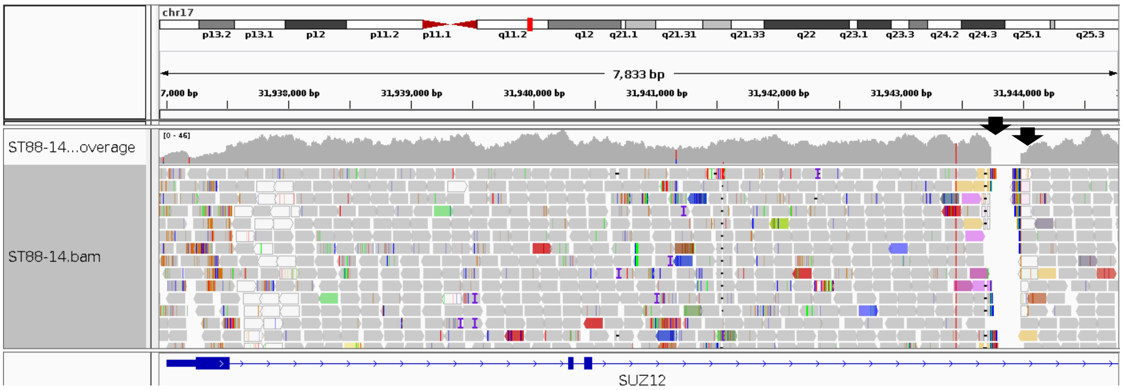

Figure 6. *Screenshot from IGV showing the translocation breakpoints affecting SUZ12 in ST88-14. Black arrows indicate breakpoint positions.*

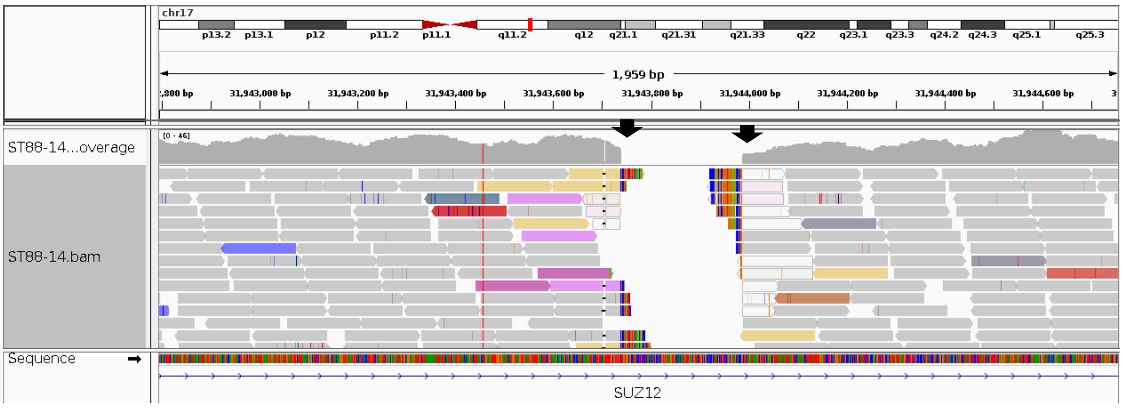

Figure 7. *Screenshot from IGV showing closer image of breakpoints affecting SUZ12 in ST88-14. Black arrows indicate breakpoint positions. The very light colors of reads next to the breakpoint show low-mapping quality since this breakpoint seems to involve repetitive regions in chromosome 21.*

Validation by PCR and Sanger sequencing of some breakpoints.

Inter-chromosomal rearrangements detected by LUMPY or CliffHunteR affecting some of the commonly altered genes in MPNST were validated by PCR and Sanger sequencing. The PCR primers, annealing temperatures and amplicon lengths are summarized in the following table:

Table 1. List of primers used for the breakpoint validation

Name	Sequence (5'->3') Template	Length	Tm	Atm
ST8814_F_chr9-chr17_rigth_CDKN2A	CTGGATAATCTCCCTCAAGACCA	23	59,03	58,79
ST8814_R_chr9-chr17_rigth_CDKN2A	ACAGTGGACAATAAGCACTTGAA	23	58,54	
ST8814_F_chr9-chr8_CDKN2A	AAGCCCCAGGTGTCTAATTACC	22	59,76	59,96
ST8814_R_chr9-chr8_CDKN2A	TGCTCCATTGGTCTTTGTGACT	22	60,16	
NMS_2_F_chr4-chr17_TP53	AGCATGTTAGTCAAGGTAGCCC	22	60,09	60,03
NMS_2_R_chr4-chr17_TP53	TCCTAGGTTGGCTCTGACTGTA	22	59,96	

a

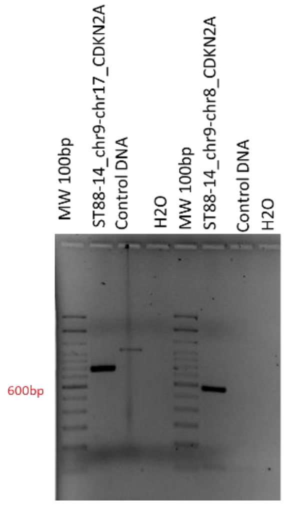

b

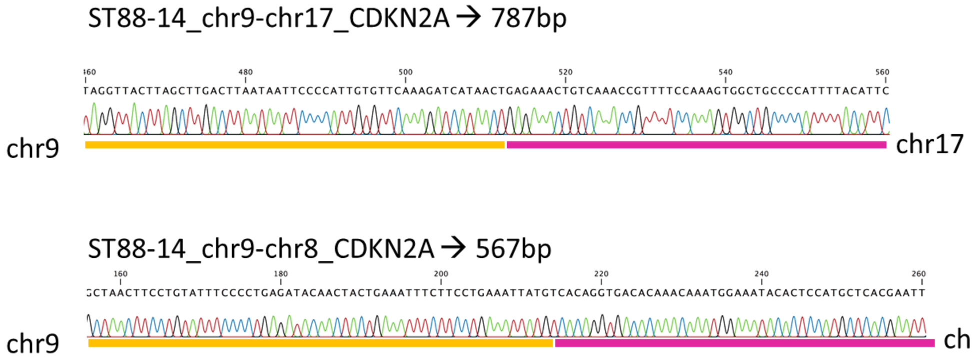

Figure 8. *Validation of two of the breakpoints inactivating CDKN2A in ST88-14 cell line.**(a).** PCR validation. **(b).** Sanger sequencing breakpoint validation*

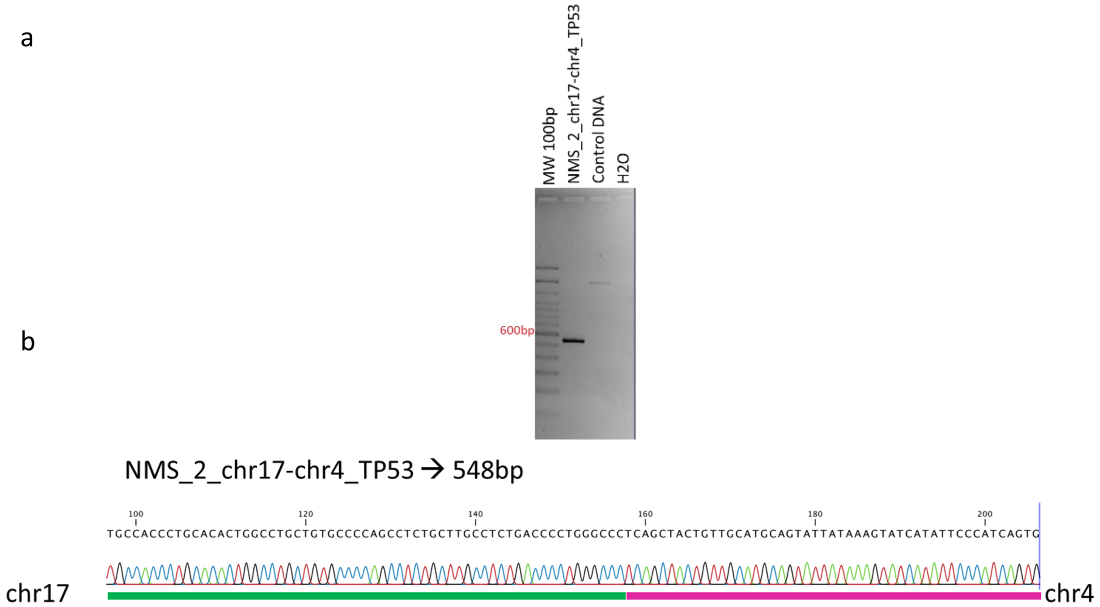

Figure 9. *Validation of the breakpoint affecting TP53 in NMS-2 cell line..(a). PCR validation. (b). Sanger sequencing breakpoint validation*

Validation by RT-PCR and Sanger sequencing of the EML4-ALK fusion gene breakpoints detected in the HS-PSS cell line.

Validation of the breakpoints detected in HS-PSS cell line by WGS data. We used the primers and conditions described previously in Takeuchi et al. (2008)[2].

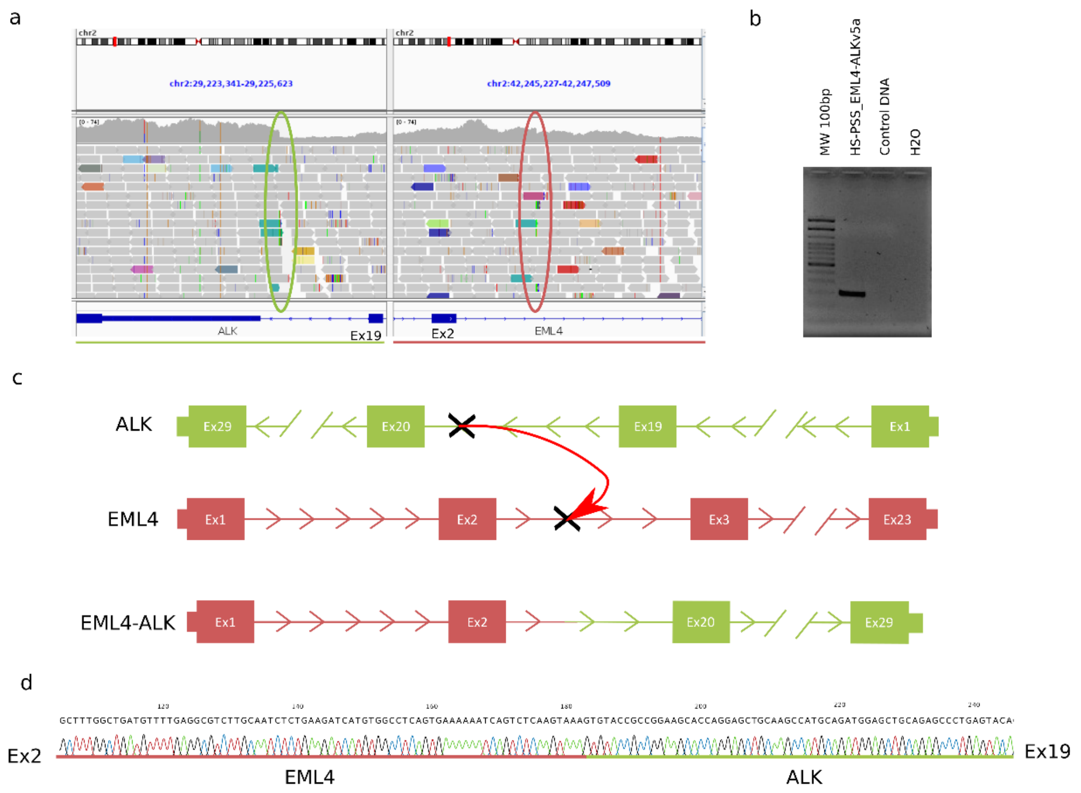

Figure 10. EML4-ALK v5a validation. ***(a)****. IGV screenshot showing the inversion breakpoints.* ***(b)****. RT-PCR gel confirming the amplification of the fusion gene.* ***(c)****. Scheme of the fusion gene generated.* ***(d)****. Sanger sequencing validating the EML4-ALK v5a fusion gene found in HS-PSS cell line.*

References

1. Robinson JT, Thorvaldsdóttir H, Winckler W, Guttman M, Lander ES, Getz G, et al. Integrative genomics viewer. Nature Biotechnology. 2011;29:24–6.

2. Takeuchi K, Choi YL, Soda M, Inamura K, Togashi Y, Hatano S, et al. Multiplex reverse transcription-PCR screening for EML4-ALK fusion transcripts. Clinical Cancer Research. 2008;14:6618–24.
